# Effectiveness and Safety of Iguratimod Monotherapy or Combined With Methotrexate in Treating Rheumatoid Arthritis: A Systematic Review and Meta-Analysis

**DOI:** 10.3389/fphar.2022.911810

**Published:** 2022-08-05

**Authors:** Dan Ouyang, Yuan Zhi Ma, Jie Zou, Yong Long Wang, Zheng Chen, Yu Ying Yang, Bin Zou, Xin Li, Jian Zhong Cao

**Affiliations:** ^1^ School of Chinese Medicine, Hunan University of Chinese Medicine, Changsha, China; ^2^ Hunan Provincial Key Laboratory of Diagnostics in Chinese Medicine, Hunan University of Chinese Medicine, Changsha, China; ^3^ General Surgery Department, University of South China Affiliated Changsha Central Hospital, Changsha, China

**Keywords:** rheumatoid arthritis, systematic review, meta-analysis, methotrexate, iguratimod

## Abstract

**Objectives:** We aimed to estimate the effectiveness and safety of iguratimod (IGU) monotherapy or in combination with methotrexate (MTX) in treating rheumatoid arthritis (RA) to provide an evidence-primarily-based foundation for clinical application.

**Methods:** We conducted a systematic review of the meta-analysis using eight databases and two clinical trial websites searching for randomized controlled trials (RCTs) from conception to 15 March 2022, based on outcomes of patients with RA treated with IGU. The evidence quality assessment of primary outcomes was evaluated by the GRADE tool, and RevMan 5.3 and StataMP 14.0 were used to perform this research.

**Results:** A total of 4302 patients with RA from 38 RCTs was included in this research. Pooled results demonstrated as follows: 1) Compared with methotrexate (MTX) alone, IGU alone was superior in improving ACR20 and DAS28-ESR, while having no significant difference in ACR50 and ACR70 [ACR20: (RR 1.15, 95% CI 1.05–1.27, *p* = 0.004); ACR50: (RR 0.97, 95% CI 0.66–1.44, *p* = 0.88); ACR70: (RR 0.92, 95% CI 0.45–1.90, *p* = 0.83); DAS28-ESR: mean difference (MD) −0.15, 95% CI −0.27 to −0.03, *p* = 0.01]. 2) Compared with MTX alone, IGU + MTX was more effective in improving ACR20, ACR50, ACR70, and DAS28-ESR. [ACR20: (RR 1.24, 95% CI 1.14–1.35, *p* < 0.00001); ACR50: (RR 1.96, 95% CI 1.62–2.39, *p* <0.00001); ACR70: (RR 1.91, 95% CI 1.41–2.57, *p* < 0.0001)]; [DAS28-ESR: (MD) −1.43, 95% CI −1.73 to −1.12, *p* < 0.00001]. 3) Compared with MTX + leflunomide (LEF), ACR20, ACR50, ACR70, and DAS28-ESR of IGU + MTX had no significant difference [ACR20: (RR 1.06, 95% CI 0.94–1.19, *p =* 0.38); ACR50: (RR 1.10, 95% CI 0.66–1.84, *p* = 0.72); ACR70: (RR 1.20, 95% CI 0.45–3.20, *p* = 0.71); DAS28-ESR: (MD −0.02, 95% CI −0.13 to −0.10, *p* = 0.77)]. 4) Compared with MTX + hydroxychloroquine (HCQ), IGU + MTX was superior in improving DAS28-ESR (MD −2.16, 95% CI −2.53 to −1.79, *p* < 0.00001). 5) Compared with MTX + tripterygium glycosides (TGs), IGU + MTX was more effective in improving DAS28-ESR (MD −0.94, 95% CI −2.36 to 0.48, *p* = 0.19). 6) There were no significant differences in adverse events (AEs) between the groups of IGU vs. MTX (RR 0.96, 95% CI 0.71–1.31, *p* = 0.80), IGU + MTX vs. MTX (RR 1.10, 95% CI 0.90–1.35, *p* = 0.34), IGU + MTX vs. MTX + HCQ (RR 0.64, 95% CI 0.29–1.42, *p* = 0.27), and IGU + MTX vs. MTX + TGs (RR 0.75, 95% CI 0.28–2.02, *p* = 0.57). The incidence of AEs in the IGU + MTX group was lower than the MTX + LEF group (RR 0.83, 95% CI 0.71–0.98, *p* = 0.03).

**Conclusion:** Compared to the MTX alone subgroup, IGU alone offers clear advantages in improving ACR20 and DAS28-ESR, despite the insufficient evidence for DAS28-ESR findings. IGU + MTX shows clear benefits in improving ACR20, ACR50, ACR70, and DAS28-ESR scores compared to standard therapies. When the intervention (IGU alone or IGU + MTX) lasted for 52 weeks, it demonstrated superior efficacy in improving ACR20 of patients without prominent adverse events. Notably, IGU or IGU + MTX has apparent advantages in improving ACR20 of first-visit RA, and IGU + MTX has obvious advantages in improving DAS28-ESR of refractory RA. Furthermore, IGU + MTX does not increase the risk of leukopenia, but it can decrease the risk of liver function tests (LFTs), regardless of the age or the stage of RA.

**Clinical Trial Registration:** https://www.crd.york.ac.uk/PROSPERO/#recordDetails, identifier CRD42022295217

## 1 Introduction

Rheumatoid arthritis (RA) is an autoimmune disease that alternates between progressing and stabilizing owing to abnormal immune response. The disease’s etiology is still unknown, and the pathogenesis is complicated (Meehan et al., 2021). The primary pathological foundation is erosive synovitis, which gradually leads to angiogenesis and pannus formation (Matsui et al., 2009), and finally leads to joint bone and cartilage destruction, resulting in joint deformity and dysfunction (Auréal et al., 2020). Patients with advanced-stage cancer have a significantly lower quality of life and are more likely to have labor loss, paralysis, and despair (Hunter et al., 2017; Otón and Carmona, 2019). The overall goal of RA treatment is to control symptoms and prevent disease progression. It encourages early treatment and treat-to-target to achieve clinical remission or dropped disease activity. Currently, disease-modifying anti-rheumatism drugs (DMARDs), nonsteroidal anti-inflammatory medicines (NSAIDs), glucocorticoids, and other medications are used to treat RA. There are four major categories of DMARDs, traditional synthesis (csDMARDs), targeted synthesis (tsDMARDs), biological original research (boDMARDs), and biosimilars (bsDMARDs). Traditional DMARDs include methotrexate (MTX), leflunomide (LEF), and tripterygium glycosides (TGs) (Burmester and Pope, 2017; Ferro et al., 2017). Targeted DMARDs include anti-TNF-α blockers, anti-IL antibodies, and etanercept (Burmester and Pope, 2017; Liu et al., 2018).

Attributable to the complicated pathophysiology of RA, clinical therapy with first-line drugs such as MTX does not always meet therapeutic requirements. The international guidelines recommend that when a single DMARD treatment does not meet the criteria, combination of DMARDs improve the curative effects (Singh et al., 2016; Smolen et al., 2017; Lau et al., 2019). Guidelines of China in 2018 also mentioned that for patients who do not accord with standard MTX alone, it is recommended to use MTX in combination with another DMARD (Chinese Rheumatology Association, 2018). IGU is a new type of small-molecule compound, which mainly regulates the immune system, inhibits T-cell and B-cell differentiation, reduces inflammatory factors, improves the function of joint swelling, and is widely used in China and Japan (Jiang et al., 2020). Multiple studies have demonstrated the superior efficacy of IGU alone or in combination with MTX in treating RA with acceptable safety (Ishiguro et al., 2013; Shi et al., 2015; Du et al., 2021).

Furthermore, it was shown to be beneficial for refractory RA and elderly RA without noticeable adverse reactions (AEs) (Cao et al., 2018; Ju et al., 2020; Li and Sun, 2021). Recently, a large multicenter randomized controlled trial was conducted to evaluate the effectiveness and safety of IGU alone or in combination with MTX. Du F et al. discovered that IGU alone or IGU + MTX was superior to MTX at week 52 with a higher ACR20 response and adequate security (Du et al., 2012). Hu et al. (2021) conducted a systematic review and meta-analysis of IGU alone or IGU + MTX treatment, but only 23 RCTs were included. Furthermore, RCTs from additional clinical research centers demonstrated the effectiveness and safety of IGU alone or in combination with MTX in treating RA (Du et al., 2021; Niu et al., 2021; Zhao, 2021). As a result, for the first time, this study could conduct a systematic review and meta-analysis of the efficacy and safety of IGU alone or combined with MTX, providing an evidence-based foundation, new direction for clinical treatment, and new research direction for RCTs in the future.

## 2 Materials and Methods

### 2.1 Protocol

This meta-analysis was performed strictly by the protocol registered in PROSPERO (CRD42022295217) and the PRISMA guidelines (Supplementary Table S1).

### 2.2 Literature Search Strategy

We searched eight databases, the Chinese Biomedical Medicine (CBM), China National Knowledge Infrastructure (CNKI), Wanfang Med Database, China Science and Technology Journal Database (VIP), PubMed, Cochrane Central Register of Controlled Trials (CENTRAL), Embase, Web of Science, as well as two clinical trial websites, the ClinicalTrials.gov and Chinese Clinical Trial Registry, from conception to 15 March 2022. The search strategy is shown in Supplementary Table S2.

### 2.3 Screening Standard

#### 2.3.1 Inclusion Criteria

(1) Participants: All patients over 18 with specific diagnostic criteria for RA (Arnett, 1988; Aletaha et al., 2010), with a balanced baseline and comparability.
(2) Intervention and control: The treatment of the experimental group included IGU monotherapy or combined with Western medicine, lifestyle, or exercise. The control group included placebo and Western medicine but without IGU.
(3) Outcomes: Primary endpoints are ACR20/50/70, 28 joint disease activity score-ESR (DAS28-ESR), and adverse events (AEs). Secondary endpoints are tender joint count-28 (TJC-28), swollen joint count-28 (SJC-28), morning stiffness (min), visual analog scale (VAS), global patient assessment (PGA), global physician assessment (EGA), Health Assessment Questionnaire (HAQ), erythrocyte sedimentation rate (ESR), C-reactive protein (CRP), anti-cyclic citrullinated peptides (anti-CCP), rheumatoid factors (RF).

#### 2.3.2 Exclusion Criteria

(1) Repeated publications
(2) Review and meta-analysis
(3) Animal or cell-based experiments
(4) No RCTs
(5) Obscure data
(6) Full text cannot be obtained
(7) Case reports.

### 2.4 Data Extraction and Risk of Bias Assessment

The literature search was conducted independently by two researchers and data extraction by five independent reviewers according to the screening criteria, followed by data cross-check. Any discrepancies were resolved by consensus or consultation with other reviewers.

Literature quality was assessed by the bias risk assessment criteria of the Cochrane Collaboration network (Deeks et al., 2021a). The assessment is as follows: 1) random assignment method; 2) allocation concealment; 3) blind method; 4) integrity of data; 5) selective reporting; 6) other bias.

### 2.5 Statistical Analysis

RevMan 5.3 and StataMP 14.0 software were used for this meta-analysis. First, a heterogeneity test was carried out. I^2^ and chi-square tests evaluated significance and heterogeneity. If the heterogeneity test results were not statistically significant (*p* > 0.1, I^2^ ≤ 50%), choose the fixed-effect model. Otherwise, choose the random-effects model (Deeks et al., 2021b). To identify the cause of the heterogeneity, the subgroup analysis was carried out based on the control group's intervention. The dichotomous variables were calculated as odds ratio (OR) or risk ratio (RR), and continuous variables as mean difference (MD) or standard mean difference (SMD). All effect sizes were expressed as 95% confidence interval (95% CI). The meta-analysis test level was *p* = 0.05. For primary outcomes, the publication bias was assessed by Egger's and Harbord's texts. *p* > 0.1 was considered free of publication bias. For all outcomes, sensitivity analyses were evaluated by observing the changes of RR (OR) and MD (SMD) after changing the effect model. According to the GRADE manual (GRADEpro, 2015), the GRADE tool was used to grade the quality of the evidence (Schünemann, 2013).

## 3 Results

### 3.1 Literature Screening Results

**FIGURE 1:**
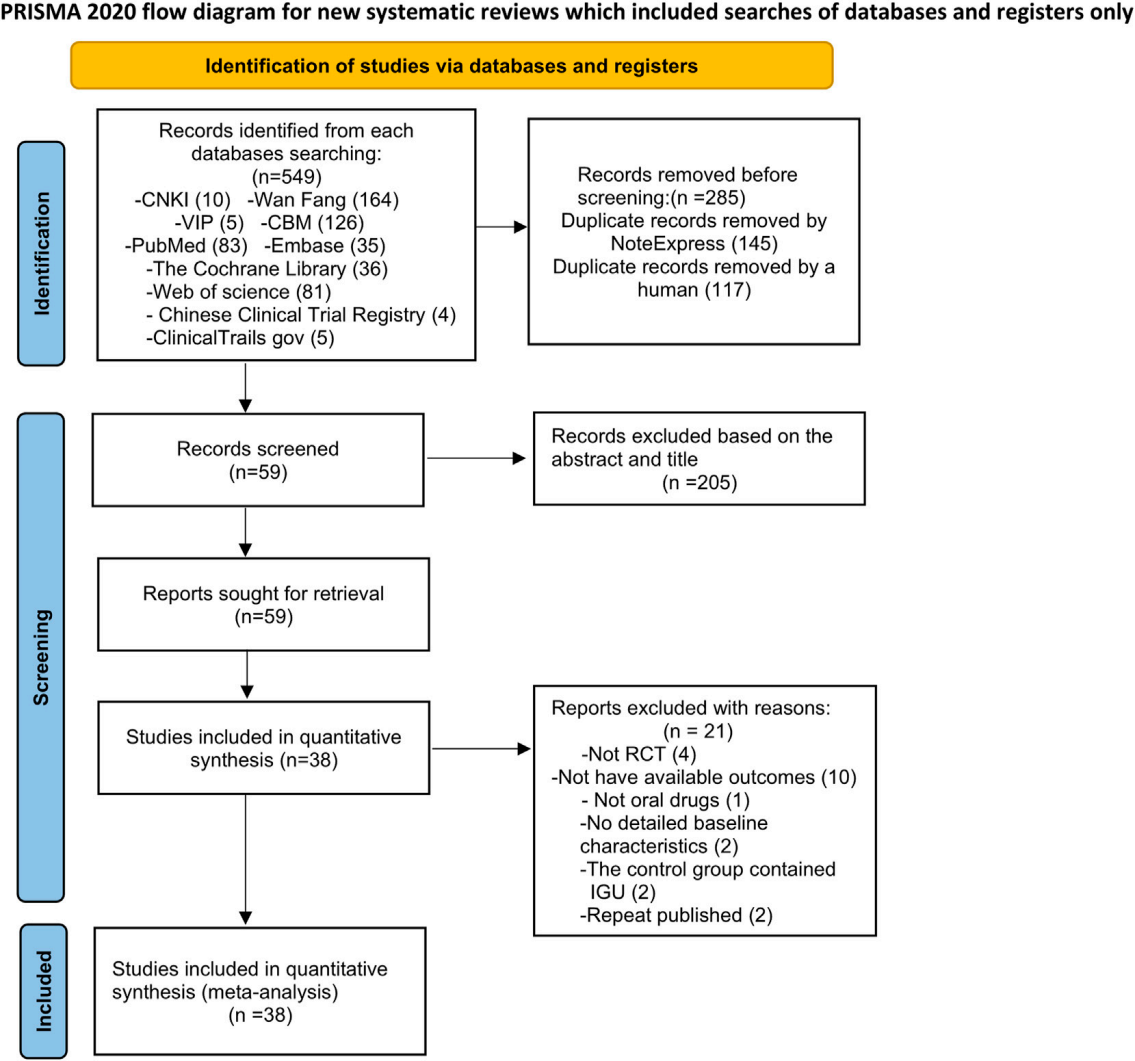
Flow diagram.

A total of 549 relevant studies were initially retrieved, and 38 articles were finally included according to the inclusion and exclusion criteria (Figure 1).

### 3.2 Basic Characteristics of the Included Literature

This study ended up including 38 RCTs involving 4302 participants. The number of people who took part in the IGU alone ranged from 30 to 297, while those who took part in the IGU + MTX study mainly were between 27 and 305. Interventions in the control group were predominantly MTX-only. The control group of Ma et al. (2017); Zhu (2017); Tian et al. (2020); Niu et al. (2021); and Meng et al. (2015) used MTX + LEF; the control group of Liu et al. (2020) and Chen and Hu (2021) used MTX + HCQ; the control group of Mo et al. (2018) and Li and Sun (2021) used MTX + TGs. In all studies, there was no statistical significance in the gender, age, and severity of the disease between the two groups before treatment (Table 1).

**TABLE 1:** The characteristics of the included studies.

Source	Mean Age (years)		Disease duration (years)		Baseline DAS28		Sample Size (Female/Male)		Intervention and dose	Main Outcomes	Age Range	Treatment duration	Disease Stage
	Trial Group	Control Group	Trial Group	Control Group	Trial Group	Control Group	Trial Group	Control Group					
Ishiguro N 2015	54.8 ± 9.9	53.5 ± 10.0	4.48 ± 0.83	4.48 ± 0.88	4.87 ± 0.89	4.97 ± 0.86	164 (134/30)	88 (70/18)	IGU 25 mg qd-bid + MTX 6/8 mg qw VS. MTX 6/8 mg qw + PLA	ACR50/70, DAS28, HAQ, RF	20<years<70	24w	-
Shi X D 2015	48.9 ± 12.2	48.4 ± 10.2	7.5 ± 4.8	7.1 ± 6.6	5.2 ± 1.3	5.2 ± 1.9	30/30 (42/18)		MTX 10–12.5 mg qw + IGU 25 mg bid VS. MTX 10–12.5 mg qw	ACR20/50/70, DAS28, VAS, PGA, EGA, HAQ, SJC, TJC, ESR, CRP, AEs	22 < years<70	24w	First-visit
Bai Q H 2015	-	-	-	-	-	-	50/50 (76/24)		IGU 25 mg bid + MTX 10 mg qw VS. MTX 10 mg qw	ACR20/50, AEs	22 < years<62	12w24w	-
Mo H 2015	31.8 ± 8.5	31.9 ± 8.6	5.6 ± 1.8	5.5 ± 1.9	-	-	30 (22/8)	30 (21/9)	IGU 25 mg bid + MTX 15 mg qw VS. MTX 15 mg qw	ACR20/50/70, ESR, CRP, RF, Anti-CCP, AEs	18 < years<72	12w	-
Xiong Y M 2015	56 ± 12	51 ± 13	-	-	-	-	30 (24/6)	28 (21/6)	IGU 25 mg bid + MTX 10 mg qw VS. MTX 10 mg qw	DAS28, ESR, CRP, RF, Anti-CCP	21 < years<68	12w,24w	-
Xu B J 2015	46.10 ± 17.09	A:43.28 ± 10.46 B:44.71 ± 9.32	4.7 ± 0.58	A:4.34 ± 0.78 B:4.23 ± 0.94	-	-	40 (23/17)	A:38 (24/14) B:32 (20/12)	IGU 25 mg bid + MTX 7.5–20 mg qw VS. IGU 25 mg bid VS. MTX 7.5–20 mg qw	PGA, Morning stiffness, TJC, SJC, ESR, CRP, RF, AEs	23 < years<72	52w	-
Wang Z J 2016	48.71 ± 8.77	47.68 ± 7.67	8.31 ± 2.61	7.28 ± 2.58	5.95 ± 1.64	6.48 ± 1.92	44 (29/15)	43 (21/6)	IGU 25 mg bid + MTX 15 mg qw VS. MTX 15 mg qw	DAS28, AEs	35 < years<70	24w	Refractory
Meng D Q 2016	41.6 ± 20.3	45.1 ± 19.2	-	-	6.40 ± 1.90	5.97 ± 1.62	30 (26/4)	30 (23/7)	IGU 25 mg bid + MTX 15 mg qw VS*.* MTX 15 mg qw	DAS28, AEs	18 < years<65	16w	Refractory
Xu L M 2017	46.34 ± 2.29	46.19 ± 2.57	-	-	6.92 ± 2.91	6.72 ± 2.94	42 (23/19)	41 (22/19)	IGU 25 mg bid + MTX 7.5–20 mg qw VS. MTX 7.5–20 mg qw	DAS28, Morning stiffness time, ESR, CRP	21 ≤ year≤70	52W	-
Cao L N 2018	67.5 ± 3.2	A:68.0 ± 2.8 B:68.5 ± 2.0	-	-	4.99 ± 0.17	A:4.98 ± 0.27 B:4.91 ± 0.30	43 (23/19)	A:30 (15/15) B:30 (20/10)	IGU 25 mg bid + MTX 10–12.5 mg qw VS. IGU 25 mg bid VS. MTX 10–12.5 mg qw	DAS28, HAQ	61 ≤ year≤78	24w	-
Zhao H N 2018	47.20 ± 3.40	46.90 ± 3.60	4.28 ± 0.36	4.23 ± 0.34	6.9 ± 2.8	6.8 ± 2.9	36 (24/12)	36 (23/13)	MTX 10 mg qw + IGU 25 mg bid VS. MTX 10 mg qw	Morning stiffness, ESR, CRP, AEs	23 < years<75	12w	-
Ju Y J 2020	42.31 ± 13.78	41.87 ± 13.94	4.72 ± 0.43	4.56 ± 0.58	6.46 ± 2.24	6.27 ± 2.12	58 (23/35)	58 (25/33)	IGU 25 mg bid + MTX 10–15 mg qw-biw VS. MTX 10–15 mg qw-biw	DAS28, ESR, CRP, RF, AEs	20.7 < years<69.3	24w	Refractory
Xiong M L 2020	48.21 ± 3.78	48.33 ± 5.93	1.98 ± 0.43	1.54 ± 0.39	-	-	51 (29/22)	51 (30/21)	IGU 25 mg bid + MTX 10–15 mg qw VS. MTX 10–15 mg qw	Morning stiffness, SJC, TJC, AEs	26 < years<65	24w	-
Jing J 2020	50.03 ± 9.96	49.87 ± 9.78	6.13 ± 1.53	6.26 ± 1.61	6.31 ± 0.85	6.29 ± 0.83	46 (25/21)	46 (26/20)	IGU 25 mg bid + MTX 10–12.5mg/w VS. MTX 10–12.5mg/w	DAS28, Morning stiffness, ESR, CRP, TJC, SJC, RF	31 < years<73	24w	-
Xie L 2018	62.89 ± 4.57	62.74 ± 3.96	6.41 ± 2.16	7.35 ± 1.87	6.75 ± 1.69	6.84 ± 1.87	39 (27/12)	39 (25/14)	IGU 25 mg bid + MTX 10–15 mg qw-biw VS. MTX 10–15 mg qw-biw	DAS28, AEs	25 < years<71	16w	Refractory
Qi D X 2019	-	-	-	-	-	-	40/40/40 (Unknown)		IGU 25 mg bid + MTX 7.5–10 mg qw VS. IGU 25 mg bid VS. MTX 7.5–10 mg qw	ACR20/50/70, PGA, EGA, HAQ, TCJ, SJC, ESR, CRP, RF, Anti-CCP, AEs	25<years<65	24w	-
Wang L H 2019	48.13 ± 6.40	47.83 ± 6.37	5.60 ± 0.70	5.41 ± 0.72	6.30 ± 0.88	6.27 ± 0.85	47 (23/23)	46 (25/22)	IGU 25 mg bid + MTX 15 mg qw VS. MTX 15 mg qw	DAS28, TJC, SJC, ESR, CRP, RF, AEs	30 < years<80	24w	-
Yan K H 2019	43.74 ± 4.83	43.58 ± 4.6	11.54 ± 2.36	11.56 ± 2.41	-	-	40 (28/12)	40 (29/11)	IGU 25 mg bid + MTX 15 mg qw VS. MTX 15 mg qw	ACR20/50/70, AEs	22 < years<69	12w/9w	-
Zhao W Z 2021	48.41 ± 6.39	48.36 ± 6.36	8.44 ± 2.39	8.39 ± 2.36	6.28 ± 1.85	6.31 ± 1.86	52 (26/26)	52 (28/24)	IGU 25 mg bid + MTX 10 mg qw VS. MTX 10 mg qw	DAS28, Morning stiffness, TJC,SJC,ESR,RF, AEs	42 < years<55	24w	-
Duan X 2015	48.9 ± 12.2	48.4 ± 10.2	7.5 ± 4.8	7.1 ± 6.6	5.2 ± 1.3	5.2 ± 0.9	30/30 (42/18)		IGU 25 mg bid + MTX 10–12.5 mg qw VS. MTX 10–12.5 mg qw	ACR20/50/70, VAS, PGA, EGA, TJC, SJC, ESR, CRP, AEs		24w	-
Xia Z 2016	46.63 ± 10.61	-	-	-	3.82 ± 0.07	A:3.98 ± 0.09 B:3.79 ± 0.08	44/38/39 (107/24)		IGU 25 mg bid + MTX 10 mg qw VS. IGU 25 mg bid VS. MTX 10 mg qw	Morning stiffness. SJC, TJC, ESR, CRP	46.63 ± 10.61	24w	-
Li S Y 2019	45.25 ± 2.78	45.425 ± 2.57	7.26 ± 0.82	7.21 ± 0.80	-	-	40 (13/27)	40 (15/25)	IGU 25 mg bid + MTX 7.5 m g-15 mg qw VS. MTX 7.5 m g-15 mg qw	Morning stiffness, ESR, CRP, AEs	32 < years<79	4w	-
Du F 2021	48.37 ± 0.69	A:46.87 ± 0.67 B:47.63 ± 10.70	11.67 ± 7.27	A:11.67 ± 7.16 B:11.60 ± 7.98	5.103 ± 0.956	A:5.084 ± 0.994 B: 5.102 ± 0.979	305 (238/67)	A:297 (230/67) B:293 (232/61)	IGU 25 mg bid + MTX 10–15 mg qw VS. IGU 25 mg bid VS. MTX 10–15 mg qw	ACR20,AEs		52w	First-visit
Chen X Y 2018	50.3 ± 6.8	7.2 ± 1.5				-	-	40/40 (56/24)	IGU 25 mg bid + MTX 10–12.5 mg qw VS. MTX 10–12.5 mg qw	ACR20/50/70, AEs	31 < years<71	4w,8w	-
Xia Z B 2017	54.50 ± 4.50	55.25 ± 4.75	-	-	-	-	27 (12/15)	28 (12/16)	IGU 25 mg bid + MTX 10 mg qw-biw VS. MTX 10 mg qw-biw	ESR, CRP, RF		8w	Refractory
Zhao L 2017	45.97 ± 10.75	A:46.46 ± 11.01 B:46.31 ± 10.89	-	-	7.40 ± 0.67	A: 7.12 ± 0.63 B:7.07 ± 0.50	29 (26/3)	A:34 (27/7) B:33 (28/5)	IGU 25 mg bid + MTX 10 mg qw VS. IGU 25 mg bid + PLA 10 mg qw VS. MTX 10 mg qw + PLA 25 mg bid	DAS28-ESR、VAS、PGA、EGA、HAQ、ESR、CRP	20 < years<69	24w	-
Chen B X 2021	52.73 ± 3.39	53.13 ± 3.64	9.23 ± 1.52	9.94 ± 1.73	-	-	30 (18/12)	30 (20/10)	IGU 25 mg bid + MTX 10 mg qw VS. MTX 10 mg qw + HCQ 200 mg bid	CRP, AEs	41 < years<70	24w	-
Liu C L 2020	44.4 ± 11.2	46.5 ± 12.8	8.2 ± 4.1	5.7 ± 4.2	6.75 ± 2.09	6.78 ± 2.13	73 (40/33)	73 (30/43)	IGU 25 mg bid + MTX 10–15 mg qw VS. MTX 10–15 mg qw + HCQ 200 mg bid	DAS28, TJC, SJC, ESR, CRP, RF, AEs	30 < years<65	24w	-
Tian X P 2020	50 ± 10	49 ± 11	6.08 ± 6.25	6.75 ± 7.33	-	-	107 (87/20)	100 (90/10)	IGU 25 mg bid + MTX 10 mg qw PLA 20 mg qd VS. MTX 10 mg qw + LEF 20 mg qd + PLA 25 mg qd	ACR20/50/70, HAQ, TJC, SJC, ESR, CRP, AEs	18 < years<70	52w	-
Zhu L J 2017	67.2 ± 3.0	66.8 ± 3.1	2.7 ± 0.5	2.8 ± 0.4	-	-	42 (22/20)	42 (23/19)	IGU 25 mg bid + MTX 10 mg qw VS. LEF 20 mg qd + MTX 10 mg qw	DAS28, Morning stiffness, SJC, TJC, AEs	67.2 ± 3.0 66.8 ± 3.1	24w	First-visit
Ma C 2017	64.41 ± 6.21	6.11 ± 3.41	64.22 ± 5.81	5.95 ± 3.54	-	-	32 (22/10)	32 (23/9)	IGU 25 mg bid + MTX 10–15 mg qw VS. LEF 20 mg qd + MTX 10–15 mg qw	TJC, SJC, ESR, CRP, AEs	45 < years<89	24w	-
Niu M 2021	48.16 ± 10.26	49.08 ± 11.13	6.27 ± 3.21	6.57 ± 3.35	5.23 ± 0.86	5.21 ± 0.79	64 (35/29)	64 (33/31)	IGU 25 mg bid + MTX 10 mg qw VS. LEF 20 mg qd + MTX 10 mg qw	DAS28, VAS, Morning stiffness, TJC, SJC, AEs	30 < years<65	24w	-
Meng D Q 2015	44.2 ± 20.5	41.7 ± 22.8	-	-	6.53 ± 1.65	6.37 ± 1.89	33 (29/4)	33 (26/7)	IGU 25 mg bid + MTX 10mg/w VS. MTX 10mg/w + LEF 10 mg qd	ACR20/50/70, DAS28, AEs	44.2 ± 20.5 41.7 ± 22.8	8w,16w	Refractory
Mo M L 2018	45 ± 11.56	43.30 ± 10.25	0.75 ± 0.58	0.82 ± 0.54	6.65 ± 1.78	6.78 ± 1.55	30 (22/8)	30 (24/6)	IGU 25 mg bid + MTX 10 mg qw VS. MTX 10 mg qw + Tripterygium Glycosides 20 mg tid	DAS28, ESR, CRP, Anti-CCP, RF, AEs	31 < years<57	4w,8w,12w	-
Xia N 2020	3.73 ± 2.78	3.62 ± 2.45	4.20 ± 1.41	4.17 ± 1.22	-	-	50 (39/11)	50 (37/13)	IGU 25 mg bid + MTX 7.5–15 mg qw VS. MTX 10 mg qw + Tripterygium Glycosides 1–1.5 mg/(kg.d) tid	TJC, SJC, CRP, ESR	41 < years<68	12w	-
Li J Y 2021	45.32 ± 7.44	7.56 ± 8.13	4.34 ± 2.19	4.12 ± 2.85	7.21 ± 1.25	7.58 ± 1.63	30 (22/8)	30 (19/11)	IGU 25 mg bid + MTX 10 mg qw VS. MTX 10 mg qw + Tripterygium Glycosides 1–1.5 mg/(kg.d) tid	DAS28, Morning stiffness, SJC, TJC, ESR, CRP, RF, AEs	45.32 ± 7.44 47.56 ± 8.13	24w	Refractory
Yang C X 2016	45.1 ± 9.1	41.9 ± 9.2	-	-	-	-	40 (16/24)	40 (12/28)	IGU 50 qd VS. MTX 10 mg qw	ACR20/50/70	39 < years<68	12w	-
Zhu H 2019	46.6 ± 5.9	64.4 ± 3.9	-	-	5.39 ± 0.70	5.46 ± 0.70	30 (25/5)	30 (27/3)	IGU 25 mg bid VS. MTX10 mg qw	ACR20/50/70, AEs	66.6 ± 5.9 64.4 ± 3.9	4w,12w,24w	-

### 3.3 Risk of Bias Assessment

#### 3.3.1 Random Sequence Generation and Allocation Concealment

Random allocation was mentioned in all of the included articles, with 15 RCTs of them mentioned the random number table method (Mo and Ma, 2015; Shi et al., 2015; Ma et al., 2017; Zhu, 2017; Cao et al., 2018; Chen, 2018; Mo et al., 2018; Li, 2019; Jing et al., 2020; Ju et al., 2020; Liu et al., 2020; Xia et al., 2020; Xiong and Geng, 2020; Niu et al., 2021; Meng et al. (2015). Two RCTs mentioned the two-color ball randomized method (Zhao and Hao, 2018; Zhao, 2021). Xu et al. (2017) mentioned the method of the random drawing, and Tian et al. (2020) mentioned the system of random regrouping. We classified these studies as low risk of bias. The remaining 17 RCTs did not describe the random sequence generation and were classified as unclear risk of bias. Tian et al. (2020) referred to the "double-dummy" method to make the pills similar in number and appearance; we considered this allocation concealment and classified it as low risk of bias. The other RCTs did not state whether allocation concealment was made, so we assessed the risk of bias as unclear.

#### 3.3.2 Blinding

Tian et al. (2020) and Ishiguro et al. (2013) used a double-blind method, so they were considered to be a low risk of bias. Other RCTs did not state whether they used blinding. Most of their primary outcome indicators are subjective evaluation, which was quickly likely to be affected by the lack of a blinding method. Therefore, they were evaluated as high risk of bias.

#### 3.3.3 Incomplete Outcome Data and Selective Outcome Reporting

Xia et al. (2016) and Zhao et al. (2017) had incomplete outcome data. There was an imbalance in numbers and reasons for missing outcome data across intervention groups, so we evaluated the risk of bias as high. The other RCTs did not have incomplete results, and we assessed the risk of bias as low. The evaluation method mentioned the morning stiffness, TJC-28, SJC-28, RF, and ESR but didn’t report the results Chen and Hu (2021). Ma et al. (2017) missing the results of DAS28-ESR. Mo et al. (2018) missing the results of VAS, PGA, EGA, morning stiffness, TJC-28, and SJC-28. Therefore, we thought they had selective outcome reporting and evaluated the risk of bias as high. The remaining RCTs didn't have selective outcome reporting and were evaluated as low risk.

#### 3.3.4 Other Possible Bias

**FIGURE 2:**
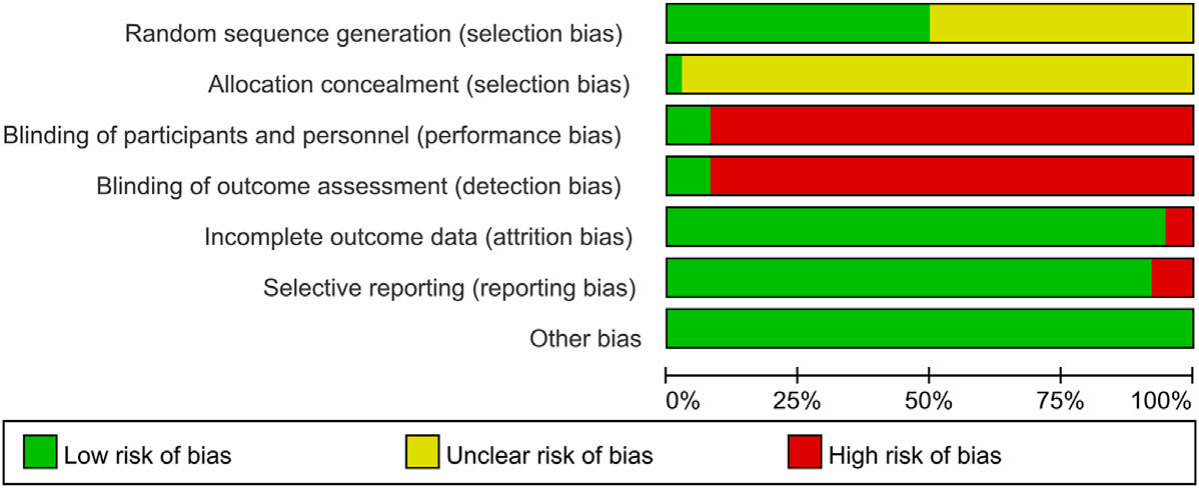
Risk of bias graph.

**FIGURE 3:**
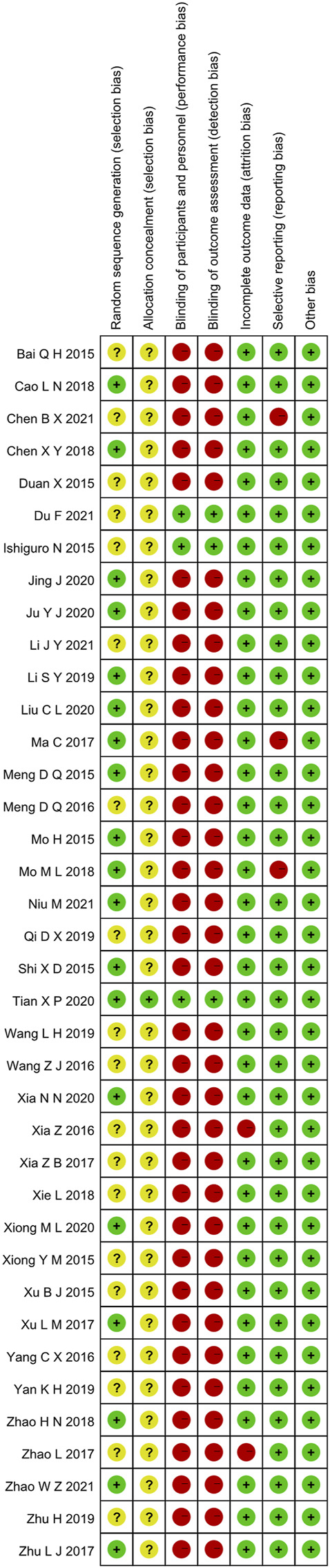
Risk of bias summary.

These RCTs were free of other sources of bias, so we assess them as low risk. The specific details (Figures 2, 3).

### 3.4 Primary Endpoints

#### 3.4.1 IGU Monotherapy

##### 3.4.1.1 ACR20

**FIGURE 4:**
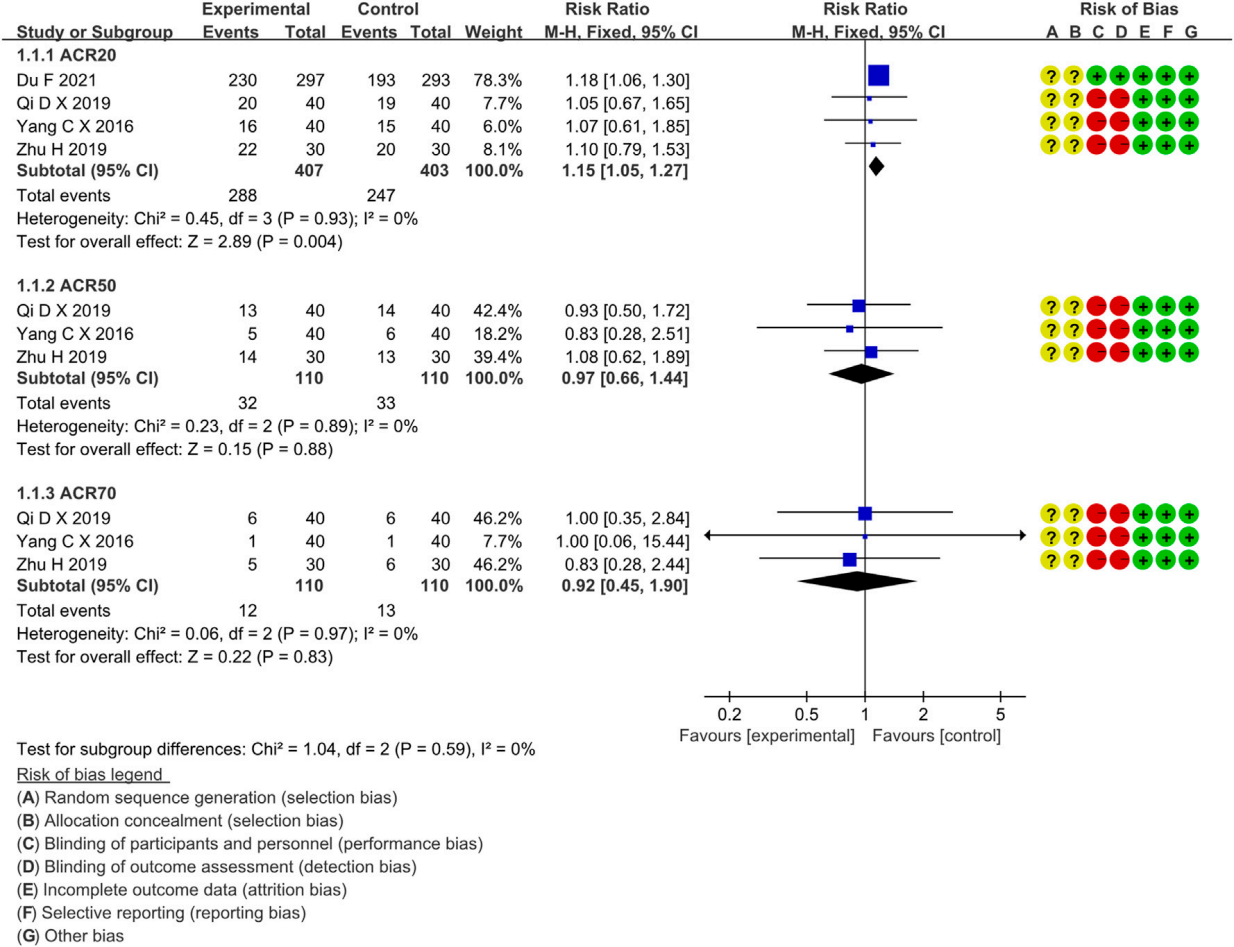
The forest plots of ACR20, ACR50, ACR70 improvement rate in the IGU vs. MTX group.

Four RCTs compared the ACR20 of the IGU and MTX groups, with 407 patients in the IGU alone group and 403 patients in the control group. There was a high degree of homogeneity (*p =* 0.93, I^2^ = 0%) among RCTs. It was decided to use the fixed-effect model. According to the data in Figure 4, the ACR20 of the IGU group was greater than that of the MTX group (RR 1.15, 95% CI 1.05–1.27, *p* = 0.004) among RA patients.

##### 3.4.1.2 ACR50

Four RCTs compared ACR50 between the IGU and MTX groups, with 110 patients in the IGU alone and 110 patients in the control group. There was a homogeneity (*p =* 0.89, I^2^ = 0%) among RCTs. It was decided to use the fixed-effect model. According to Figure 4, ACR50 between the IGU group and the MTX group is not statistically significant (RR 1.10, 95% CI 0.66–1.84, *p* = 0.72).

##### 3.4.1.3 ACR70

Three RCTs assessed ACR70 in RA patients, involving 110 patients in the IGU alone and 110 in the control group. There was a homogeneity (*p =* 0.97, I^2^ = 0%) among RCTs. It was decided to use the fixed-effect model. According to Figure 4, ACR70 of RA patients between the IGU and MTX groups has no significant difference (RR 0.92, 95% CI 0.45–1.90, *p* = 0.83).

##### 3.4.1.4 DAS28-ESR

Three RCTs assessed DAS28-ESR in RA patients, involving 91 patients in the IGU alone and 89 in the control group. There was low heterogeneity (*p =* 0.31, I^2^ = 4%, fixed-effects model) among RCTs. As shown in Figure 8, DAS28-ESR of the IGU alone group was lower than the MTX group (MD -0.15, 95% CI -0.27 to -0.03, *p* = 0.01).

#### 3.4.2 IGU + MTX

##### 3.4.2.1 ACR20

**FIGURE 5:**
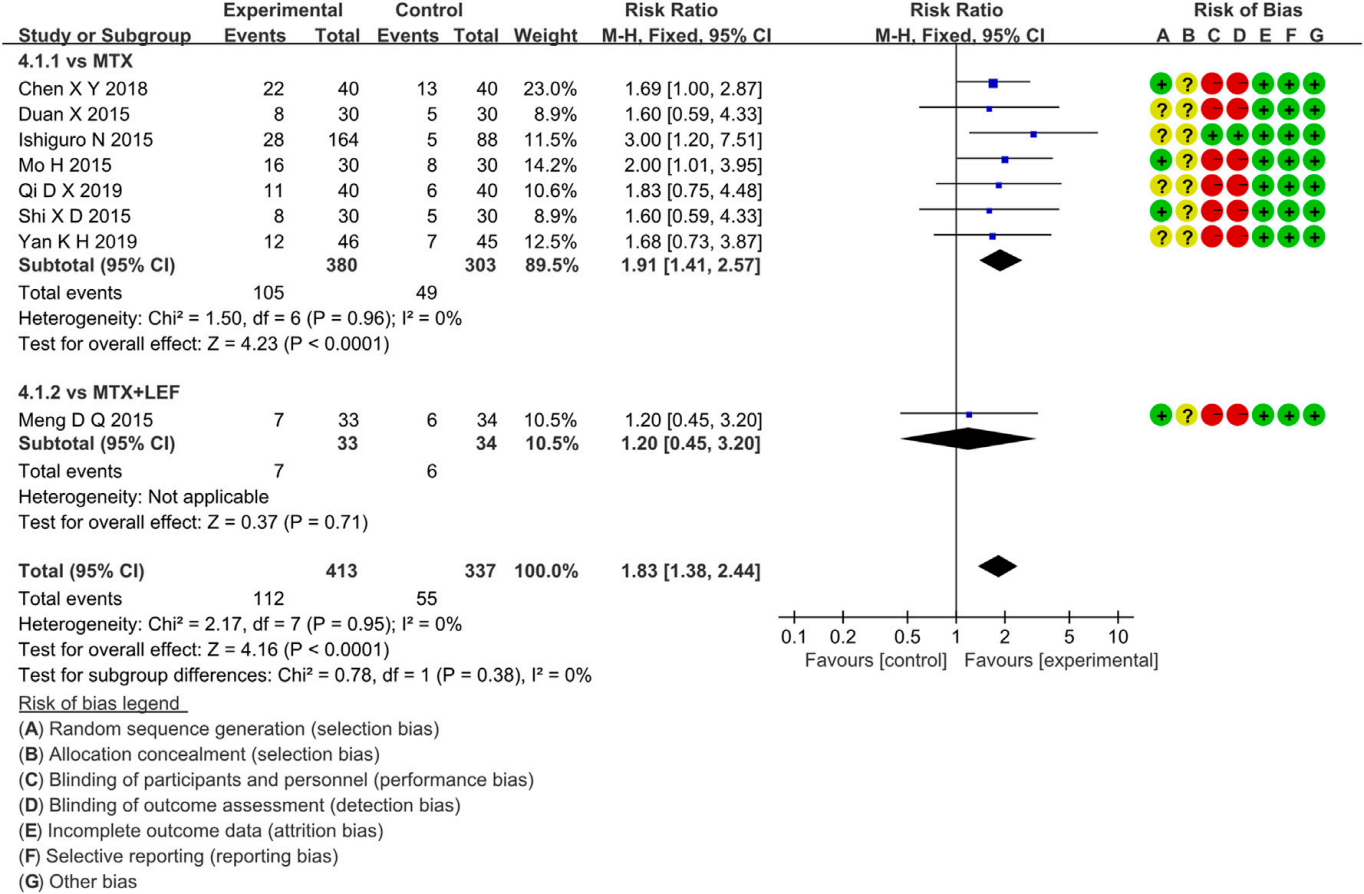
The forest plots of ACR20 improvement rate in the IGU + MTX vs. control groups.

Ten RCTs evaluated the ACR20 in RA patients, which involved 705 patients in the IGU + MTX group and 687 patients in the control group. According to the intervention characteristics of the control group, ten RCTs were divided into two subgroups (MTX alone subgroup and MTX + LEF subgroup). There was low heterogeneity in each subgroup (MTX subgroup: *p =* 0.14, I^2^ = 36%, MTX + LEF subgroup: *p =* 0.65, I^2^ = 0%). The fixed-effect model was used. As shown in Figure 5, there was a statistically significant difference (RR 1.24, 95% CI 1.14–1.35, *p* < 0.00001) in the MTX subgroup, and the IGU + MTX group indicated a higher incidence of ACR20 compared to the MTX group. However, between the IGU + MTX and the MTX + LEF group, ACR20 of RA patients showed no significant difference (RR 1.06, 95% CI 0.94–1.19, *p =* 0.38).

##### 3.4.2.2 ACR50

**FIGURE 6:**
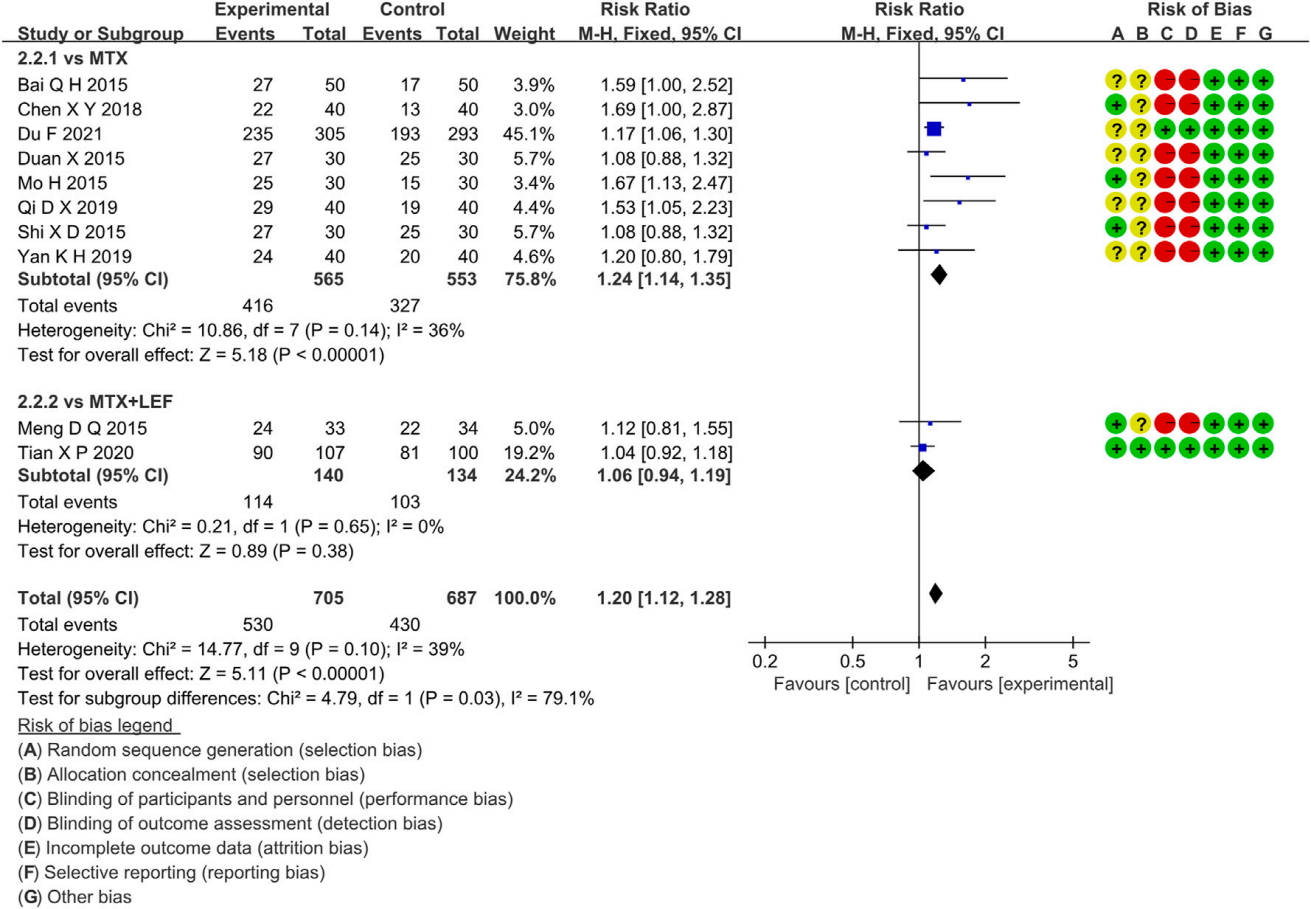
The forest plots of ACR50 improvement rate in the IGU + MTX vs. control groups.

Nine RCTs evaluated ACR50 in RA patients, which involved 457 patients in the IGU + MTX group and 382 patients in the control group. These studies were divided into two subgroups (MTX alone subgroup and MTX + LEF subgroup). There was homogeneity in each subgroup (MTX subgroup: *p =* 0.98, I^2^ = 0%, MTX + LEF subgroup: not applicable) among studies. The fixed-effect model was used. According to the data shown in Figure 6, there was a significant difference (RR 1.96, 95% CI 1.62–2.39, *p* < 0.00001) in the MTX subgroup, and the IGU + MTX group reflected a higher ACR50 compared to MTX. However, between the IGU + MTX and the MTX + LEF group, ACR50 of RA patients demonstrated no significant difference (RR 1.10, 95% CI 0.66–1.84, *p =* 0.72).

##### 3.4.2.3 ACR70

**FIGURE 7:**
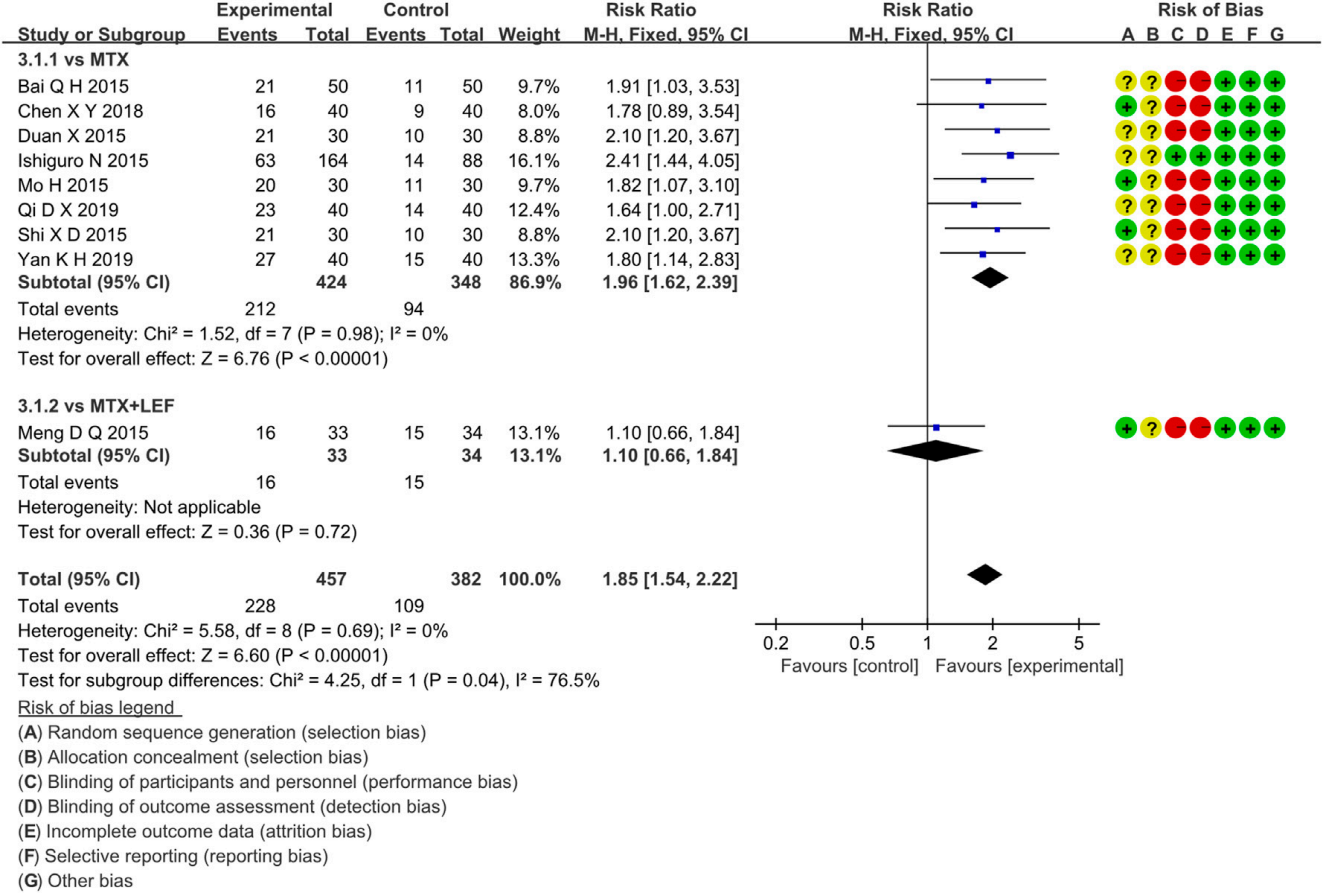
The forest plots of ACR70 improvement rate in the IGU + MTX vs. control groups.

Eight RCTs evaluated ACR70 in RA patients in the IGU + MTX group, involving 413 patients in the IGU + MTX group and 337 patients in the control group. These studies were divided into two subgroups (MTX alone subgroup and MTX + LEF subgroup). There was homogeneity between subgroups (MTX subgroup: *p =* 0.96, I^2^ = 0%, MTX + LEF subgroup: not applicable). The fixed-effect model was used. The data are presented in Figure 7. The MTX subgroup showed a significant difference (RR 1.91, 95% CI 1.41–2.57, p < 0.0001). This indicated that the incidence of ACR70 was higher in the IGU + MTX group than in MTX. However, the MTX + LEF subgroup showed no significant difference (RR 1.20, 95% CI 0.45–3.20, *p* = 0.71) among the groups, suggesting no difference in ACR70 between the IGU + MTX group and the MTX + LEF group.

##### 3.4.2.4 DAS28-ESR

**FIGURE 8:**
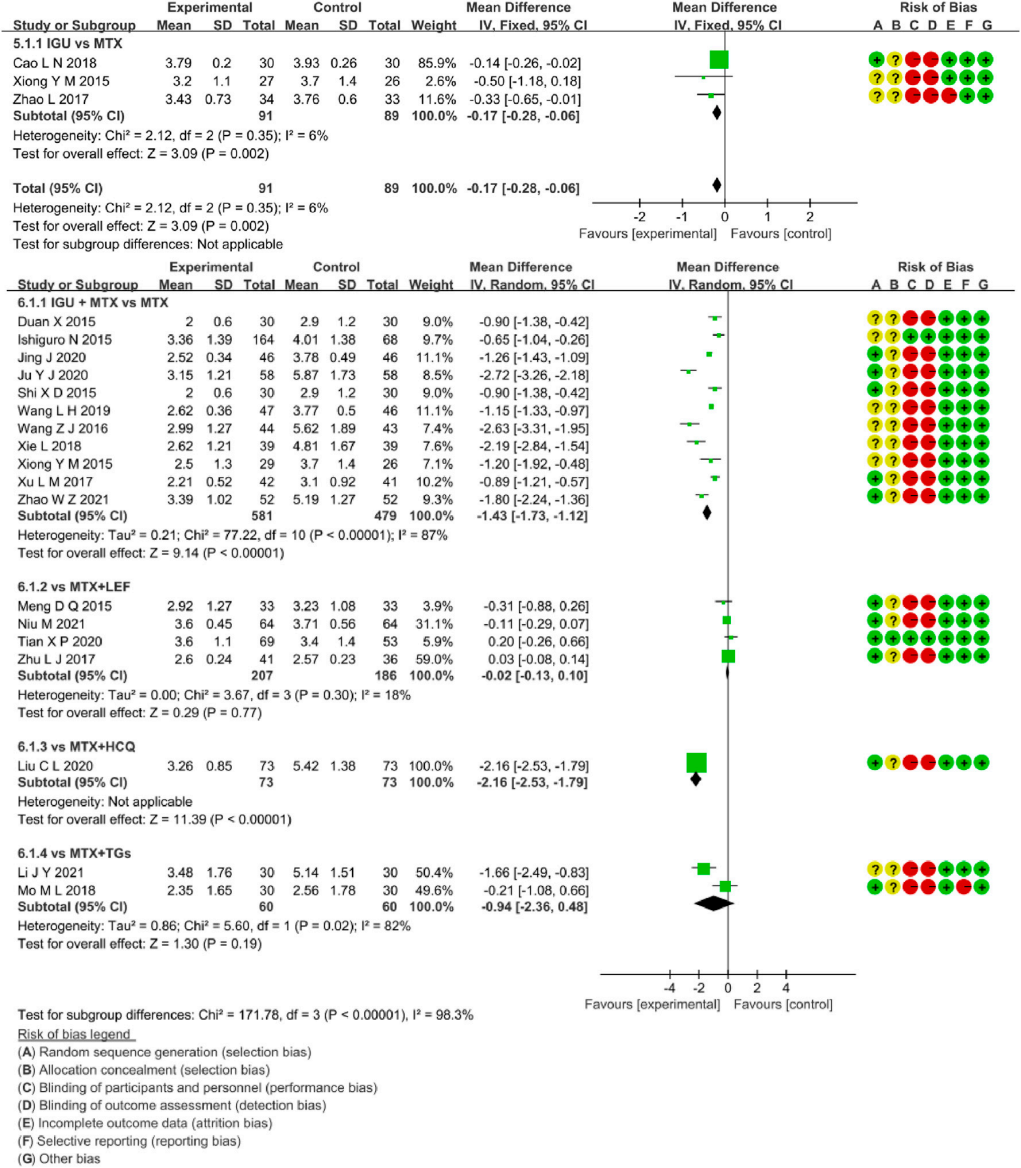
The forest plots of DAS28-ESR in the IGU monotherapy or IGU + MTX vs. control groups.

Eighteen RCTs evaluated DAS28-ESR in RA patients, involving 921 patients in the IGU + MTX group and 798 patients in the control group. These studies were divided into four subgroups (MTX monotherapy subgroup, MTX + LEF subgroup, MTX + HCQ, MTX + TGs subgroup) by the intervention characteristics of the control group. According to Figure 8, there was high heterogeneity between subgroups (MTX subgroup: *p* < 0.00001, I^2^ = 87%, MTX + LEF subgroup: *p =* 0.30, I^2^ = 18%, MTX + HCQ subgroup: not applicable, MTX + TGs subgroup: *p =* 0.02, I^2^ = 82%). For the random-effect model, the data showed a statistically significant difference in the MTX subgroup (MD −1.43, 95% CI −1.73 to −1.12, *p* < 0.00001) and MTX + HCQ subgroup (MD −2.16, 95% CI −2.53 to −1.79, *p* <0.00001), but no significant deference in the other two subgroups (MTX + LEF subgroup: MD −0.02, 95% CI −0.13 to −0.10, *p* = 0.77, MTX + TGs subgroup: MD -0.94, 95% CI −2.36 to 0.48, *p* = 0.19). Taken together, these RCTs reflected that DAS28-ESR of IGU + MTX was superior to MTX monotherapy and MTX + HCQ in RA patients. However, it was essentially the same as that of MTX + LEF and MTX + TGs.

### 3.5 Secondary Endpoints

The secondary endpoints contained the following: tender joint count-28 (TJC-28), swollen joint count-28 (SJC-28), morning stiffness (min), visual analog scale (VAS), patient global assessment (PGA), physician global assessment (EGA), Health Assessment Questionnaire (HAQ), erythrocyte sedimentation rate (ESR), C-reactive protein (CRP), anti-cyclic citrullinated peptides (anti-CCP), rheumatoid factors (RF). Results are shown in Table 2.

**TABLE 2:** Outcomes of secondary endpoints.

Outcomes	Types of Invention	Subgroup	Heterogeneity		Overall Effect				Statistical	Studies (N)	Participants(N)	Figures
			I^2^ (%)	*p*	MD	95%CI	*p*	Significant	Method			
TJC-28	IGU	MTX	70	0.04	-2.17	[-2.92, -1.42]	<0.00001	Yes	Random	3	237	S1
	IGU + MTX	MTX	0	0.44	-2.54	[-2.69, -2.38]	<0.00001	Yes	Random	5	427	S3
		MTX + LEF	32	0.22	-0.14	[-0.34,0.06]	0.16	No		4	413	
		MTX + HCQ	0		-0.76	[-0.94, -0.58]	<0.00001	Yes		1	146	
		MTX + TGs	37	0.21	-1.78	[-2.33, -1.23]	<0.00001	Yes		2	160	
		Summary	96	<0.00001	-1.40	[-1.98, -0.81]	<0.0001	Yes		12	1146	
SJC-28	IGU	MTX	90	<0.0001	-1.22	[-1.40, -1.04]	<0.00001	Yes	Random	3	237	S1
	IGU + MTX	MTX	5	0.38	-2.98	[-3.11, -2.85]	<0.00001	Yes	Random	5	427	S4
		MTX + LEF	73	0.01	-0.09	[-0.36, 0.18]	0.50	No		4	413	
		MTX + HCQ	0		-1.30	[-1.67, -0.93]	<0.00001	Yes		1	146	
		MTX + TGs	55	0.14	-1.99	[-2.66, -1.33]	<0.00001	Yes		2	160	
		Summary	99	<0.00001	-1.84	[-2.81, -0.87]	0.0002	Yes		12	1146	
VAS	IGU	MTX	0	0.45	-5.61	[-7.12, -4.11]	<0.00001	No	Fixed	3	234	S2
	IGU + MTX	MTX	0		-5.30	[-7.71, -2.89]	<0.0001	Yes	Random	1	60	S5
		MTX + TGs	0		-0.21	[-1.08, 0.06]	0.64	No		1	60	
		Summary	100	<0.00001	-2.63	[-7.61, 2.36]	0.30	No		2	180	
PGA	IGU	MTX	0	0.59	-2.73	[-3.52, -1.95]	<0.00001	Yes	Fixed	3	167	S2
	IGU + MTX	MTX	0	0.74	-12.77	[-13.40, -12.13]	<0.00001	Yes	Fixed	4	293	S5
EGA	IGU	MTX	0	0.85	-3.27	[-3.95, -2.59]	<0.00001	Yes	Fixed	3	234	S2
	IGU + MTX	MTX	73	0.01	-5.08	[-9.93, -0.82]	<0.00001	Yes	Random	4	275	S5
HAQ	IGU	MTX	0	0.89	0.00	[-0.01, 0.01]	0.96	No	Fixed	4	294	S2
	IGU + MTX	MTX	88	<0.00001	-0.17	[-0.50, 0.17]	0.34	No	Random	4	260	S5
		MTX + LEF	0		0.00	[-0.26, 0.26]	1.00	No		1	144	
		Summary	85	<0.0001	-0.13	[-0.41, 0.14]	0.34	No		5	404	
ESR	IGU	MTX	59	0.04	-6.34	[-6.89, -5.79]	<0.00001	Yes	Random	5	357	S1
										
	IGU + MTX	MTX	96	<0.00001	-15.27	[-20.31, -10.23]	<0.00001	Yes	Random	11	815	S6
		MTX + LEF	74	0.02	-2.37	[-9.64,4.90]	0.52	No		3	324	
		MTX + HCQ	0		-6.61	[-8.17, -5.05]	<0.00001	Yes		1	146	
		MTX + TGs	52	0.12	-5.91	[-8.86, -2.95]	<0.0001	Yes		3	220	
		Summary	97	<0.00001	-10.98	[-15.01, -6.96]	<0.00001	Yes		18	1505	
CRP	IGU	MTX	96	<0.00001	-5.91	[-9.45, -2.37]	0.001	Yes	Random	5	357	S1
	IGU + MTX	MTX	95	<0.00001	-10.87	[-14.31, -7.43]	<0.00001	Yes	Random	12	875	S7
		MTX + LEF	44	0.18	2.15	[-3.78,8.09]	0.48	No		2	199	
		MTX + HCQ	93	0.0001	-4.17	[-6.75, -1.58]	0.002	Yes		2	206	
		MTX + TGs	0	0.82	-2.56	[-4.14, -0.98]	0.001	Yes		2	120	
		Summary	97	<0.00001	-7.75	[-10.41, -5.08]	<0.00001	Yes		18	1400	
Anti-CCP	IGU	MTX	0		-13.00	[-18.40, -7.60]	<0.00001	Yes	Fixed	1	53	S2
	IGU + MTX	MTX	0	0.77	-17.51	[-22.66, -12.37]	<0.00001	Yes	Fixed	3	175	S8
		MTX + LEF	0		-125	[-277.88, 27.88]	0.11	No		1	142	
		MTX + TGs	0		-10.03	[-78.49, 58.43]	0.77	No		1	60	
		Summary	0	0.65	-17.59	[-22.72, -12.46]	<0.00001	Yes		5	377	
RF	IGU	MTX	0	0.35	-3.03	[-7.69,1.63]	0.20	No	Fixed	2	123	S2
	IGU + MTX	MTX	38	0.18	-32.11	[-34.54, -29.68]	<0.00001	Yes	Random	4	231	S9
		MTX + HCQ	0		-20.5	[-24.18, -16.82]	<0.00001	Yes		1	146	
		MTX + TGs	0		-354.4	[-435.91, -272.89]	<0.00001	Yes		1	60	
		Summary	95	<0.00001	-31.66	[-40.06, -23.26]	<0.00001	Yes		6	437	
Morning stiffness	IGU	MTX	98	<0.00001	-0.31	[-0.35, -0.28]	<0.00001	Yes	Random	2	174	S1
	IGU + MTX	MTX	99	<0.00001	-4.05	[-5.00, -3.09]	<0.00001	Yes	Random	8	686	S10
		MTX + LEF	41	0.19	-0.78	[-4.00,2.44]	0.63	No		2	205	
		MTX + TGs	0		-0.46	[-0.80, -0.12]	0.009	Yes		1	60	
	Summary		98	<0.00001	-1.21	[-1.70, -0.73]	<0.00001	Yes		10	951	

#### 3.5.1 Adverse Events

##### 3.5.1.1 IGU Monotherapy

**FIGURE 9:**
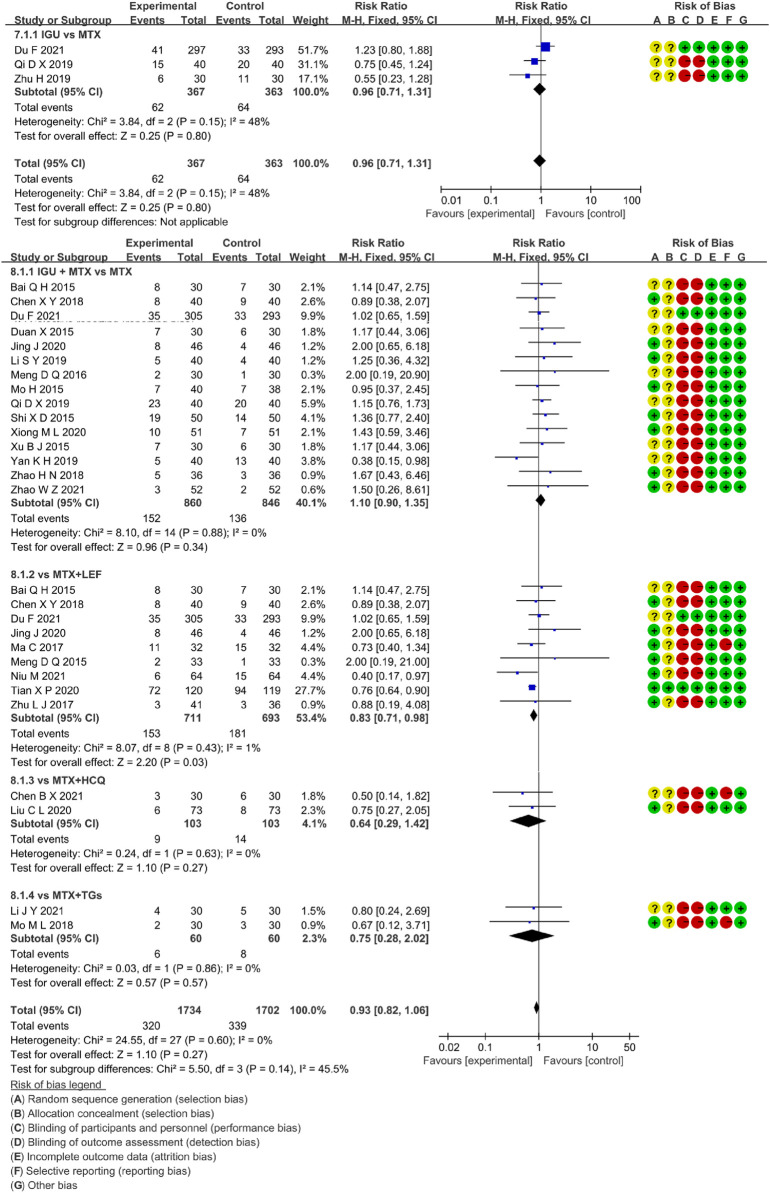
The forest plots of AEs rate in the IGU monotherapy or IGU + MTX vs. control groups.

Three RCTs assessed the incidence rate of AEs in RA patients, involving 367 patients in the IGU alone and 363 patients in the control group. Figure 9 showed that there was low heterogeneity (*p =* 0.15, I^2^ = 48%) and no significant difference among trials (RR 0.96, 95% CI 0.71–1.31], *p =* 0.80, fixed-effect model). These findings suggested that the AEs of IGU monotherapy was as comparable to MTX.

##### 3.5.1.2 IGU + MTX

Twenty-four RCTs evaluated the incidence rate of adverse events in RA patients, involving 1734 patients in the IGU + MTX group and 1702 patients in the control group. The intervention features of the control group split these investigations into four subgroups (MTX monotherapy subgroup, MTX + LEF subgroup, MTX + HCQ subgroup, MTX + TGs subgroup). Each subgroup had a high degree of homogeneity (MTX subgroup: *p =* 0.88, I^2^ = 0%, MTX + LEF subgroup: *p =* 0.43, I^2^ = 1%, MTX + HCQ subgroup: *p =* 0.63, I^2^ = 0%, MTX + TGs subgroup: *p =* 0.86, I^2^ = 0%). It was decided to employ the fixed-effect model. Figure 9 demonstrated a statistically significant in the MTX + LEF subgroup (RR 0.83, 95% CI 0.71–0.98, *p =* 0.03) and no significant difference in the other three subgroups (MTX subgroup, RR 1.10, 95% CI 0.90–1.35, *p =* 0.34), MTX + HCQ subgroup, RR 0.64, 95% CI 0.29–1.42, *p =* 0.27), MTX + TGs subgroup, RR 0.75, 95% CI 0.28–2.02, *p =* 0.57). In these RCTs, the AEs of IGU + MTX group was found to be as comparable as that of MTX monotherapy, MTX + HCQ, and MTX + TGs, however, it was lower than that of MTX + LEF.

### 3.6 Other Subgroup Analysis

We performed subgroup analyses of primary endpoints and safety based on the course of therapy, stage of disease, and age of RA patients. We also investigated adverse event data based on common side effects of IGU, such as leukopenia and elevated LFTs. According to the results shown in Table 3, we concluded that when the intervention (IGU alone or IGU + MTX) lasted for 52 weeks, it demonstrated superior efficacy in improving the ACR20 of patients without prominent adverse events. Notably, IGU or IGU + MTX had apparent advantages in improving the ACR20 of first-visit RA. IGU + MTX had apparent benefits in improving DAS28-ESR of refractory RA. Regarding adverse events, IGU or IGU + MTX did not raise leukopenia risk while decreasing LFTs' risk. It was equally as safe for young/middle-aged and elderly populations as the control group. The same is true for refractory and first-visit RA.

**TABLE 3:** Other subgroup analysis.

Outcomes	Types of Invention		Subgroup	Heterogeneity		Overall Effect				Statistical method	Studies(N)	Participants (N)	Figures
				I^2^ (%)	*p*	MD	95%CI	*p*	Significance				
DAS28-ESR-course	IGU		12w	0	0.90	-0.14	[-0.25, -0.02]	0.02	Yes		2	113	
			24w	0	0.66	-0.36	[-0.65, -0.07]	0.01	Yes		2	120	
			Summary	0	0.54	-0.36	[-0.65, -0.07]	0.002	Yes	Fixed	3	233	S13
	IGU + MTX		12w	70	0.04	-1.04	[-1.63, -0.44]	0.0007	Yes		3	175	
			24w	91	<0.00001	-1.31	[-1.84, -0.79]	<0.00001	Yes		6	703	
			52w	0		-0.89	[-1.21, -0.57]	<0.00001	Yes		1	83	
			Summary	87	<0.00001	-1.18	[-1.48, -0.89]	<0.00001	Yes	Random	10	961	S14
ACR20-course	IGU		52w	0		1.18	[1.06, 1.30]	0.002	Yes		1	590	S15
	IGU + MTX		12w	0	0.40	1.48	[1.16, 1.91]	0.002	Yes		3	240	
			24w	48	0.11	1.23	[1.01, 1.49]	0.04	Yes		5	362	
			52w	0		1.17	[1.06, 1.30]	0.003	Yes		1	598	
			Summary	30	0.17	1.23	[1.10, 1.38]	0.0003	Yes	Random	8	1200	S16
ACR50-course	IGU + MTX		12w	0	0.86	1.91	[1.38, 2.64]	<0.0001	Yes		3	240	
			24w	0	0.88	2.06	[1.60, 2.64]	<0.00001	Yes		5	552	
			Summary	0	0.98	2.01	[1.64, 2.45]	<0.00001	Yes	Fixed	7	792	S17
ACR70-course	IGU + MTX		12w	0	0.92	2.06	[1.30, 3.24]	0.002	Yes		2	140	
			24w	0	0.75	2.07	[1.28, 3.33]	0.003	Yes		6	452	
			Summary	0	0.94	2.06	[1.48, 2.88]	<0.0001	Yes	Random	6	592	S18
AEs-course	IGU		24w	0	0.53	0.68	[0.44, 1.05]	0.08	No		2	140	
			52w	0		1.26	[0.77,2.06]	0.35	No		1	590	
			Summary	48	0.15	0.96	[0.71, 1.31]	0.80	No	Fixed	3	730	S19
	IGU + MTX		12w	15	0.32	0.72	[0.42,1.24]	0.23	No		4	290	
			24w	0	0.58	0.95	[0.76, 1.19]	0.66	No		12	1041	
			52w	20	0.28	0.84	[0.71, 1.01]	0.06	No		3	1041	
			Summary	0	0.47	0.87	[0.76, 1.00]	0.06	No	Fixed	19	2228	S20
DAS28-ESR-stage	IGU		Refractory	0		-1.66	[-2.49, -0.83]	<0.0001	Yes	Fixed	1	60	S21
	IGU + MTX		Refractory	30	0.22	-2.42	[-2.79, -2.06]	<0.00001	Yes		4	401	
			First-visit	0		-0.90	[-1.38, -0.42]	0.0002	Yes		1	60	
			Summary	85	<0.00001	-2.12	[-2.81, -1.44]	<0.00001	Yes	Random	5	461	S22
ACR20-stage	IGU		First-visit	0		1.18	[1.06, 1.30]	0.002	Yes	Fixed	1	590	S21
	IGU + MTX		First-visit	0	0.47	1.16	[1.06, 1.27]	0.002	Yes	Fixed	2	658	S22
ACR50-stage	IGU + MTX		First-visit	0		2.1	[1.20, 3.67]	0.009	Yes	Fixed	1	60	S22
ACR70-stage	IGU + MTX		First-visit	0		1.6	[0.59, 4.33]	0.36	No	Fixed	1	60	S22
AEs-stage	IGU		First-visit	0		1.26	[0.77,2.06]	0.35	No	Fixed	1	590	S21
	IGU + MTX		Refractory	0	0.86	1.11	[0.60, 2.05]	0.74	No		5	312	
			First-visit	0	0.7	1.1	[0.78, 1.56]	0.58	No		3	775	
			Summary	0	0.96	1.1	[0.82, 1.49]	0.51	No	Fixed	8	1087	S22
DAS28-ESR-age	IGU		Young/middle aged	0		-0.44	[-0.79,-0.09]	0.01	Yes		1	61	
			Elderly	0		-0.14	[-0.26,-0.02]	0.02	Yes		1	60	
			Summary	60	0.11	-0.24	[-0.52, 0.04]	0.09	No	Random	2	121	S23
	IGU + MTX		Young/middle aged	73	0.05	-2.18	[-3.06,-1.31]	<0.00001	Yes		2	164	
			Elderly	0		-1.35	[-1.47,-1.23]	<0.00001	Yes		1	60	
			Summary	86	<0.00001	-1.84	[-2.47, -1.21]	<0.00001	No	Random	3	224	S25
ACR20-age	IGU		Young/middle aged	0	0.32	1.05	[0.79, 1.53]	0.57	No		1	80	
			Elderly	0		1.1	[0.79, 1.53]	0.57	No		1	60	
			Summary	0	0.67	1.08	[0.82, 1.42]	0.24	No	Random	2	140	S23
	IGU + MTX		Young/middle aged	0	0.93	1.52	[1.15, 2.00]	0.003	Yes	Fixed	3	241	S25
ACR50-age	IGU		Young/middle aged	0	0.57	0.93	[0.50, 1.72]	0.84	No		1	80	
			Elderly	0		1.08	[0.62, 1.89]	0.81	No		1	60	
			Summary	0	0.85	1.00	[0.66, 1.52]	1.00	No	Fixed	2	140	S23
	IGU + MTX		Young/middle aged	23	0.27	1.51	[1.07, 2.12]	0.02	Yes	Fixed	3	241	S25
ACR70-age	IGU		Young/middle aged	0		1.00	[0.35,2.84]	0.74	No		1	80	
			Elderly	0		0.83	[0.28,2.44]	1.00	No		1	60	
			Summary	0	0.97	0.92	[0.43,1.94]	0.82	No	Fixed	2	140	S24
	IGU + MTX		Young/middle aged	0		1.83	[0.75, 4.48]	0.18	No	Fixed	1	80	S25
AEs-age	IGU		Young/middle aged	0		0.75	[0.75,1.24]	0.27	No		1	80	
			Elderly	0		0.55	[0.23,1.28]	0.17	No		1	60	
			Summary	0	0.76	0.68	[0.43, 1.03]	0.07	No	Fixed	2	140	S24
	IGU + MTX		Young/middle aged	0	0.64	0.95	[0.71,1.28]	0.78	No	Fixed	10	887	S25
AEs	IGU + MTX		leukopenia	0	0.73	0.63	[0.37, 1.07]	0.09	No	Fixed	10	1038	S26
			LFTs	0	0.61	0.56	[0.40, 0.77]	0.0004	Yes	Fixed	15	1365	S26

### 3.7 Sensitive Analysis

We changed the effect model to evaluate the sensitivity of this meta-analysis and observed the changes in RR (OR) and MD (SMD) after changing the effect model. The results showed that the MD of morning stiffness, SJC-28, ESR, and CRP in the IGU VS. MTX group and the MD of TJC-28, SJC-28, morning stiffness, VAS, EGA, HAQ, RF, anti-CCP, ESR, and CRP in the IGU + MTX VS. MTX group changed significantly, and the results may have some risks. RR (OR) and MD (SMD) of other indicators did not change much, which could be considered robust results. The comparison results are shown in Table 4.

**TABLE 4:** Sensitive analysis of all the outcomes and DAS28-ESR of IGU + MTX.

All the Outcomes				
Outcomes	IGU monotherapy		IGU + MTX	
	Fixed-effect model	Random-effect mode	Fixed-effect model	Random-effect mode
ACR20	1.15 [1.05, 1.27]	1.16 [1.06, 1.28]	1.20 [1.12, 1.29]	1.18 [1.08, 1.29]
ACR50	0.97 [0.66, 1.44]	0.98 [0.67, 1.45]	1.85 [1.54, 2.22]	1.80 [1.51, 2.16]
ACR70	0.92 [0.45, 1.90]	0.92 [0.45, 1.90]	1.83 [1.38, 2.44]	1.78 [1.35, 2.36]
DAS28-ESR	-0.15 [-0.27, -0.03]	-0.16 [-0.31, -0.00]	-0.80 [-0.83, -0.77]	-0.76 [-1.08, -0.44]
TJC-28	-2.15 [-2.45, -1.86]	-2.17 [-2.92, -1.42]	-0.99 [-1.07, -0.91]	-1.55 [-2.26, -0.84]
SJC-28	-1.22 [-1.40, -1.04]	-0.37 [-1.94, 1.20]	-0.01 [-0.11, 0.08]	-0.09 [-0.36, 0.18]
Morning stiffness	-0.31 [-0.35, -0.28]	-3.02 [-8.45, 2.40]	-0.63 [-0.66, -0.60]	-2.49 [-3.09, -1.88]
VAS	-5.61 [-7.12, -4.11]	-5.61 [-7.12, -4.11]	-0.61 [-0.68, -0.55]	-1.12 [-1.51, -0.74]
PGA	-2.73 [-3.52, -1.94]	-2.73 [-3.52, -1.95]	-12.77 [-13.40, -12.13]	-12.77 [-13.40, -12.13]
EGA	-3.27 [-3.95, -2.59]	-3.27 [-3.95, -2.59]	-3.34 [-3.99, -2.69]	-5.08 [-9.33, -0.82]
HAQ	-0.00 [-0.01, 0.01]	-0.00 [-0.01, 0.01]	-0.16 [-0.27, -0.06]	-0.13 [-0.41, 0.14]
RF	-3.03 [-7.69, 1.63]	-3.03 [-7.69, 1.63]	-31.47 [-32.90, -30.04]	-34.97 [-47.83, -22.11]
Anti-CCP	-13.00 [-18.40, -7.60]	-13.00 [-18.40, -7.60]	-11.38 [-12.07, -10.68]	-11.57 [-15.69, -7.45]
ESR	-6.34 [-6.89, -5.79]	-4.77 [-7.71, -1.83]	-11.29 [-11.98, -10.59]	-10.98 [-15.01, -6.96]
CRP	-5.26 [-5.72, -4.79]	-5.91 [-9.45, -2.37]	-7.01 [-7.41, -6.60]	-7.75 [-10.41, -5.08]
AEs	0.96 [0.71, 1.31]	0.87 [0.55, 1.36]	0.93 [0.82, 1.06]	0.89 [0.79, 1.00]

**DAS28-ESR of IGU + MTX**					
Study	Heterogeneity		Study	Heterogeneity	
	I (%)	*p*		I (%)	*p*
Duan X 2015	88	<0.00001	Wang Z J 2016	88	<0.00001
Ishiguro N 2015	87	<0.00001	Xie L 2018	87	<0.00001
Jing J 2020	88	<0.00001	Xiong Y M 2015	88	<0.00001
Ju Y J 2020	81	<0.00001	Xu L M 2017	88	<0.00001
Shi X D 2015	88	<0.00001	Zhao W Z 2021	87	<0.00001
Wang L H 2019	88	<0.00001			

The IGU + MTX DAS28-ESR analysis had a higher heterogeneity. We did a sensitivity analysis to determine which study was driving this heterogeneity. However, we observed that regardless of which study was removed, there was still a high degree of heterogeneity. The results are shown in Table 4.

### 3.8 Publication Bias Analysis

Egger’s and Harbord’s texts shown in Table 5. 1) IGU alone: ACR20: there may be a publication bias (*p* = 0.097); ACR50: the possibility of publication bias was small (*p* = 0.752); ACR70: the possibility of publication bias was small (*p* = 0.876); DAS28-ESR: the possibility of publication bias was small (*p* = 0.684); adverse events: there may be a publication bias (*p* = 0.046). 2) IGU + MTX: ACR20: the possibility of publication bias was small (*p* = 0.419); ACR50: the possibility of publication bias was small (*p* = 0.990); ACR70: there may be a publication bias (*p* = 0.032); DAS28-ESR: the possibility of publication bias was small (*p* = 0.168); adverse events: the possibility of publication bias was small (*p* = 0.196).

**TABLE 5:** Publication bias texts.

	Egger’s Tests (P)	Harbord’s Texts (P)			
	DAS28-ESR	ACR20	ACR50	ACR70	AEs
IGU monotherapy	0.684	0.097	0.752	0.876	0.046
IGU + MTX	0.168	0.419	0.990	0.032	0.196

### 3.9 Evidence Quality Assessment

**FIGURE 10:**
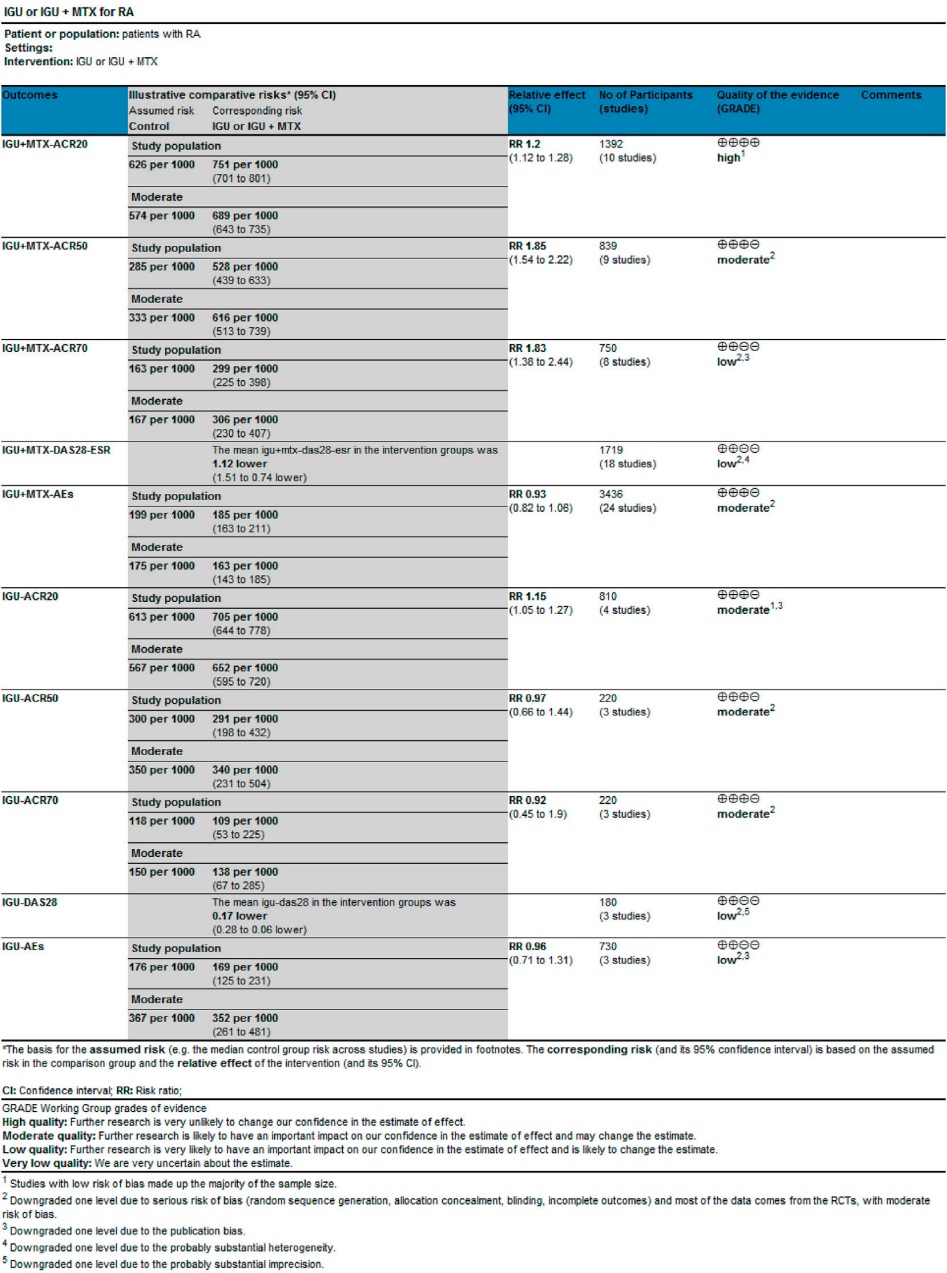
The quality assessment of ACR20, ACR50, ACR70, DAS28-ESR, and AEs.

We evaluated the quality of evidence for the primary outcomes using GRADEprofile. The results are as follows: 1) IGU alone: The quality of ACR20, ACR50, and ACR70 was moderate; the quality of AEs and DAS28-ESR was low. 2) IGU + MTX: The quality of ACR20 was high. The quality of ACR50and AEs were moderate; the quality of ACR70 and DAS28-ESR was low (Figure 10).

## 4 Discussion

### 4.1 Primary Outcomes Summary

This systematic review and meta-analysis included 38 RCTs involving 4302 participants. The primary outcomes were as follows: 1) IGU vs. MTX: IGU only was more effective in improving the ACR20 and DAS28-ESR. The symptom assessment indicators (TJC-28, VAS, PGA, EGA) were lower, but the indicators (SJC-28, HAQ) were not statistically significant across groups. The inflammatory immune assessment indicators (ESR, CRP, anti-CCP) were lower. However, the markers (RF) were not substantially different. In addition, the AEs between the IGU and MTX groups showed no significant variations. 2) IGU + MTX vs. MTX: The IGU + MTX group improved the ACR20, ACR50, and ACR70 rate and DAS28-ESR score more effectively. The symptom assessment indicators (Morning stiffness time, TJC-28, SJC-28, VAS, PGA, EGA, HAQ) and the inflammatory immune assessment indicators (ESR, CRP, RF, anti-CCP) were lower. The AEs among groups were no significant variations. 3) IGU + MTX vs. MTX + LEF: There was no significant difference between groups in ACR20, ACR50, ACR70, or DAS28-ESR levels. The symptom assessment indicators (Morning stiffness, SJC-28, TJC-28, and HAQ) were not statistically significant. The inflammatory immune evaluation signs (ESR, CRP, RF, anti-CCP) were not statistically significant. The IGU + MTX group had lower AEs. 4) IGU + MTX vs. MTX + HCQ: The IGU + MTX group had lower symptom-related indicators (TJC and SJC) than the MTX + HCQ group. Indicators of inflammation and immunity (ESR, CRP, and RF) were also lower. Furthermore, there was no discernible change in AEs. 5) IGU + MTX vs. MTX + tripterygium glycosides: DAS28-ESR was not statistically significant among the group. The symptom assessment indicators (Morning stiffness, TJC, and SJC) and the inflammatory immune assessment indicators (ESR, CRP, RF) were lower, but the anti-CCP showed no discernible change. The AEs among groups were no significant variations. 6) Subgroup analysis results: When the intervention lasts for 52 weeks, IGU alone or IGU + MTX had greater ACR20 of patients without prominent adverse events. IGU or IGU + MTX was more effective in improving the ACR20 of first-visit RA. IGU + MTX was more effective in improving DAS28-ESR of refractory RA. Regarding adverse events, IGU or IGU + MTX did not raise the risk of leukopenia while decreasing the risk of LFTs. It was equally as safe for young/middle-aged and elderly populations as the control group. The same is true for refractory and first-visit RA. 7) Sensitivity analysis showed that the primary outcome indicators were consistent with the actual analysis results, suggesting that IGU alone or IGU + MTX could effectively improve the clinical efficacy of RA and was superior to the control group. 8) Publication bias for the primary endpoint showed the possibility of publication bias for ACR50, ACR70, and DAS28-ESR in the IGU group, and ACR20, ACR50, DAS28-ESR, and AEs in the IGU + MTX group was small. There may be a publication bias for ACR20, AEs in the IGU group, and ACR70 in the IGU + MTX group.

### 4.2 Evidence of Applicability

Rheumatoid arthritis is an autoimmune disease characterized by chronic erosive arthritis. It alternates between progressive and stable phases. The pathogenesis is complex, treatment difficult, and the cure rate low (Mu et al., 2021). Current treatment goals are mainly to control symptoms, delay disease progression, and improve quality of life (Hunter et al., 2017).

MTX is the first-line clinical drug recommended by the EULAR and has been proven to have an excellent anti-inflammatory effect. Its primary mechanism of action targets and affects the TNF-α pathway in inflammatory disease (Lu et al., 2009; Mimori et al., 2019). However, in some cases, MTX or If the patient does not improve after 3 months or the treatment target is not met after 6 months, another DMARD, such as MTX + LEF, MTX + HCQ, MTX + adalimumab, or MTX + tripterygium glycosides, should be used. However, long-time use of them was often associated with various problems, which limited their clinical application to some extent.32,33 (Jiang et al., 2020; Xie et al., 2020).

IGU is a new small molecule drug with effectiveness as well as safety. It possesses anti-inflammatory, immune-regulatory, and bone-protective properties (Jiang et al., 2020; Xie et al., 2020). A twice-daily therapeutic dosage of 25 mg has been demonstrated to be efficacious and well-tolerated, and it has nothing to do with food (Xiao et al., 2018). Previous studies have shown that IGU can reduce prostaglandin production in inflammatory tissues by COX-2 inhibition; Inhibit the bradykinin release from inflammatory tissues; Inhibit Il-1 *β* and IL-6 release from monocytes: inhibition of antigen-specific T-cell proliferation; Reduce IgG and IgM levels produced by B cells in RA patients; Stimulate osteoblast differentiation and bone construction; Inhibit costimulatory factor and cytokine production, expression in synovial cells (Garcia, 2019; Jiang et al., 2020; Xie et al., 2020). Wang X et al. found that the combination of IGU and MTX significantly inhibited the high expression of RANKL mRNA (compared with MTX alone, *p* < 0.01; compared with IGU, *p* < 0.05) (Wang et al., 2017). Clinical studies have shown that IGU could coordinate with MTX to reduce inflammation in RA patients, promote bone formation, and antagonize bone absorption (Yan et al., 2018).

### 4.3 Sources of Heterogeneity

Sources of heterogeneity in this study: 1) Only 22 RCTs referred to the specific stochastic method, and 16 RCTs did not describe the random sequence generation. The allocation concealment was mentioned in only one RCT. A double-blind technique was used in two RCTs. Allocation concealment and blinding were not mentioned in the other RCTs. The results of two RCTs are missing. There was selective reporting in three RCTs.These are sources of publication bias. 2) The sensitivity analysis revealed that the MD of morning stiffness, SJC-28, ESR, and CRP in the IGU VS. MTX group and the MD of TJC-28, SJC-28, morning stiffness, VAS, EGA, HAQ, RF, Anti-CCP, ESR, and CRP in the IGU + MTX VS. MTX group had changed significantly. There may be certain risks. 3) Samples were dropped in four studies, which may introduce some bias. 4) While DAS28-ESR heterogeneity was substantial in the IGU + MTX group, we performed a series of subgroup and sensitivity analyses. However, it remained very varied, which might be attributed to varying follow-up intervals, illness progression, or other factors. 5) The symptom assessment indicators (morning stiffness time, TJC, SJC, VAS, PGA, EGA, HAQ) were subjective, and implementation bias and measurement bias may occur in evaluating results. 6) There were few RCTs with extractable data in subgroups such as MTX + LEF, MTX + TGs, and MTX + HCQ, and the conclusions were unstable. More relevant RCTs are needed to modify or verify the results.

### 4.4 Safety of IGU or IGU + MTX

Safety analysis showed no significant difference in the incidence of AEs between the groups of IGU + MTX vs. MTX alone, IGU + MTX vs. MTX + TGs, and IGU vs. MTX. However, the incidence of AEs in the IGU + MTX group was lower than in the MTX + LEF group. IGU + MTX does not increase the risk of leukopenia, but it can decrease the risk of LFTs. Recently, a multicenter, randomized, double-blind, parallel controlled trial of rheumatoid arthritis showed no significant difference in the incidence of adverse events after 52 weeks of treatment with IGU alone or in combination with MTX compared to MTX (Du et al., 2021). Another study showed that IGU combined with MTX is safer than LEF combined with MTX (Tian et al., 2020). These studies showed that IGU was safe for long-term use compared to other DMARDs. A 52-week, multicenter, prospective, observational, phase IV IGU clinical trial in Japan found that the incidence of AEs peaked after approximately 4 weeks of treatment. Subsequently, the incidence of various AEs did not increase over time (Mimori et al., 2019). Long-term use of IGU was safe, with relatively few adverse reactions problems (vomiting, abdominal pain, diarrhea, loss of appetite, etc.) and liver malfunction (elevated transaminases) were the most common side effects, followed by leukopenia, skin rash, and itching (Tian et al., 2020). Furthermore, a multicenter, prospective, real-world phase IV clinical study from China reflected the better safety profile of IGU. It showed that IGU as a combination did not increase the risk of liver damage. In contrast, the combination of IGU and LEF increased the risk of leukopenia and IGU-related kidney disease by < 1%. While phase IV study in Japanese, less than 5.1% (136/2666) could be related to differences in patient age and disease course (Mimori et al., 2019). In addition, the study found no significant increase in AEs in elderly patients with active RA compared to adults under 65 years of age (Mu et al., 2021). The treatment of RA with interstitial lung disease (ILD) was a clinical contradiction because MTX, LEF, and bDMARD were all associated with RA-ILD (Li et al., 2013; Ishikawa and Ishikawa, 2019; Mimori et al., 2019). However, retrospective observational studies have shown that IGU combined with LEF, HCQ, sulfadiazine, and other DMARDs was safe in treating chronic interstitial pneumonia complicated with RA (Mimori et al., 2017). More studies are needed to explore the correlation between RA and ILD adverse reactions. Other adverse effects of IGU include oral ulcers, dizziness, and headache (Mo and Ma, 2015; Shi et al., 2015; Jing et al., 2020). We still need more sample size and more time to verify the safety of the IGU.

### 4.5 Strengths and Limitations of this Study

This study is the latest systematic review and meta-analysis of the efficacy and safety of IGU monotherapy or combined with MTX, providing an evidence-primarily based foundation and new directions for clinical management, as well as new research directions for future RCTs. We conducted subgroup analyses of IGU monotherapy or IGU + MTX based on intervention in the control group, treatment duration, illness stage, and patient age, as well as an investigation of adverse event data based on common IGU side effects. We conducted evidence quality assessment, sensitivity analysis, and publication bias analysis to verify the reliability and recommendation of the outcomes.

The limitations of this study are the high or insignificant risks of random sequence generation, blinding, allocation concealment, incomplete data, and selective reporting for most RCTs. These directly affect the accuracy of the results and the level of evidence. The heterogeneity of some outcome indicators is high, which may be due to different patient baseline data, drug doses, and background treatments in different studies. In addition, randomized controlled trials of some subgroup analyses were rare. It is necessary to develop more RCTs from different regions and ethnic groups with straightforward random sequence generation methods, allocation concealment, and blinding, based on the patients' age, disease stage, and course, to modify or validate the results. Furthermore, current IGU RCTs mainly focus on China and Japan, and evidence may be lacking in other countries, making the evidence extrapolable primarily to Asia.

### 4.6 Reflections on Future Research

In future clinical practice, it is necessary to conduct more RCTs of IGU coupled with additional csDMARDs to broaden the therapeutic possibilities. IGU combined with csDMARD demonstrated a curative effect. When used with IGU, Wu et al. discovered that leflunomide reduced DAS28, joint symptom-related indicators, and inflammatory immunological indicators (Wu et al., 2021). IGU can also be used with biologic disease-modifying anti-rheumatic medications (bDMARDs) to treat individuals who do not react well to biological medicines (Yoshikawa et al., 2018). Combining etanercept with IGU, for example, may increase ACR20, ACR50, and ACR70 while lowering common symptom-related and inflammatory immunological indicators in people who have low etanercept effectiveness (Sun et al., 2016). In addition, the combination of IGU dramatically decreased disease activity in individuals with a poor response to tocilizumab (DAS28, CDAI, and EULAR response criteria) (Ebina et al., 2019).

## 5 Conclusion

1) When compared to the MTX alone subgroup, IGU alone offers clear advantages in improving ACR20 and DAS28-ESR, despite the low quality of evidence for DAS28-ESR findings. Compared to standard therapies, IGU + MTX shows clear benefits in improving ACR20, ACR50, ACR70, and DAS28-ESR scores. However, the quality of evidence for ACR70 and DAS28-ESR findings is much lower than that of ACR20. 2) Regarding adverse reactions, IGU or IGU + MTX does not increase the incidence of AEs. IGU + MTX is safer than MTX + LEF. In the future, IGU or IGU + MTX may be utilized as an alternate therapy for some RA patients with poor effectiveness or tolerance to MTX, tripterygium, or leflunomide. 3) In terms of subgroup analysis, when the intervention (IGU alone or IGU + MTX) lasts for 52 weeks, it demonstrated superior efficacy in improving the ACR20 of patients without obvious adverse events. In addition, IGU or IGU + MTX has obvious advantages in improving the ACR20 of first-visit RA. IGU + MTX has obvious advantages in improving DAS28-ESR of refractory RA. For adverse events analysis, IGU + MTX does not increase the risk of leukopenia but can decrease LFTs' risk. IGU or IGU + MTX is just as safe as the control group for young/middle-aged, elderly populations. This is the same case for refractory and first-visit RA.

## Data Availability

The original contributions presented in the study are included in the article/Supplementary Material. Further inquiries can be directed to the corresponding authors.
