# Supplementary material for: Effectiveness and Safety of Iguratimod Monotherapy or Combined With Methotrexate in Treating Rheumatoid Arthritis: A Systematic Review and Meta-Analysis
Source: Front Pharmacol. 2022 Aug 5;13:911810. doi: 10.3389/fphar.2022.911810 (PMC9389904; doi:10.3389/fphar.2022.911810)
Supplement: Supplementary file 1 [file DataSheet1.ZIP › FIGURE S1.pdf]

	Experimental			Control				Mean Difference
Study or Subgroup	Mean	SD	Total	Mean	SD	Total	Weight	IV, Random, 95% CI
**12.1.1 SJC-28**								
Xia Z 2016	2.71	0.45	38	4.02	0.41	49	41.1%	-1.31 [-1.49, -1.13]
Qi D X 2019	6.2	5.3	40	6.5	5.2	40	21.9%	-0.30 [-2.60, 2.00]
Xu B J 2015	6.54	2.08	32	5.9	1.39	38	36.9%	0.64 [-0.21, 1.49]
**Subtotal (95% CI)**			**110**			**127**	**100.0%**	**-0.37 [-1.94, 1.20]**
Heterogeneity: Tau² = 1.56; Chi² = 20.13, df = 2 (P < 0.0001); I² = 90%								
Test for overall effect: Z = 0.46 (P = 0.65)								
**12.1.2 TJC-28**								
Xu B J 2015	4.89	1.19	32	7.59	1.23	38	42.9%	-2.70 [-3.27, -2.13]
Xia Z 2016	7.45	0.78	38	9.43	0.87	49	50.8%	-1.98 [-2.33, -1.63]
Qi D X 2019	8.2	6.8	40	8.3	5.9	40	6.4%	-0.10 [-2.89, 2.69]
**Subtotal (95% CI)**			**110**			**127**	**100.0%**	**-2.17 [-2.92, -1.42]**
Heterogeneity: Tau² = 0.25; Chi² = 6.59, df = 2 (P = 0.04); I² = 70%								
Test for overall effect: Z = 5.70 (P < 0.00001)								
**12.1.3 Morning stiffness**								
Xu B J 2015	27.11	3.56	38	32.96	3.81	49	49.0%	-5.85 [-7.41, -4.29]
Xia Z 2016	3.12	0.09	38	3.43	0.08	49	51.0%	-0.31 [-0.35, -0.27]
**Subtotal (95% CI)**			**76**			**98**	**100.0%**	**-3.02 [-8.45, 2.40]**
Heterogeneity: Tau² = 15.03; Chi² = 48.71, df = 1 (P < 0.00001); I² = 98%								
Test for overall effect: Z = 1.09 (P = 0.27)								
**12.1.4 ESR**								
Xiong Y M 2015	25	7	27	34	11	26	18.2%	-9.00 [-13.98, -4.02]
Xia Z 2016	19.84	1.46	38	26.27	1.15	49	37.7%	-6.43 [-6.99, -5.87]
Xu B J 2015	22.98	9.93	32	27.33	8.07	38	21.1%	-4.35 [-8.64, -0.06]
Zhao L 2017	21.97	13.05	34	21.28	19.46	33	10.0%	0.69 [-7.27, 8.65]
Qi D X 2019	34.5	17.4	40	33.4	12.5	40	13.0%	1.10 [-5.54, 7.74]
**Subtotal (95% CI)**			**171**			**186**	**100.0%**	**-4.77 [-7.71, -1.83]**
Heterogeneity: Tau² = 5.87; Chi² = 9.84, df = 4 (P = 0.04); I² = 59%								
Test for overall effect: Z = 3.18 (P = 0.001)								
**12.1.5 CRP**								
Zhao L 2017	6.92	1.62	34	19.9	7.39	33	20.1%	-12.98 [-15.56, -10.40]
Xiong Y M 2015	13	4	27	19	5	26	20.3%	-6.00 [-8.44, -3.56]
Xia Z 2016	9.25	1.07	38	14.99	1.44	49	22.4%	-5.74 [-6.27, -5.21]
Qi D X 2019	13.7	10.3	40	18.4	13	40	15.3%	-4.70 [-9.84, 0.44]
Xu B J 2015	5.82	1.32	32	6.15	3.76	38	21.9%	-0.33 [-1.61, 0.95]
**Subtotal (95% CI)**			**171**			**186**	**100.0%**	**-5.91 [-9.45, -2.37]**
Heterogeneity: Tau² = 14.49; Chi² = 94.97, df = 4 (P < 0.00001); I² = 96%								
Test for overall effect: Z = 3.27 (P = 0.001)								

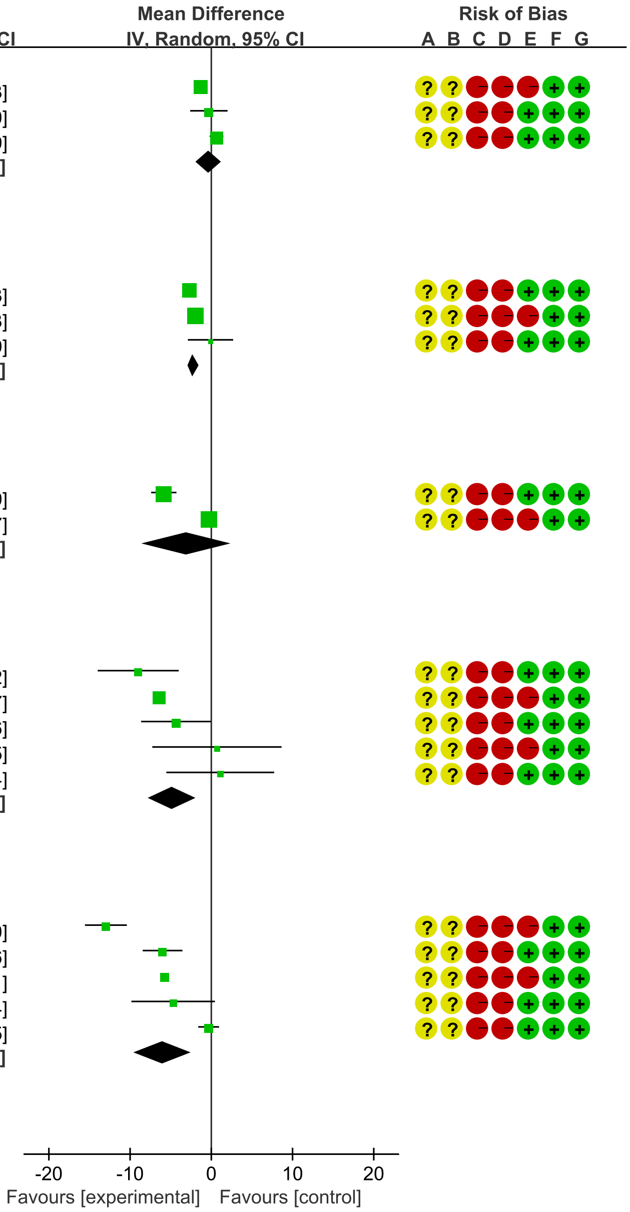

Test for subgroup differences: Chi² = 12.41, df = 4 (P = 0.01), I² = 67.8%

Risk of bias legend

(**A**) Random sequence generation (selection bias)
(**B**) Allocation concealment (selection bias)
(**C**) Blinding of participants and personnel (performance bias)
(**D**) Blinding of outcome assessment (detection bias)
(**E**) Incomplete outcome data (attrition bias)
(**F**) Selective reporting (reporting bias)
(**G**) Other bias
