# Supplementary material for: Effectiveness and Safety of Iguratimod Monotherapy or Combined With Methotrexate in Treating Rheumatoid Arthritis: A Systematic Review and Meta-Analysis
Source: Front Pharmacol. 2022 Aug 5;13:911810. doi: 10.3389/fphar.2022.911810 (PMC9389904; doi:10.3389/fphar.2022.911810)
Supplement: Supplementary file 1 [file DataSheet1.ZIP › S10.pdf]

Study or Subgroup	Experimental Mean	Experimental SD	Experimental Total	Control Mean	Control SD	Control Total	Weight	Mean Difference IV, Random, 95% CI	A	B	C	D	E	F	G
**9.1.1 IGU+MTX vs MTX**															
Jing J 2020	58.29	4.16	46	67.45	6.53	46	4.9%	-9.16 [-11.40, -6.92]	+	?	-	-	+	+	+
Li S Y 2019	1.24	0.11	40	1.78	0.67	40	14.7%	-0.54 [-0.75, -0.33]	+	?	-	-	+	+	+
Xia Z 2016	20.8	2.14	44	32.96	3.81	49	9.2%	-12.16 [-13.40, -10.92]	?	?	-	-	-	+	+
Xiong M L 2020	2.71	0.36	51	4.03	0.53	51	14.8%	-1.32 [-1.50, -1.14]	+	?	-	-	+	+	+
Xu B J 2015	2.32	1.58	30	4.1	2.44	30	10.4%	-1.78 [-2.82, -0.74]	?	?	-	-	+	+	+
Xu L M 2017	48.58	31.26	42	76.28	36.12	41	0.2%	-27.70 [-42.25, -13.15]	+	?	-	-	+	+	+
Zhao H N 2018	48.3	12.6	36	75.9	15.8	36	0.8%	-27.60 [-34.20, -21.00]	+	?	-	-	+	+	+
Zhao W Z 2021	0.55	0.06	52	1.24	0.12	52	15.0%	-0.69 [-0.73, -0.65]	+	?	-	-	+	+	+
**Subtotal (95% CI)**			**341**			**345**	**69.9%**	**-4.05 [-5.00, -3.09]**							
Heterogeneity: Tau² = 1.22; Chi² = 512.54, df = 7 (P < 0.00001); I² = 99%															
Test for overall effect: Z = 8.31 (P < 0.00001)															
**9.1.2 vs MTX+LEF**															
Niu M 2021	1.68	0.22	41	1.62	0.21	36	14.9%	0.06 [-0.04, 0.16]	+	?	-	-	+	+	+
Zhu L J 2017	57.28	16.53	64	61.27	18.46	64	0.9%	-3.99 [-10.06, 2.08]	+	?	-	-	+	+	+
**Subtotal (95% CI)**			**105**			**100**	**15.8%**	**-0.78 [-4.00, 2.44]**							
Heterogeneity: Tau² = 3.40; Chi² = 1.71, df = 1 (P = 0.19); I² = 41%															
Test for overall effect: Z = 0.48 (P = 0.63)															
**9.1.3 vs MTX+TGs**															
Li J Y 2021	1.07	0.46	30	1.53	0.84	30	14.3%	-0.46 [-0.80, -0.12]	?	?	-	-	+	+	+
**Subtotal (95% CI)**			**30**			**30**	**14.3%**	**-0.46 [-0.80, -0.12]**							
Heterogeneity: Not applicable															
Test for overall effect: Z = 2.63 (P = 0.009)															
**Total (95% CI)**			**476**			**475**	**100.0%**	**-2.49 [-3.09, -1.88]**							
Heterogeneity: Tau² = 0.63; Chi² = 740.32, df = 10 (P < 0.00001); I² = 99%															
Test for overall effect: Z = 8.07 (P < 0.00001)															
Test for subgroup differences: Chi² = 48.08, df = 2 (P < 0.00001), I² = 95.8%															

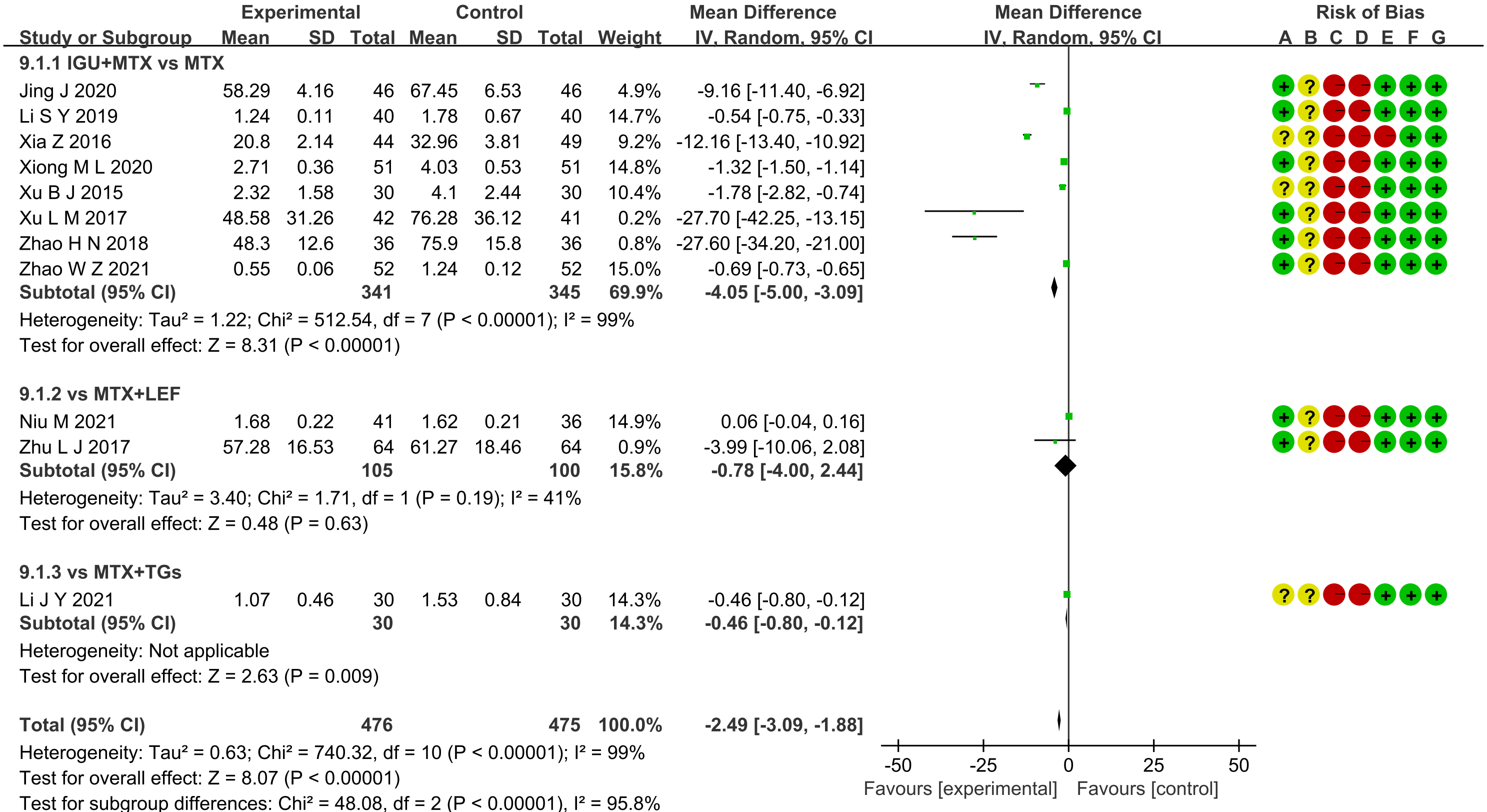

Risk of bias legend
(**A**) Random sequence generation (selection bias)
(**B**) Allocation concealment (selection bias)
(**C**) Blinding of participants and personnel (performance bias)
(**D**) Blinding of outcome assessment (detection bias)
(**E**) Incomplete outcome data (attrition bias)
(**F**) Selective reporting (reporting bias)
(**G**) Other bias
