# Supplementary material for: Effectiveness and Safety of Iguratimod Monotherapy or Combined With Methotrexate in Treating Rheumatoid Arthritis: A Systematic Review and Meta-Analysis
Source: Front Pharmacol. 2022 Aug 5;13:911810. doi: 10.3389/fphar.2022.911810 (PMC9389904; doi:10.3389/fphar.2022.911810)
Supplement: Supplementary file 1 [file DataSheet1.ZIP › S17.pdf]

Study or Subgroup	Experimental Events	Experimental Total	Control Events	Control Total	Weight	Risk Ratio M-H, Fixed, 95% CI	Risk Ratio M-H, Fixed, 95% CI	Risk of Bias A B C D E F G
**3.2.2 12w**								
Bai Q H 2015	16	50	7	50	7.3%	2.29 [1.03, 5.07]		
Mo H 2015	20	30	11	30	11.4%	1.82 [1.07, 3.10]		
Yan K H 2019	27	40	15	40	15.6%	1.80 [1.14, 2.83]		
**Subtotal (95% CI)**		**120**		**120**	**34.3%**	**1.91 [1.38, 2.64]**		
Total events	63		33					
Heterogeneity: Chi² = 0.29, df = 2 (P = 0.86); I² = 0%								
Test for overall effect: Z = 3.92 (P < 0.0001)								
**3.2.3 24w**								
Bai Q H 2015	21	50	11	50	11.4%	1.91 [1.03, 3.53]		
Duan X 2015	21	30	10	30	10.4%	2.10 [1.20, 3.67]		
Ishiguro N 2015	63	164	14	88	18.9%	2.41 [1.44, 4.05]		
Qi D X 2019	23	40	14	40	14.5%	1.64 [1.00, 2.71]		
Shi X D 2015	21	30	10	30	10.4%	2.10 [1.20, 3.67]		
**Subtotal (95% CI)**		**314**		**238**	**65.7%**	**2.06 [1.60, 2.64]**		
Total events	149		59					
Heterogeneity: Chi² = 1.21, df = 4 (P = 0.88); I² = 0%								
Test for overall effect: Z = 5.65 (P < 0.00001)								
**Total (95% CI)**		**434**		**358**	**100.0%**	**2.01 [1.64, 2.45]**		
Total events	212		92					
Heterogeneity: Chi² = 1.63, df = 7 (P = 0.98); I² = 0%								
Test for overall effect: Z = 6.88 (P < 0.00001)								
Test for subgroup differences: Chi² = 0.13, df = 1 (P = 0.72), I² = 0%								

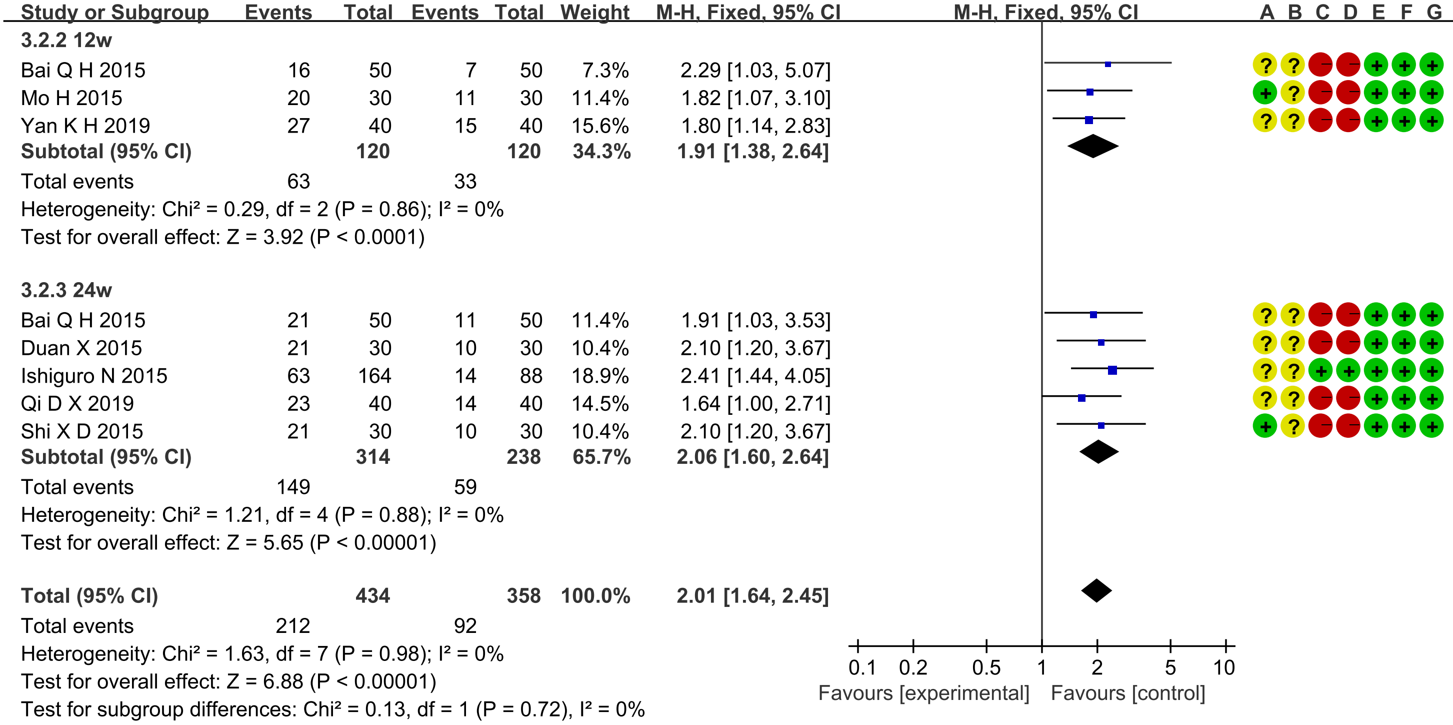

Risk of bias legend

(**A**) Random sequence generation (selection bias)
(**B**) Allocation concealment (selection bias)
(**C**) Blinding of participants and personnel (performance bias)
(**D**) Blinding of outcome assessment (detection bias)
(**E**) Incomplete outcome data (attrition bias)
(**F**) Selective reporting (reporting bias)
(**G**) Other bias
