# Supplementary material for: Effectiveness and Safety of Iguratimod Monotherapy or Combined With Methotrexate in Treating Rheumatoid Arthritis: A Systematic Review and Meta-Analysis
Source: Front Pharmacol. 2022 Aug 5;13:911810. doi: 10.3389/fphar.2022.911810 (PMC9389904; doi:10.3389/fphar.2022.911810)
Supplement: Supplementary file 1 [file DataSheet1.ZIP › S18.pdf]

| Study or Subgroup | Experimental Events | Experimental Total | Control Events | Control Total | Weight | Risk Ratio M-H, Fixed, 95% CI | A | B | C | D | E | F | G |
|---|---|---|---|---|---|---|---|---|---|---|---|---|---|
| **4.2.2 12w** | | | | | | | | | | | | | |
| Mo H 2015 | 16 | 30 | 8 | 30 | 19.7% | 2.00 [1.01, 3.95] | + | ? | - | - | + | + | + |
| Yan K H 2019 | 21 | 40 | 10 | 40 | 24.7% | 2.10 [1.14, 3.87] | ? | ? | - | - | + | + | + |
| **Subtotal (95% CI)** | | **70** | | **70** | **44.4%** | **2.06 [1.30, 3.24]** | | | | | | | |
| Total events | 37 | | 18 | | | | | | | | | | |
| Heterogeneity: Chi² = 0.01, df = 1 (P = 0.92); I² = 0% | | | | | | | | | | | | | |
| Test for overall effect: Z = 3.10 (P = 0.002) | | | | | | | | | | | | | |
| **4.2.3 24w** | | | | | | | | | | | | | |
| Duan X 2015 | 8 | 30 | 5 | 30 | 12.3% | 1.60 [0.59, 4.33] | ? | ? | - | - | + | + | + |
| Ishiguro N 2015 | 28 | 164 | 5 | 88 | 16.1% | 3.00 [1.20, 7.51] | ? | ? | + | + | + | + | + |
| Qi D X 2019 | 11 | 40 | 6 | 40 | 14.8% | 1.83 [0.75, 4.48] | ? | ? | - | - | + | + | + |
| Shi X D 2015 | 8 | 30 | 5 | 30 | 12.3% | 1.60 [0.59, 4.33] | + | ? | - | - | + | + | + |
| **Subtotal (95% CI)** | | **264** | | **188** | **55.6%** | **2.07 [1.28, 3.33]** | | | | | | | |
| Total events | 55 | | 21 | | | | | | | | | | |
| Heterogeneity: Chi² = 1.22, df = 3 (P = 0.75); I² = 0% | | | | | | | | | | | | | |
| Test for overall effect: Z = 2.98 (P = 0.003) | | | | | | | | | | | | | |
| **Total (95% CI)** | | **334** | | **258** | **100.0%** | **2.06 [1.48, 2.88]** | | | | | | | |
| Total events | 92 | | 39 | | | | | | | | | | |
| Heterogeneity: Chi² = 1.23, df = 5 (P = 0.94); I² = 0% | | | | | | | | | | | | | |
| Test for overall effect: Z = 4.25 (P < 0.0001) | | | | | | | | | | | | | |
| Test for subgroup differences: Chi² = 0.00, df = 1 (P = 0.99), I² = 0% | | | | | | | | | | | | | |

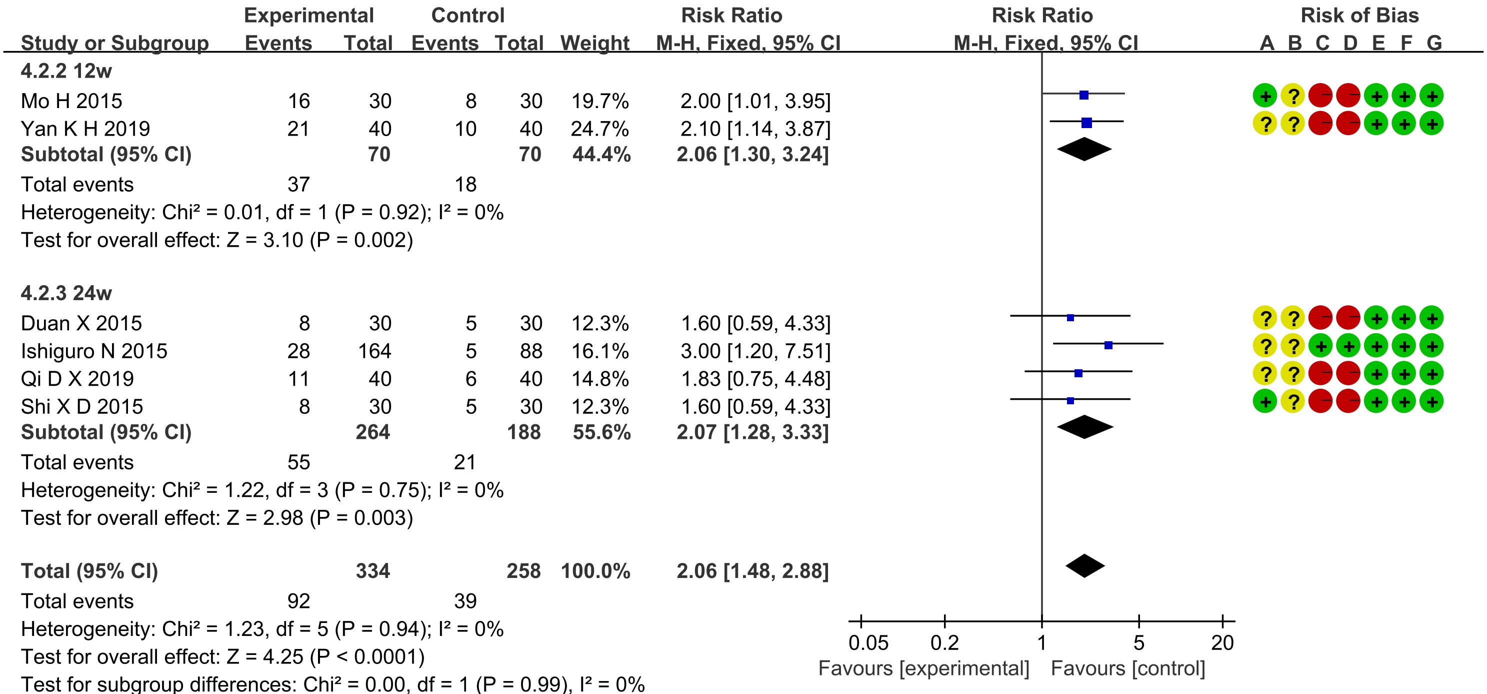


Risk of bias legend

(**A**) Random sequence generation (selection bias)
(**B**) Allocation concealment (selection bias)
(**C**) Blinding of participants and personnel (performance bias)
(**D**) Blinding of outcome assessment (detection bias)
(**E**) Incomplete outcome data (attrition bias)
(**F**) Selective reporting (reporting bias)
(**G**) Other bias
