# Supplementary material for: Effectiveness and Safety of Iguratimod Monotherapy or Combined With Methotrexate in Treating Rheumatoid Arthritis: A Systematic Review and Meta-Analysis
Source: Front Pharmacol. 2022 Aug 5;13:911810. doi: 10.3389/fphar.2022.911810 (PMC9389904; doi:10.3389/fphar.2022.911810)
Supplement: Supplementary file 1 [file DataSheet1.ZIP › S19.pdf]

	Experimental		Control			Risk Ratio	Risk of Bias						
Study or Subgroup	Events	Total	Events	Total	Weight	M-H, Fixed, 95% CI	A	B	C	D	E	F	G
**7.2.1 24w**													
Qi D X 2019	15	40	20	40	31.1%	0.75 [0.45, 1.24]	?	?	-	-	+	+	+
Zhu H 2019	6	30	11	30	17.1%	0.55 [0.23, 1.28]	?	?	-	-	+	+	+
**Subtotal (95% CI)**		**70**		**70**	**48.3%**	**0.68 [0.44, 1.05]**							
Total events	21		31										
Heterogeneity: Chi² = 0.40, df = 1 (P = 0.53); I² = 0%													
Test for overall effect: Z = 1.74 (P = 0.08)													
**7.2.2 52w**													
Du F 2021	41	297	33	293	51.7%	1.23 [0.80, 1.88]	?	?	+	+	+	+	+
**Subtotal (95% CI)**		**297**		**293**	**51.7%**	**1.23 [0.80, 1.88]**							
Total events	41		33										
Heterogeneity: Not applicable													
Test for overall effect: Z = 0.93 (P = 0.35)													
**Total (95% CI)**		**367**		**363**	**100.0%**	**0.96 [0.71, 1.31]**							
Total events	62		64										
Heterogeneity: Chi² = 3.84, df = 2 (P = 0.15); I² = 48%													
Test for overall effect: Z = 0.25 (P = 0.80)													
Test for subgroup differences: Chi² = 3.59, df = 1 (P = 0.06), I² = 72.1%													

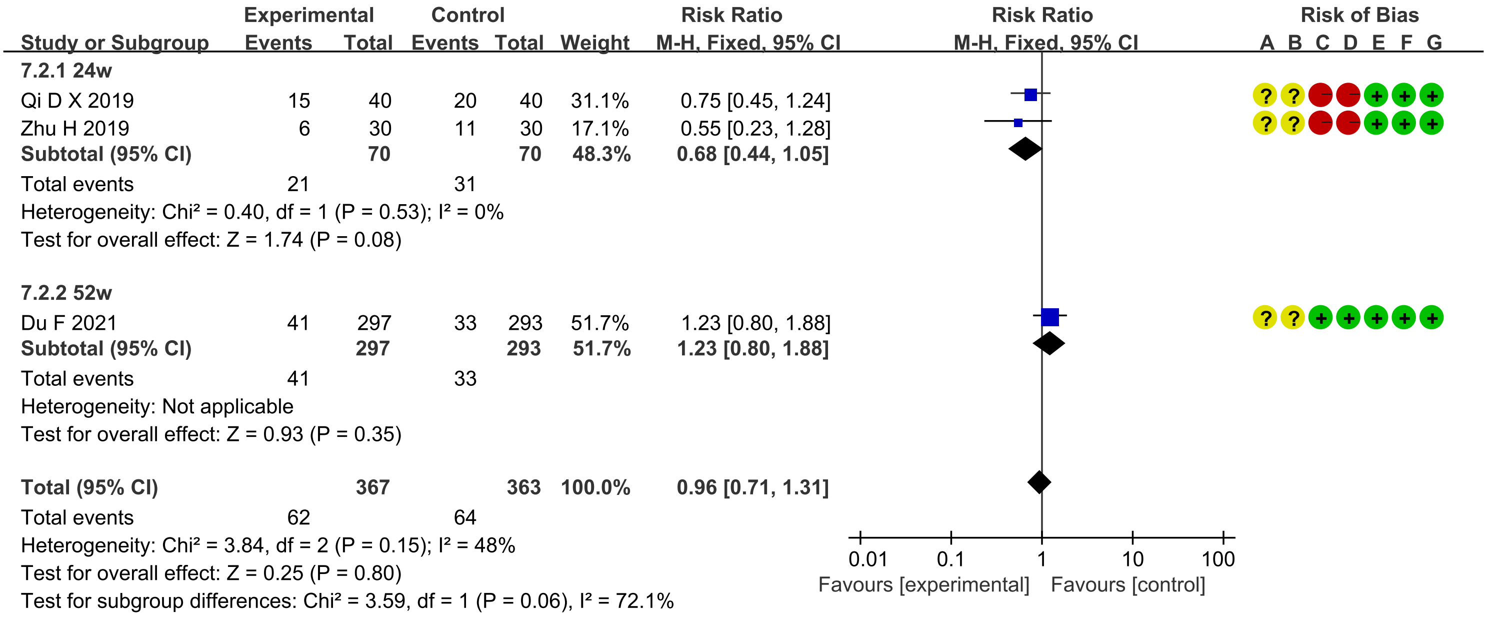

Risk of bias legend

(**A**) Random sequence generation (selection bias)
(**B**) Allocation concealment (selection bias)
(**C**) Blinding of participants and personnel (performance bias)
(**D**) Blinding of outcome assessment (detection bias)
(**E**) Incomplete outcome data (attrition bias)
(**F**) Selective reporting (reporting bias)
(**G**) Other bias
