# Supplementary material for: Effectiveness and Safety of Iguratimod Monotherapy or Combined With Methotrexate in Treating Rheumatoid Arthritis: A Systematic Review and Meta-Analysis
Source: Front Pharmacol. 2022 Aug 5;13:911810. doi: 10.3389/fphar.2022.911810 (PMC9389904; doi:10.3389/fphar.2022.911810)
Supplement: Supplementary file 1 [file DataSheet1.ZIP › S20.pdf]

Study or Subgroup	Experimental Events	Experimental Total	Control Events	Control Total	Weight	Risk Ratio M-H, Fixed, 95% CI
**8.2.2 12w**						
Mo H 2015	7	40	7	38	2.7%	0.95 [0.37, 2.45]
Mo M L 2018	2	30	3	30	1.1%	0.67 [0.12, 3.71]
Yan K H 2019	5	40	13	40	4.8%	0.38 [0.15, 0.98]
Zhao H N 2018	5	36	3	36	1.1%	1.67 [0.43, 6.46]
**Subtotal (95% CI)**		**146**		**144**	**9.8%**	**0.72 [0.42, 1.24]**
Total events	19		26			
Heterogeneity: Chi² = 3.54, df = 3 (P = 0.32); I² = 15%						
Test for overall effect: Z = 1.19 (P = 0.23)						
**8.2.3 24W**						
Bai Q H 2015	8	30	7	30	2.6%	1.14 [0.47, 2.75]
Chen B X 2021	3	30	6	30	2.2%	0.50 [0.14, 1.82]
Duan X 2015	7	30	6	30	2.2%	1.17 [0.44, 3.06]
Li J Y 2021	4	30	5	30	1.9%	0.80 [0.24, 2.69]
Liu C L 2020	6	73	8	73	3.0%	0.75 [0.27, 2.05]
Ma C 2017	11	32	15	32	5.6%	0.73 [0.40, 1.34]
Niu M 2021	6	64	15	64	5.6%	0.40 [0.17, 0.97]
Qi D X 2019	23	40	20	40	7.5%	1.15 [0.76, 1.73]
Shi X D 2015	19	50	14	50	5.2%	1.36 [0.77, 2.40]
Xiong M L 2020	10	51	7	51	2.6%	1.43 [0.59, 3.46]
Zhao W Z 2021	3	52	2	52	0.7%	1.50 [0.26, 8.61]
Zhu L J 2017	3	41	3	36	1.2%	0.88 [0.19, 4.08]
**Subtotal (95% CI)**		**523**		**518**	**40.3%**	**0.95 [0.76, 1.19]**
Total events	103		108			
Heterogeneity: Chi² = 9.43, df = 11 (P = 0.58); I² = 0%						
Test for overall effect: Z = 0.44 (P = 0.66)						
**8.2.4 52w**						
Du F 2021	35	305	33	293	12.5%	1.02 [0.65, 1.59]
Tian X P 2020	72	120	94	119	35.2%	0.76 [0.64, 0.90]
Xu B J 2015	7	30	6	30	2.2%	1.17 [0.44, 3.06]
**Subtotal (95% CI)**		**455**		**442**	**49.9%**	**0.84 [0.71, 1.01]**
Total events	114		133			
Heterogeneity: Chi² = 2.51, df = 2 (P = 0.28); I² = 20%						
Test for overall effect: Z = 1.90 (P = 0.06)						
**Total (95% CI)**		**1124**		**1104**	**100.0%**	**0.87 [0.76, 1.00]**
Total events	236		267			
Heterogeneity: Chi² = 17.75, df = 18 (P = 0.47); I² = 0%						
Test for overall effect: Z = 1.91 (P = 0.06)						
Test for subgroup differences: Chi² = 1.17, df = 2 (P = 0.56), I² = 0%						

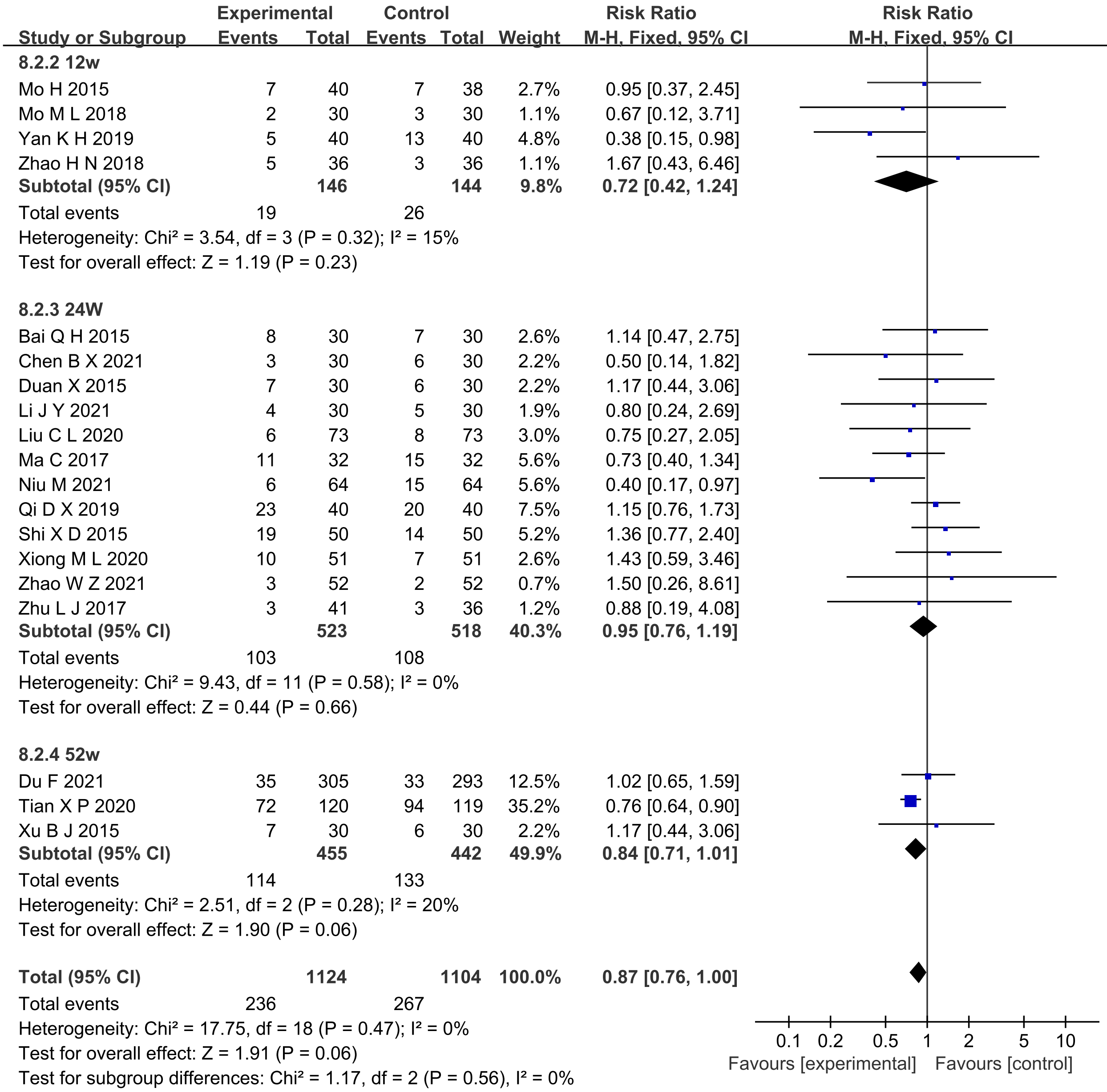

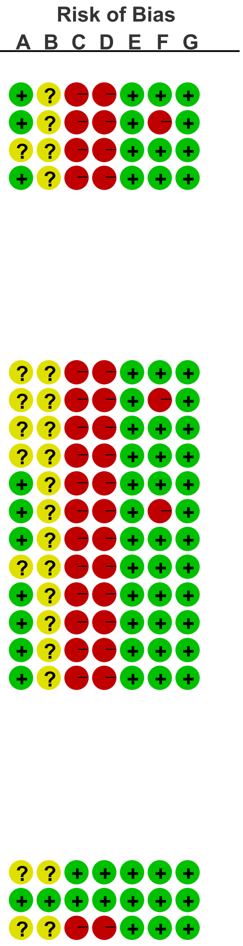

Risk of bias legend
(**A**) Random sequence generation (selection bias)
(**B**) Allocation concealment (selection bias)
(**C**) Blinding of participants and personnel (performance bias)
(**D**) Blinding of outcome assessment (detection bias)
(**E**) Incomplete outcome data (attrition bias)
(**F**) Selective reporting (reporting bias)
(**G**) Other bias
