# Supplementary material for: Effectiveness and Safety of Iguratimod Monotherapy or Combined With Methotrexate in Treating Rheumatoid Arthritis: A Systematic Review and Meta-Analysis
Source: Front Pharmacol. 2022 Aug 5;13:911810. doi: 10.3389/fphar.2022.911810 (PMC9389904; doi:10.3389/fphar.2022.911810)
Supplement: Supplementary file 1 [file DataSheet1.ZIP › S21.pdf]

Study or Subgroup	Experimental Mean	Experimental SD	Experimental Total	Control Mean	Control SD	Control Total	Weight	Mean Difference IV, Fixed, 95% CI	Risk of Bias A B C D E F G
**5.3.1 Refractory**									
Li J Y 2021	3.48	1.76	30	5.14	1.51	30	100.0%	-1.66 [-2.49, -0.83]	? ? − − + + +
**Subtotal (95% CI)**			**30**			**30**	**100.0%**	**-1.66 [-2.49, -0.83]**	
Heterogeneity: Not applicable									
Test for overall effect: Z = 3.92 (P < 0.0001)									
**Total (95% CI)**			**30**			**30**	**100.0%**	**-1.66 [-2.49, -0.83]**	
Heterogeneity: Not applicable									
Test for overall effect: Z = 3.92 (P < 0.0001)									
Test for subgroup differences: Not applicable									

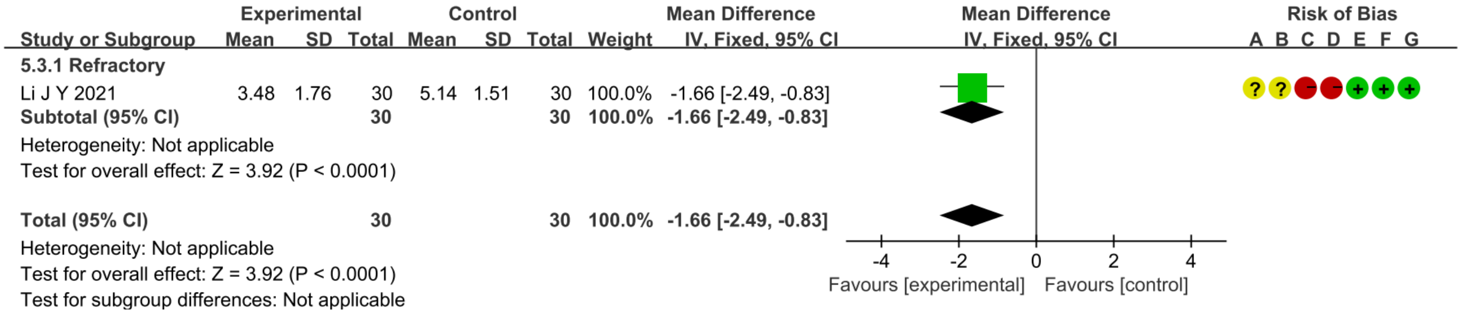

Study or Subgroup	Experimental Events	Experimental Total	Control Events	Control Total	Weight	Risk Ratio M-H, Fixed, 95% CI	Risk of Bias A B C D E F G
**1.3.1 ACR20_First-visit**							
Du F 2021	230	297	193	293	100.0%	1.18 [1.06, 1.30]	? ? + + + + +
**Subtotal (95% CI)**		**297**		**293**	**100.0%**	**1.18 [1.06, 1.30]**	
Total events	230		193				
Heterogeneity: Not applicable							
Test for overall effect: Z = 3.09 (P = 0.002)							
**Total (95% CI)**		**297**		**293**	**100.0%**	**1.18 [1.06, 1.30]**	
Total events	230		193				
Heterogeneity: Not applicable							
Test for overall effect: Z = 3.09 (P = 0.002)							
Test for subgroup differences: Not applicable							

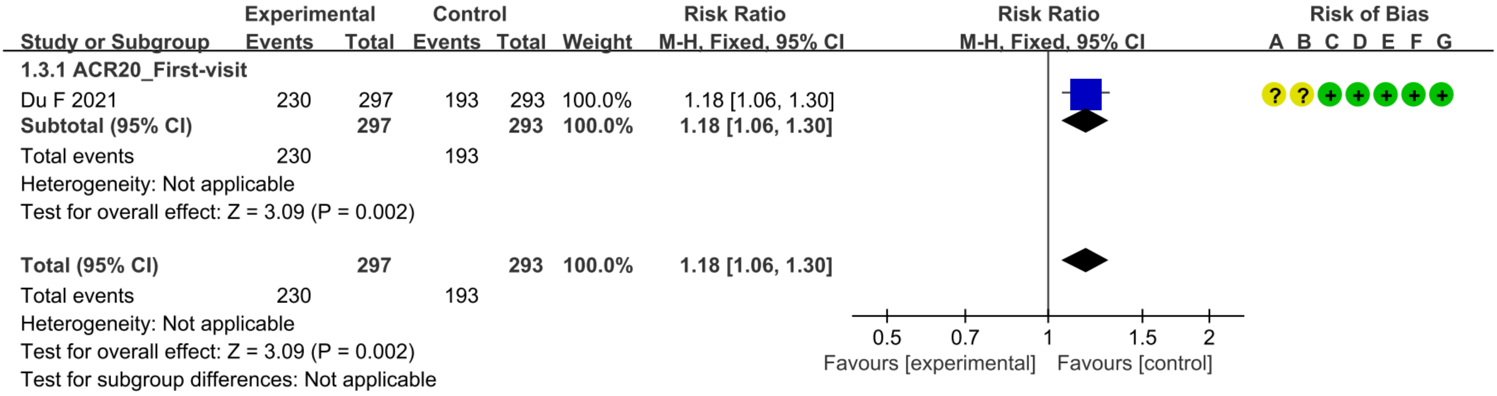

Study or Subgroup	Experimental Events	Experimental Total	Control Events	Control Total	Weight	Risk Ratio M-H, Fixed, 95% CI	Risk of Bias A B C D E F G
**7.4.1 AEs-First-visit**							
Du F 2021	41	297	33	293	100.0%	1.23 [0.80, 1.88]	? ? + + + + +
**Subtotal (95% CI)**		**297**		**293**	**100.0%**	**1.23 [0.80, 1.88]**	
Total events	41		33				
Heterogeneity: Not applicable							
Test for overall effect: Z = 0.93 (P = 0.35)							
**Total (95% CI)**		**297**		**293**	**100.0%**	**1.23 [0.80, 1.88]**	
Total events	41		33				
Heterogeneity: Not applicable							
Test for overall effect: Z = 0.93 (P = 0.35)							
Test for subgroup differences: Not applicable							

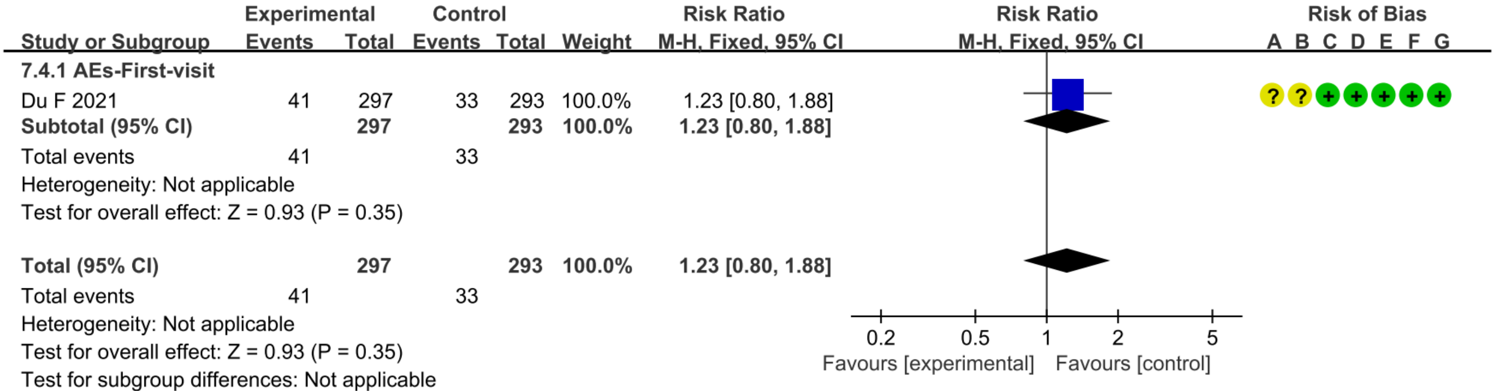

Risk of bias legend

(**A**) Random sequence generation (selection bias)
(**B**) Allocation concealment (selection bias)
(**C**) Blinding of participants and personnel (performance bias)
(**D**) Blinding of outcome assessment (detection bias)
(**E**) Incomplete outcome data (attrition bias)
(**F**) Selective reporting (reporting bias)
(**G**) Other bias
