# Supplementary material for: Effectiveness and Safety of Iguratimod Monotherapy or Combined With Methotrexate in Treating Rheumatoid Arthritis: A Systematic Review and Meta-Analysis
Source: Front Pharmacol. 2022 Aug 5;13:911810. doi: 10.3389/fphar.2022.911810 (PMC9389904; doi:10.3389/fphar.2022.911810)
Supplement: Supplementary file 1 [file DataSheet1.ZIP › S23.pdf]

Study or Subgroup	Experimental Mean	Experimental SD	Experimental Total	Control Mean	Control SD	Control Total	Weight	Mean Difference IV, Random, 95% CI	Risk of Bias A B C D E F G
**5.4.1 DAS28-ESR_Elderly**									
Cao L N 2018	3.79	0.2	30	3.93	0.26	30	100.0%	-0.14 [-0.26, -0.02]	+ ? - - + + +
**Subtotal (95% CI)**			**30**			**30**	**100.0%**	**-0.14 [-0.26, -0.02]**	
Heterogeneity: Not applicable									
Test for overall effect: Z = 2.34 (P = 0.02)									
**Total (95% CI)**			**30**			**30**	**100.0%**	**-0.14 [-0.26, -0.02]**	
Heterogeneity: Not applicable									
Test for overall effect: Z = 2.34 (P = 0.02)									
Test for subgroup differences: Not applicable									

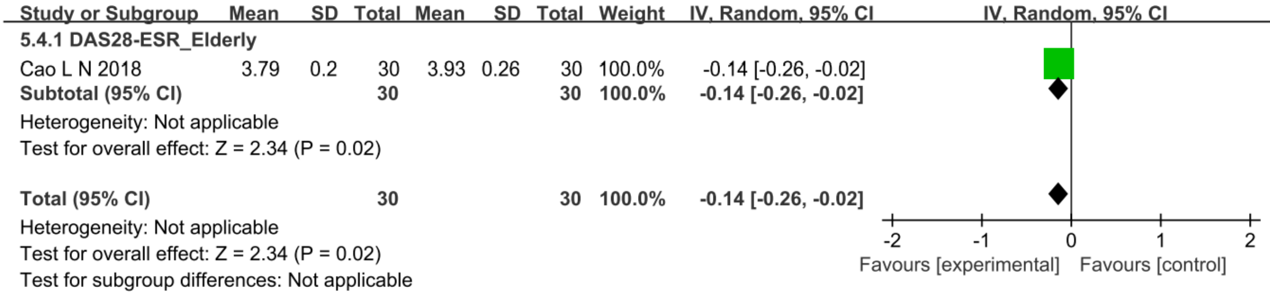

Study or Subgroup	Experimental Events	Experimental Total	Control Events	Control Total	Weight	Risk Ratio M-H, Fixed, 95% CI	Risk of Bias A B C D E F G
**1.4.1 ACR20_Elderly**							
Zhu H 2019	22	30	20	30	51.3%	1.10 [0.79, 1.53]	? ? - - + + +
**Subtotal (95% CI)**		**30**		**30**	**51.3%**	**1.10 [0.79, 1.53]**	
Total events	22		20				
Heterogeneity: Not applicable							
Test for overall effect: Z = 0.56 (P = 0.57)							
**1.4.2 Young and middle aged**							
Qi D X 2019	20	40	19	40	48.7%	1.05 [0.67, 1.65]	? ? - - + + +
**Subtotal (95% CI)**		**40**		**40**	**48.7%**	**1.05 [0.67, 1.65]**	
Total events	20		19				
Heterogeneity: Not applicable							
Test for overall effect: Z = 0.22 (P = 0.82)							
**Total (95% CI)**		**70**		**70**	**100.0%**	**1.08 [0.82, 1.42]**	
Total events	42		39				
Heterogeneity: Chi² = 0.03, df = 1 (P = 0.87); I² = 0%							
Test for overall effect: Z = 0.52 (P = 0.60)							
Test for subgroup differences: Chi² = 0.02, df = 1 (P = 0.88), I² = 0%							

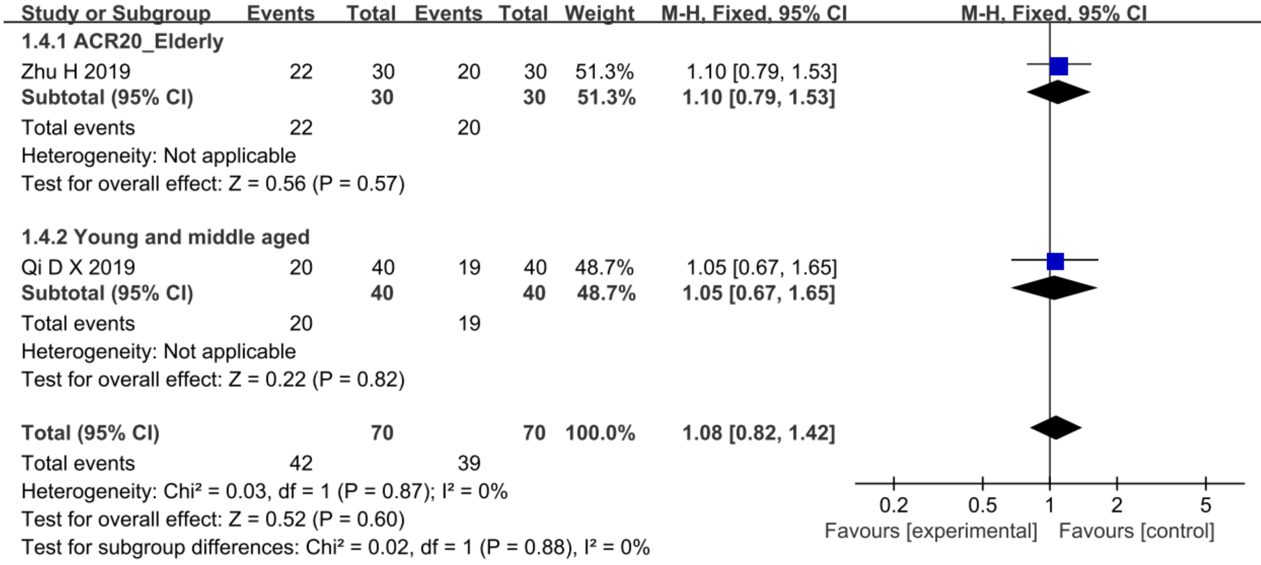

Study or Subgroup	Experimental Events	Experimental Total	Control Events	Control Total	Weight	Risk Ratio M-H, Fixed, 95% CI	Risk of Bias A B C D E F G
**10.3.1 ACR50_Elderly**							
Zhu H 2019	14	30	13	30	48.1%	1.08 [0.62, 1.89]	? ? - - + + +
**Subtotal (95% CI)**		**30**		**30**	**48.1%**	**1.08 [0.62, 1.89]**	
Total events	14		13				
Heterogeneity: Not applicable							
Test for overall effect: Z = 0.26 (P = 0.80)							
**10.3.2 Young and middle aged**							
Qi D X 2019	13	40	14	40	51.9%	0.93 [0.50, 1.72]	? ? - - + + +
**Subtotal (95% CI)**		**40**		**40**	**51.9%**	**0.93 [0.50, 1.72]**	
Total events	13		14				
Heterogeneity: Not applicable							
Test for overall effect: Z = 0.24 (P = 0.81)							
**Total (95% CI)**		**70**		**70**	**100.0%**	**1.00 [0.66, 1.52]**	
Total events	27		27				
Heterogeneity: Chi² = 0.12, df = 1 (P = 0.73); I² = 0%							
Test for overall effect: Z = 0.00 (P = 1.00)							
Test for subgroup differences: Chi² = 0.12, df = 1 (P = 0.73), I² = 0%							

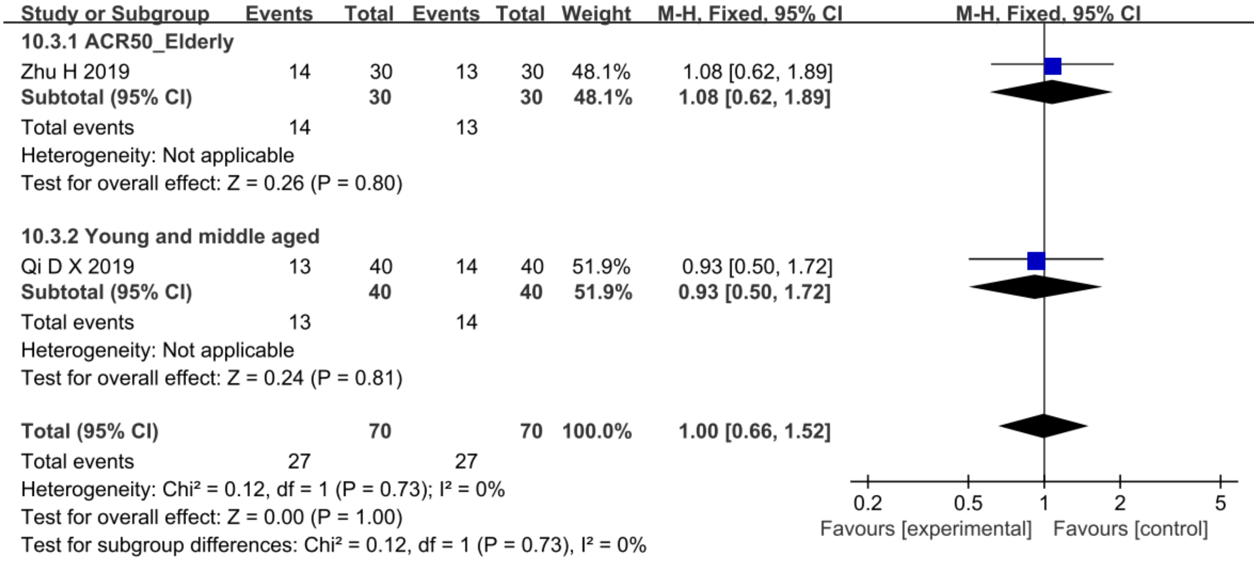

Risk of bias legend

(**A**) Random sequence generation (selection bias)
(**B**) Allocation concealment (selection bias)
(**C**) Blinding of participants and personnel (performance bias)
(**D**) Blinding of outcome assessment (detection bias)
(**E**) Incomplete outcome data (attrition bias)
(**F**) Selective reporting (reporting bias)
(**G**) Other bias
