# Supplementary material for: Effectiveness and Safety of Iguratimod Monotherapy or Combined With Methotrexate in Treating Rheumatoid Arthritis: A Systematic Review and Meta-Analysis
Source: Front Pharmacol. 2022 Aug 5;13:911810. doi: 10.3389/fphar.2022.911810 (PMC9389904; doi:10.3389/fphar.2022.911810)
Supplement: Supplementary file 1 [file DataSheet1.ZIP › S24.pdf]

Study or Subgroup	Experimental Events	Experimental Total	Control Events	Control Total	Weight	Risk Ratio M-H, Fixed, 95% CI	Risk of Bias A B C D E F G
**11.2.1 ACR70_Elderly**							
Zhu H 2019	5	30	6	30	50.0%	0.83 [0.28, 2.44]	? ? − − + + +
**Subtotal (95% CI)**		**30**		**30**	**50.0%**	**0.83 [0.28, 2.44]**	
Total events	5		6				
Heterogeneity: Not applicable							
Test for overall effect: Z = 0.33 (P = 0.74)							
**11.2.2 Young and middle-aged**							
Qi D X 2019	6	40	6	40	50.0%	1.00 [0.35, 2.84]	? ? − − + + +
**Subtotal (95% CI)**		**40**		**40**	**50.0%**	**1.00 [0.35, 2.84]**	
Total events	6		6				
Heterogeneity: Not applicable							
Test for overall effect: Z = 0.00 (P = 1.00)							
**Total (95% CI)**		**70**		**70**	**100.0%**	**0.92 [0.43, 1.94]**	
Total events	11		12				
Heterogeneity: Chi² = 0.06, df = 1 (P = 0.81); I² = 0%							
Test for overall effect: Z = 0.23 (P = 0.82)							
Test for subgroup differences: Chi² = 0.06, df = 1 (P = 0.81), I² = 0%							

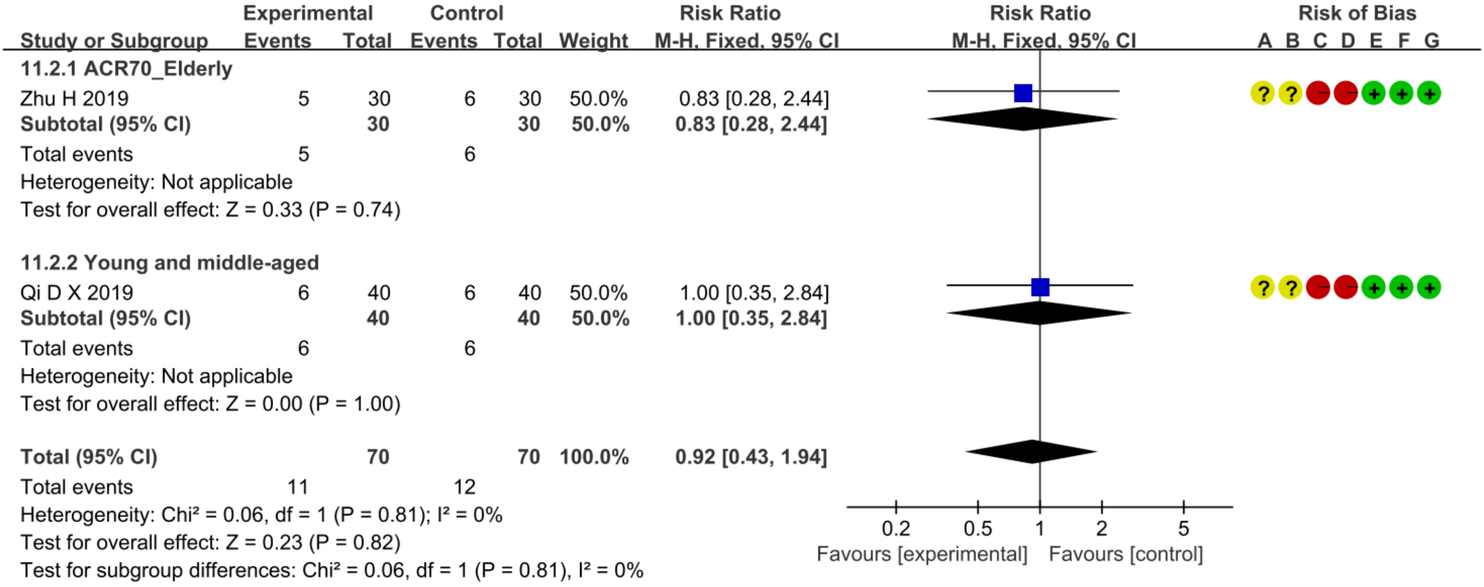

Study or Subgroup	Experimental Events	Experimental Total	Control Events	Control Total	Weight	Risk Ratio M-H, Fixed, 95% CI	Risk of Bias A B C D E F G
**7.3.1 AEs_Young and middle-aged**							
Qi D X 2019	15	40	20	40	64.5%	0.75 [0.45, 1.24]	? ? − − + + +
**Subtotal (95% CI)**		**40**		**40**	**64.5%**	**0.75 [0.45, 1.24]**	
Total events	15		20				
Heterogeneity: Not applicable							
Test for overall effect: Z = 1.11 (P = 0.27)							
**7.3.2 Elderly**							
Zhu H 2019	6	30	11	30	35.5%	0.55 [0.23, 1.28]	? ? − − + + +
**Subtotal (95% CI)**		**30**		**30**	**35.5%**	**0.55 [0.23, 1.28]**	
Total events	6		11				
Heterogeneity: Not applicable							
Test for overall effect: Z = 1.39 (P = 0.17)							
**Total (95% CI)**		**70**		**70**	**100.0%**	**0.68 [0.44, 1.05]**	
Total events	21		31				
Heterogeneity: Chi² = 0.40, df = 1 (P = 0.53); I² = 0%							
Test for overall effect: Z = 1.74 (P = 0.08)							
Test for subgroup differences: Chi² = 0.39, df = 1 (P = 0.53), I² = 0%							

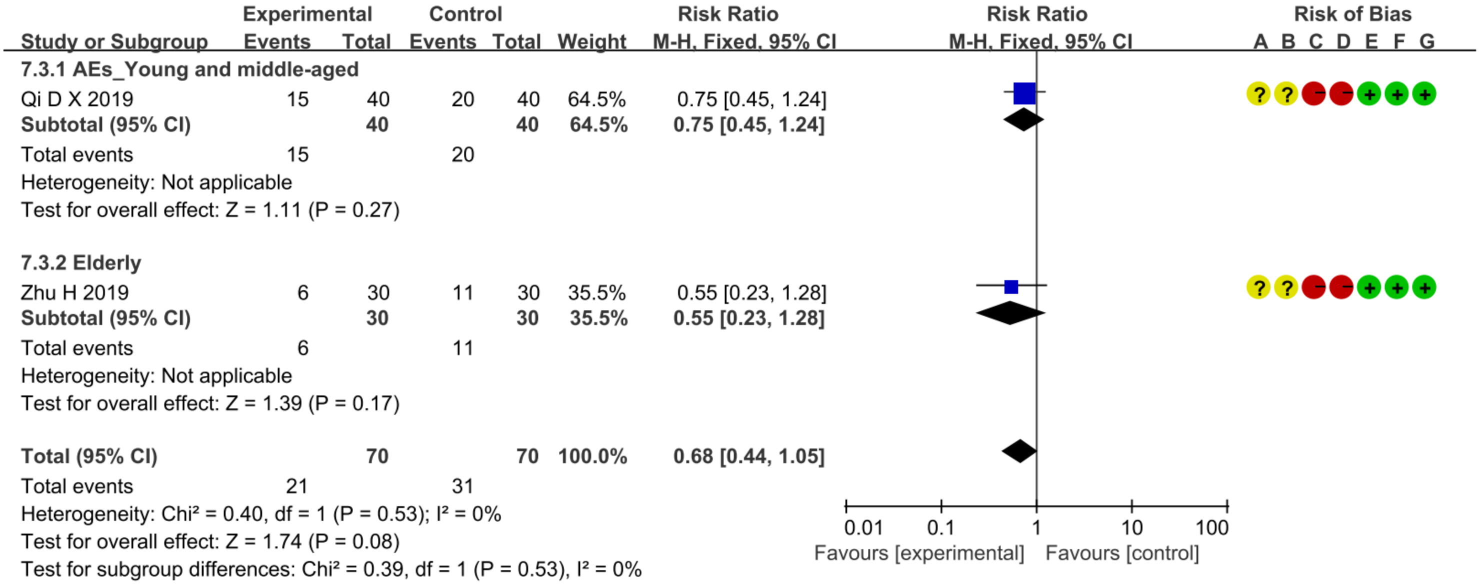

Risk of bias legend
(**A**) Random sequence generation (selection bias)
(**B**) Allocation concealment (selection bias)
(**C**) Blinding of participants and personnel (performance bias)
(**D**) Blinding of outcome assessment (detection bias)
(**E**) Incomplete outcome data (attrition bias)
(**F**) Selective reporting (reporting bias)
(**G**) Other bias
