# Supplementary material for: Effectiveness and Safety of Iguratimod Monotherapy or Combined With Methotrexate in Treating Rheumatoid Arthritis: A Systematic Review and Meta-Analysis
Source: Front Pharmacol. 2022 Aug 5;13:911810. doi: 10.3389/fphar.2022.911810 (PMC9389904; doi:10.3389/fphar.2022.911810)
Supplement: Supplementary file 1 [file DataSheet1.ZIP › S26.pdf]

Study or Subgroup	Experimental Events	Experimental Total	Control Events	Control Total	Weight	Risk Ratio M-H, Fixed, 95% CI	A	B	C	D	E	F	G
**8.5.1 leukopenia**													
Chen X Y 2018	3	40	2	40	5.9%	1.50 [0.26, 8.50]	+	?	-	-	+	+	+
Duan X 2015	2	30	1	30	3.0%	2.00 [0.19, 20.90]	?	?	-	-	+	+	+
Liu C L 2020	1	73	1	73	3.0%	1.00 [0.06, 15.69]	+	?	-	-	+	+	+
Mo H 2015	3	40	3	38	9.1%	0.95 [0.20, 4.42]	+	?	-	-	+	+	+
Niu M 2021	1	64	2	64	5.9%	0.50 [0.05, 5.38]	+	?	-	-	+	+	+
Qi D X 2019	4	40	3	40	8.9%	1.33 [0.32, 5.58]	?	?	-	-	+	+	+
Tian X P 2020	4	120	15	119	44.7%	0.26 [0.09, 0.77]	+	+	+	+	+	+	+
Xie L 2018	0	39	1	39	4.5%	0.33 [0.01, 7.94]	?	?	-	-	+	+	+
Yan K H 2019	1	40	2	40	5.9%	0.50 [0.05, 5.30]	?	?	-	-	+	+	+
Zhao L 2017	2	35	3	34	9.0%	0.65 [0.12, 3.64]	?	?	-	-	-	+	+
**Subtotal (95% CI)**		**521**		**517**	**100.0%**	**0.63 [0.37, 1.07]**							
Total events	21		33										
Heterogeneity: Chi² = 6.06, df = 9 (P = 0.73); I² = 0%													
Test for overall effect: Z = 1.69 (P = 0.09)													
**8.5.2 LFTs**													
Chen B X 2021	2	30	1	30	1.2%	2.00 [0.19, 20.90]	?	?	-	-	+	-	+
Chen X Y 2018	1	40	1	40	1.2%	1.00 [0.06, 15.44]	+	?	-	-	+	+	+
Duan X 2015	3	30	2	30	2.4%	1.50 [0.27, 8.34]	?	?	-	-	+	+	+
Liu C L 2020	1	73	1	73	1.2%	1.00 [0.06, 15.69]	+	?	-	-	+	+	+
Ma C 2017	1	32	3	32	3.5%	0.33 [0.04, 3.04]	+	?	-	-	+	-	+
Meng D Q 2015	1	33	0	33	0.6%	3.00 [0.13, 71.07]	+	?	-	-	+	+	+
Mo H 2015	2	40	2	38	2.4%	0.95 [0.14, 6.41]	+	?	-	-	+	+	+
Mo M L 2018	1	30	2	30	2.4%	0.50 [0.05, 5.22]	+	?	-	-	+	-	+
Niu M 2021	1	64	4	64	4.7%	0.25 [0.03, 2.18]	+	?	-	-	+	+	+
Qi D X 2019	6	40	5	40	5.9%	1.20 [0.40, 3.62]	?	?	-	-	+	+	+
Tian X P 2020	20	120	50	119	59.0%	0.40 [0.25, 0.62]	+	+	+	+	+	+	+
Xie L 2018	0	39	1	39	1.8%	0.33 [0.01, 7.94]	?	?	-	-	+	+	+
Yan K H 2019	1	40	3	40	3.5%	0.33 [0.04, 3.07]	?	?	-	-	+	+	+
Zhao L 2017	6	35	5	34	6.0%	1.17 [0.39, 3.46]	?	?	-	-	-	+	+
Zhu L J 2017	0	41	3	36	4.4%	0.13 [0.01, 2.36]	+	?	-	-	+	+	+
**Subtotal (95% CI)**		**687**		**678**	**100.0%**	**0.56 [0.40, 0.77]**							
Total events	46		83										
Heterogeneity: Chi² = 11.99, df = 14 (P = 0.61); I² = 0%													
Test for overall effect: Z = 3.55 (P = 0.0004)													

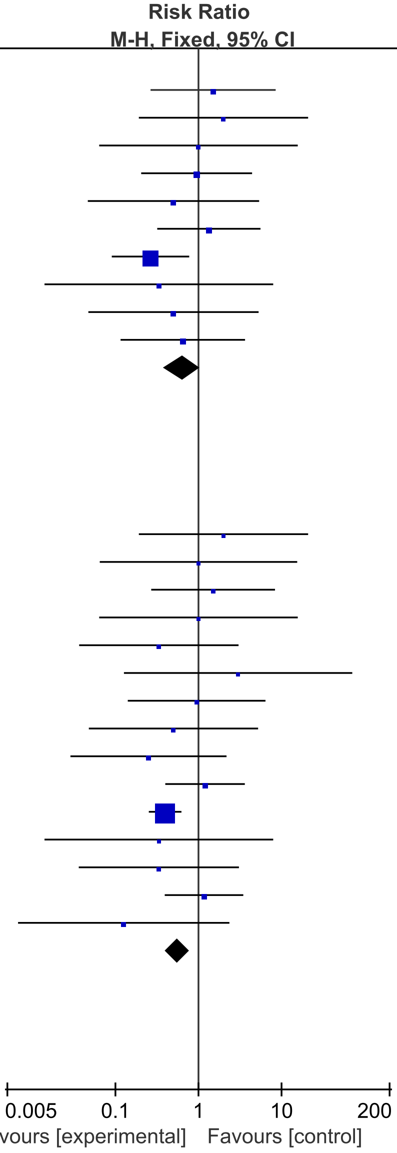

Test for subgroup differences: Chi² = 0.18, df = 1 (P = 0.67), I² = 0%

Risk of bias legend

(**A**) Random sequence generation (selection bias)
(**B**) Allocation concealment (selection bias)
(**C**) Blinding of participants and personnel (performance bias)
(**D**) Blinding of outcome assessment (detection bias)
(**E**) Incomplete outcome data (attrition bias)
(**F**) Selective reporting (reporting bias)
(**G**) Other bias
