# Supplementary material for: Effectiveness and Safety of Iguratimod Monotherapy or Combined With Methotrexate in Treating Rheumatoid Arthritis: A Systematic Review and Meta-Analysis
Source: Front Pharmacol. 2022 Aug 5;13:911810. doi: 10.3389/fphar.2022.911810 (PMC9389904; doi:10.3389/fphar.2022.911810)
Supplement: Supplementary file 1 [file DataSheet1.ZIP › S4.pdf]

Study or Subgroup	Experimental Mean	Experimental SD	Experimental Total	Control Mean	Control SD	Control Total	Weight	Mean Difference IV, Random, 95% CI	A	B	C	D	E	F	G
**9.3.1 IGU+MTX vs MTX**															
Jing J 2020	4.35	0.69	46	7.36	0.94	46	9.0%	-3.01 [-3.35, -2.67]	+	?	−	−	+	+	+
Qi D X 2019	4	3.8	40	6.5	5.2	40	6.6%	-2.50 [-4.50, -0.50]	?	?	−	−	+	+	+
Xia Z 2016	1.09	0.15	44	4.02	0.41	49	9.1%	-2.93 [-3.05, -2.81]	?	?	−	−	−	+	+
Xiong M L 2020	9.67	1.29	51	13.18	2.04	51	8.7%	-3.51 [-4.17, -2.85]	+	?	−	−	+	+	+
Xu B J 2015	4.39	0.32	30	7.59	1.23	30	8.9%	-3.20 [-3.65, -2.75]	?	?	−	−	+	+	+
**Subtotal (95% CI)**			**211**			**216**	**42.2%**	**-2.98 [-3.11, -2.85]**							
Heterogeneity: Tau² = 0.00; Chi² = 4.21, df = 4 (P = 0.38); I² = 5%															
Test for overall effect: Z = 45.14 (P < 0.00001)															
**9.3.2 vs MTX+HCQ**															
Liu C L 2020	2.15	0.87	73	3.45	1.34	73	9.0%	-1.30 [-1.67, -0.93]	+	?	−	−	+	+	+
**Subtotal (95% CI)**			**73**			**73**	**9.0%**	**-1.30 [-1.67, -0.93]**							
Heterogeneity: Not applicable															
Test for overall effect: Z = 6.95 (P < 0.00001)															
**9.3.3 vs MTX + LEF**															
Ma C 2017	1.08	1.28	32	2.14	1.87	32	8.6%	-1.06 [-1.85, -0.27]	+	?	−	−	+	−	+
Niu M 2021	1.25	0.36	64	1.34	0.4	64	9.1%	-0.09 [-0.22, 0.04]	+	?	−	−	+	+	+
Tian X P 2020	2	8.72	82	2	9.74	62	4.8%	0.00 [-3.07, 3.07]	+	+	+	+	+	+	+
Zhu L J 2017	1.15	0.32	41	1.04	0.31	36	9.1%	0.11 [-0.03, 0.25]	+	?	−	−	+	+	+
**Subtotal (95% CI)**			**219**			**194**	**31.4%**	**-0.09 [-0.36, 0.18]**							
Heterogeneity: Tau² = 0.04; Chi² = 11.07, df = 3 (P = 0.01); I² = 73%															
Test for overall effect: Z = 0.68 (P = 0.50)															
**9.3.4 vs MTX+TGs**															
Li J Y 2021	2.12	1.08	30	4.45	1.38	30	8.7%	-2.33 [-2.96, -1.70]	?	?	−	−	+	+	+
Xia N N 2020	4.82	1.55	50	6.47	1.7	50	8.7%	-1.65 [-2.29, -1.01]	+	?	−	−	+	+	+
**Subtotal (95% CI)**			**80**			**80**	**17.5%**	**-1.99 [-2.66, -1.33]**							
Heterogeneity: Tau² = 0.13; Chi² = 2.22, df = 1 (P = 0.14); I² = 55%															
Test for overall effect: Z = 5.86 (P < 0.00001)															
**Total (95% CI)**			**583**			**563**	**100.0%**	**-1.84 [-2.81, -0.87]**							
Heterogeneity: Tau² = 2.70; Chi² = 1605.01, df = 11 (P < 0.00001); I² = 99%															
Test for overall effect: Z = 3.72 (P = 0.0002)															
Test for subgroup differences: Chi² = 393.42, df = 3 (P < 0.00001), I² = 99.2%															

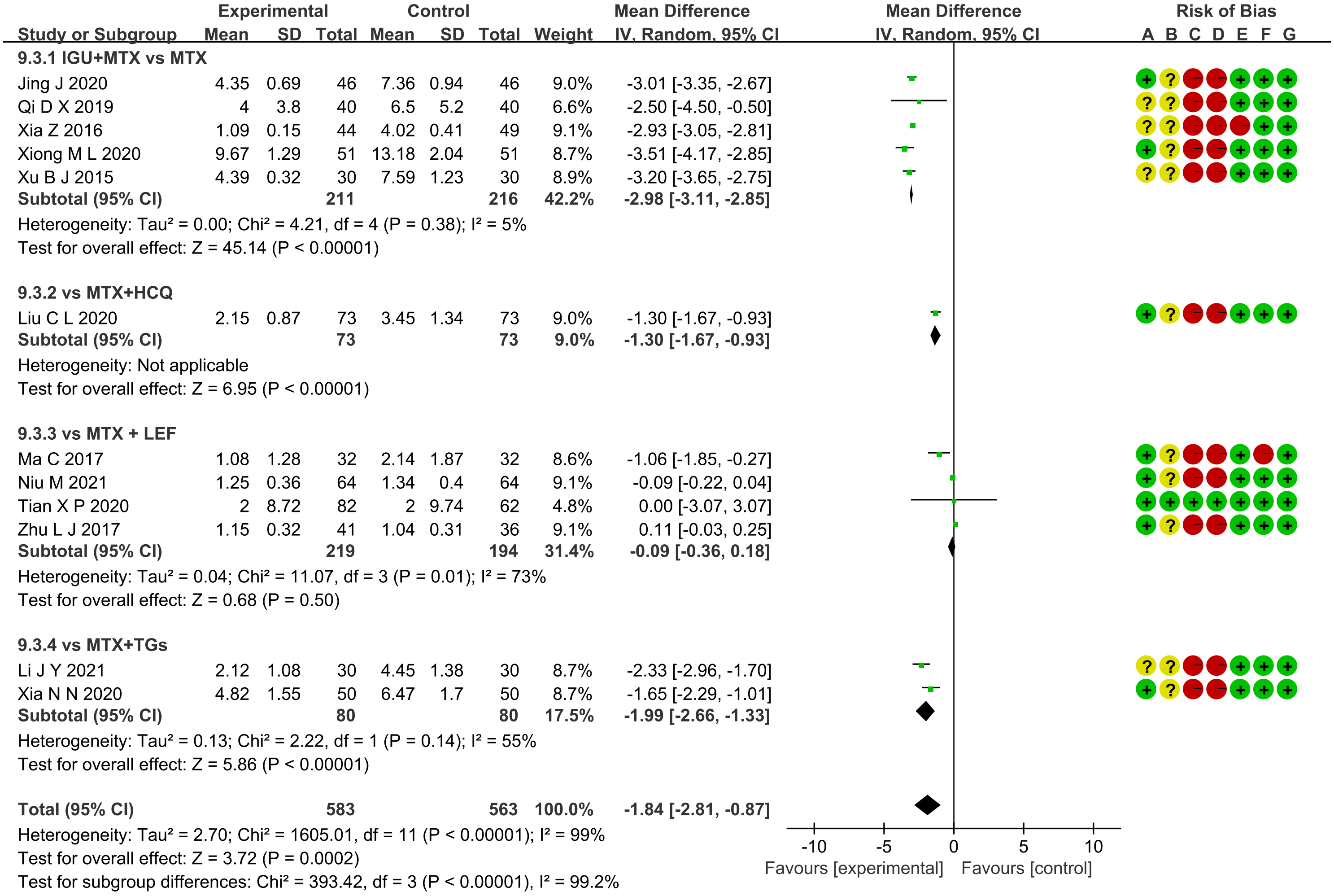

Risk of bias legend

(**A**) Random sequence generation (selection bias)
(**B**) Allocation concealment (selection bias)
(**C**) Blinding of participants and personnel (performance bias)
(**D**) Blinding of outcome assessment (detection bias)
(**E**) Incomplete outcome data (attrition bias)
(**F**) Selective reporting (reporting bias)
(**G**) Other bias
