# Supplementary material for: Effectiveness and Safety of Iguratimod Monotherapy or Combined With Methotrexate in Treating Rheumatoid Arthritis: A Systematic Review and Meta-Analysis
Source: Front Pharmacol. 2022 Aug 5;13:911810. doi: 10.3389/fphar.2022.911810 (PMC9389904; doi:10.3389/fphar.2022.911810)
Supplement: Supplementary file 1 [file DataSheet1.ZIP › S6.pdf]

Study or Subgroup	Experimental Mean	Experimental SD	Experimental Total	Control Mean	Control SD	Control Total	Weight	Mean Difference IV, Random, 95% CI
**9.9.1 IGU+MTX vs MTX**								
Jing J 2020	27.53	3.79	46	34.26	4.61	46	6.2%	-6.73 [-8.45, -5.01]
Ju Y J 2020	11.44	2.14	58	32.84	6.15	58	6.2%	-21.40 [-23.08, -19.72]
Li S Y 2019	34.56	3.21	40	45.72	12.53	40	5.9%	-11.16 [-15.17, -7.15]
Mo H 2015	38.52	29.36	30	51.86	30.53	30	3.3%	-13.34 [-28.50, 1.82]
Qi D X 2019	19.9	12.1	40	33.4	12.5	40	5.6%	-13.50 [-18.89, -8.11]
Shi X D 2015	14.3	9.5	30	25.4	22	30	4.9%	-11.10 [-19.68, -2.52]
Xia Z 2016	10.03	4.33	27	35.87	6.65	28	6.0%	-25.84 [-28.80, -22.88]
Xiong Y M 2015	15	6	29	34	11	26	5.7%	-19.00 [-23.76, -14.24]
Xu L M 2017	30.22	5.59	42	51.11	8.94	41	6.0%	-20.89 [-24.11, -17.67]
Zhao H N 2018	30.4	5.8	36	51.3	9.6	36	5.9%	-20.90 [-24.56, -17.24]
Zhao L 2017	39.14	14.7	29	39.06	16.48	33	5.1%	0.08 [-7.68, 7.84]
**Subtotal (95% CI)**			**407**			**408**	**60.7%**	**-15.27 [-20.31, -10.23]**
Heterogeneity: Tau² = 64.02; Chi² = 234.92, df = 10 (P < 0.00001); I² = 96%								
Test for overall effect: Z = 5.94 (P < 0.00001)								
**9.9.2 vs MTX+LEF**								
Ma C 2017	20.79	19.11	32	33.41	21.02	32	4.5%	-12.62 [-22.46, -2.78]
Niu M 2021	32.59	5.86	64	34.24	6.88	64	6.1%	-1.65 [-3.86, 0.56]
Tian X P 2020	28	23	74	23	22	58	5.1%	5.00 [-2.71, 12.71]
**Subtotal (95% CI)**			**170**			**154**	**15.7%**	**-2.37 [-9.64, 4.90]**
Heterogeneity: Tau² = 29.66; Chi² = 7.63, df = 2 (P = 0.02); I² = 74%								
Test for overall effect: Z = 0.64 (P = 0.52)								
**9.9.3 vs MTX+HCQ**								
Liu C L 2020	30.14	4.11	73	36.75	5.42	73	6.2%	-6.61 [-8.17, -5.05]
**Subtotal (95% CI)**			**73**			**73**	**6.2%**	**-6.61 [-8.17, -5.05]**
Heterogeneity: Not applicable								
Test for overall effect: Z = 8.30 (P < 0.00001)								
**9.9.4 vs MTX+ TGs**								
Li J Y 2021	24.47	6.32	30	29.44	5.01	30	6.0%	-4.97 [-7.86, -2.08]
Mo M L 2018	25.35	12.35	30	27.36	14.38	30	5.3%	-2.01 [-8.79, 4.77]
Xia N N 2020	19.15	6.44	50	27.2	6.51	50	6.1%	-8.05 [-10.59, -5.51]
**Subtotal (95% CI)**			**110**			**110**	**17.4%**	**-5.91 [-8.86, -2.95]**
Heterogeneity: Tau² = 3.41; Chi² = 4.17, df = 2 (P = 0.12); I² = 52%								
Test for overall effect: Z = 3.92 (P < 0.0001)								
**Total (95% CI)**			**760**			**745**	**100.0%**	**-10.98 [-15.01, -6.96]**
Heterogeneity: Tau² = 67.71; Chi² = 495.91, df = 17 (P < 0.00001); I² = 97%								
Test for overall effect: Z = 5.35 (P < 0.00001)								
Test for subgroup differences: Chi² = 12.66, df = 3 (P = 0.005), I² = 76.3%								

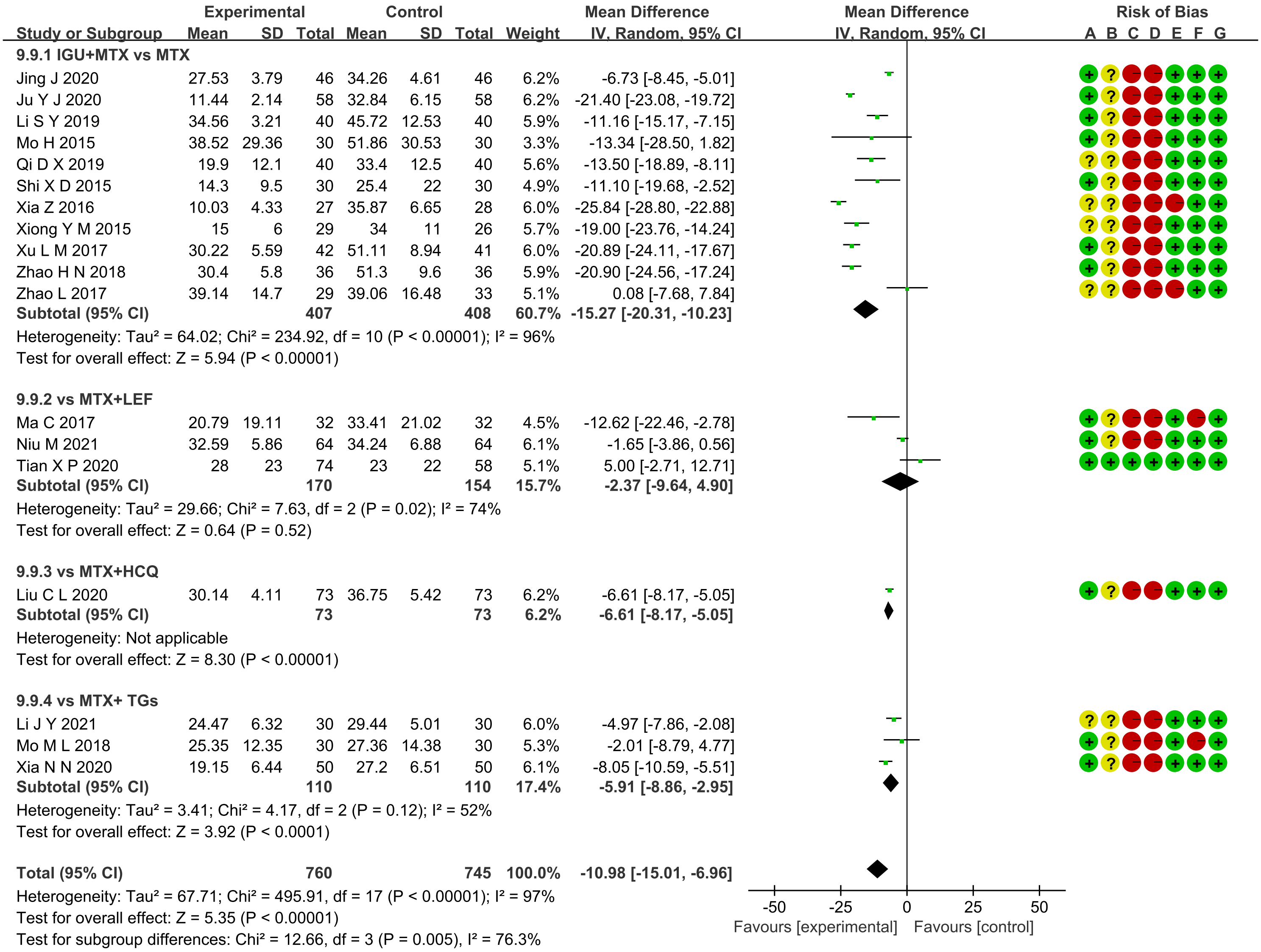

Risk of bias legend
(**A**) Random sequence generation (selection bias)
(**B**) Allocation concealment (selection bias)
(**C**) Blinding of participants and personnel (performance bias)
(**D**) Blinding of outcome assessment (detection bias)
(**E**) Incomplete outcome data (attrition bias)
(**F**) Selective reporting (reporting bias)
(**G**) Other bias
