# Supplementary material for: Effectiveness and Safety of Iguratimod Monotherapy or Combined With Methotrexate in Treating Rheumatoid Arthritis: A Systematic Review and Meta-Analysis
Source: Front Pharmacol. 2022 Aug 5;13:911810. doi: 10.3389/fphar.2022.911810 (PMC9389904; doi:10.3389/fphar.2022.911810)
Supplement: Supplementary file 1 [file DataSheet1.ZIP › S7.pdf]

Study or Subgroup	Experimental Mean	Experimental SD	Experimental Total	Control Mean	Control SD	Control Total	Weight	Mean Difference IV, Random, 95% CI	A	B	C	D	E	F	G
**9.10.1 IGU+MTX vs MTX**															
Ishiguro N 2015	9.3	20.8	30	2.6	19.7	30	3.3%	6.70 [-3.55, 16.95]	?	?	+	+	+	+	+
Jing J 2020	14.91	2.09	46	28.41	4.05	46	6.4%	-13.50 [-14.82, -12.18]	+	?	-	-	+	+	+
Ju Y J 2020	9.13	1.82	58	27.47	7.24	58	6.3%	-18.34 [-20.26, -16.42]	+	?	-	-	+	+	+
Li S Y 2019	10.19	2.31	40	14.14	4.12	40	6.4%	-3.95 [-5.41, -2.49]	+	?	-	-	+	+	+
Mo H 2015	30.65	30.81	30	48.25	31.83	30	2.0%	-17.60 [-33.45, -1.75]	+	?	-	-	+	+	+
Qi D X 2019	11.6	12.1	40	18.4	13	40	5.1%	-6.80 [-12.30, -1.30]	?	?	-	-	+	+	+
Shi X D 2015	5.7	5.6	30	10.7	9	30	5.7%	-5.00 [-8.79, -1.21]	+	?	-	-	+	+	+
Xia Z 2016	9.03	4.25	27	27.19	5.34	28	6.1%	-18.16 [-20.71, -15.61]	?	?	-	-	-	+	+
Xiong Y M 2015	8	3	29	19	5	26	6.2%	-11.00 [-13.21, -8.79]	?	?	-	-	+	+	+
Xu L M 2017	22.18	7.71	42	34.43	9.29	41	5.8%	-12.25 [-15.93, -8.57]	+	?	-	-	+	+	+
Zhao H N 2018	22.3	7.6	36	34.5	9.7	36	5.7%	-12.20 [-16.23, -8.17]	+	?	-	-	+	+	+
Zhao L 2017	5.69	0.8	29	19.9	7.39	33	6.1%	-14.21 [-16.75, -11.67]	?	?	-	-	-	+	+
**Subtotal (95% CI)**			**437**			**438**	**65.1%**	**-10.87 [-14.31, -7.43]**							
Heterogeneity: Tau² = 31.09; Chi² = 217.59, df = 11 (P < 0.00001); I² = 95%															
Test for overall effect: Z = 6.20 (P < 0.00001)															
**9.10.2 vs MTX+LEF**															
Ma C 2017	12.07	12.63	32	13.14	14.32	32	4.6%	-1.07 [-7.69, 5.55]	+	?	-	-	+	-	+
Tian X P 2020	13	19	76	8	16	59	4.9%	5.00 [-0.91, 10.91]	+	+	+	+	+	+	+
**Subtotal (95% CI)**			**108**			**91**	**9.6%**	**2.15 [-3.78, 8.09]**							
Heterogeneity: Tau² = 8.18; Chi² = 1.80, df = 1 (P = 0.18); I² = 44%															
Test for overall effect: Z = 0.71 (P = 0.48)															
**9.10.3 vs MTX+HCQ**															
Chen B X 2021	7.81	2.58	30	10.6	2.25	30	6.4%	-2.79 [-4.01, -1.57]	?	?	-	-	+	-	+
Liu C L 2020	3.06	0.21	73	8.49	2.59	73	6.5%	-5.43 [-6.03, -4.83]	+	?	-	-	+	+	+
**Subtotal (95% CI)**			**103**			**103**	**12.9%**	**-4.17 [-6.75, -1.58]**							
Heterogeneity: Tau² = 3.24; Chi² = 14.43, df = 1 (P = 0.0001); I² = 93%															
Test for overall effect: Z = 3.16 (P = 0.002)															
**9.10.4 vs MTX + TGs**															
Li J Y 2021	12.75	3.48	30	15.46	4.45	30	6.3%	-2.71 [-4.73, -0.69]	?	?	-	-	+	+	+
Mo M L 2018	9.45	4.32	30	11.78	5.56	30	6.1%	-2.33 [-4.85, 0.19]	+	?	-	-	+	-	+
**Subtotal (95% CI)**			**60**			**60**	**12.4%**	**-2.56 [-4.14, -0.98]**							
Heterogeneity: Tau² = 0.00; Chi² = 0.05, df = 1 (P = 0.82); I² = 0%															
Test for overall effect: Z = 3.18 (P = 0.001)															
**Total (95% CI)**			**708**			**692**	**100.0%**	**-7.75 [-10.41, -5.08]**							
Heterogeneity: Tau² = 28.41; Chi² = 506.81, df = 17 (P < 0.00001); I² = 97%															
Test for overall effect: Z = 5.70 (P < 0.00001)															
Test for subgroup differences: Chi² = 22.58, df = 3 (P < 0.0001), I² = 86.7%															

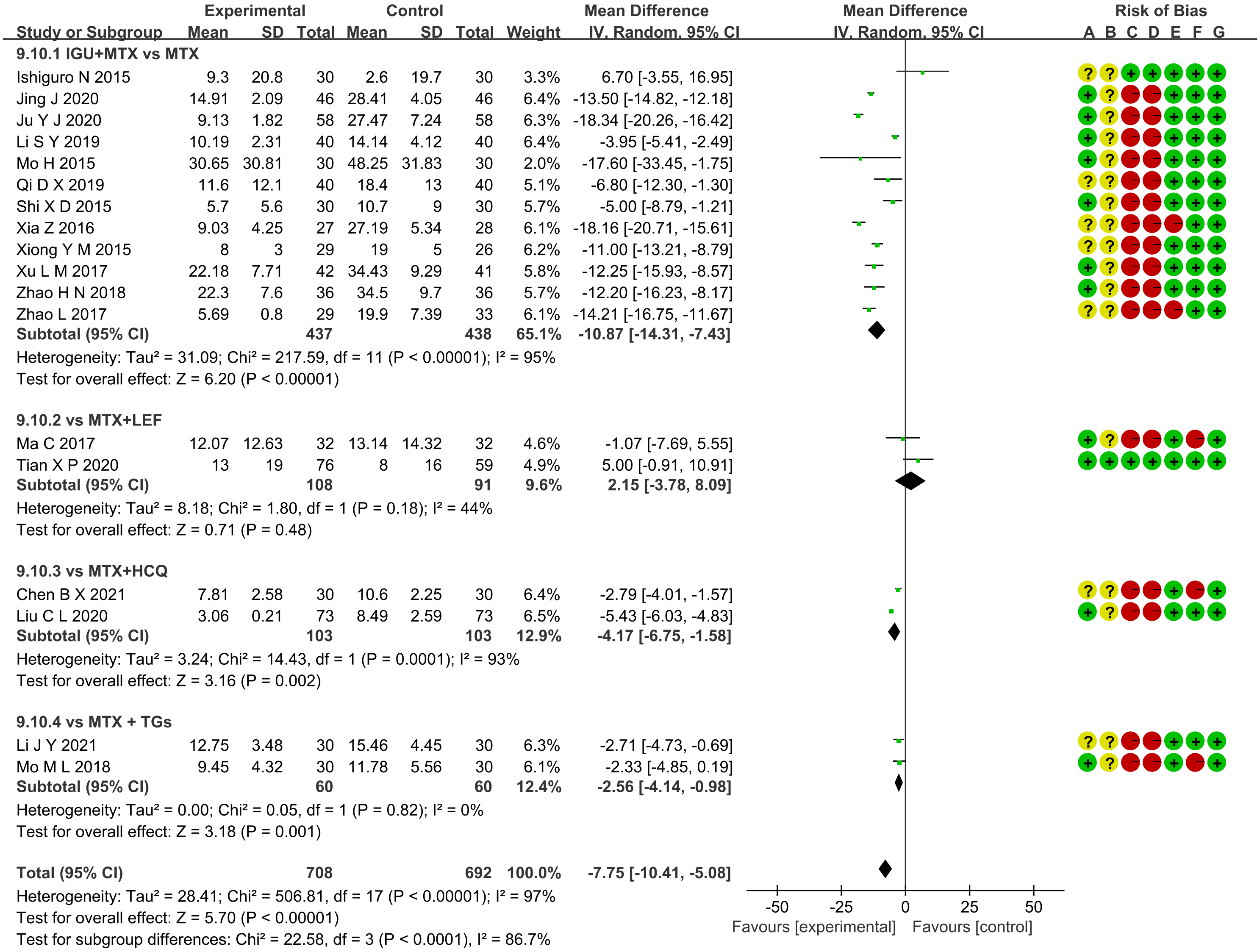

Risk of bias legend

(**A**) Random sequence generation (selection bias)
(**B**) Allocation concealment (selection bias)
(**C**) Blinding of participants and personnel (performance bias)
(**D**) Blinding of outcome assessment (detection bias)
(**E**) Incomplete outcome data (attrition bias)
(**F**) Selective reporting (reporting bias)
(**G**) Other bias
