# Supplementary material for: Effectiveness and Safety of Iguratimod Monotherapy or Combined With Methotrexate in Treating Rheumatoid Arthritis: A Systematic Review and Meta-Analysis
Source: Front Pharmacol. 2022 Aug 5;13:911810. doi: 10.3389/fphar.2022.911810 (PMC9389904; doi:10.3389/fphar.2022.911810)
Supplement: Supplementary file 1 [file DataSheet1.ZIP › S8.pdf]

Study or Subgroup	Experimental Mean	Experimental SD	Experimental Total	Control Mean	Control SD	Control Total	Weight	Mean Difference IV, Fixed, 95% CI
**9.11.1 IGU+MTX vs MTX**								
Ishiguro N 2015	37.4	63	30	31.7	190.9	30	0.5%	5.70 [-66.24, 77.64]
Mo H 2015	36.85	25.63	30	52.26	28.35	30	14.1%	-15.41 [-29.09, -1.73]
Xiong Y M 2015	25	12	29	43	9	26	84.8%	-18.00 [-23.57, -12.43]
**Subtotal (95% CI)**			**89**			**86**	**99.3%**	**-17.51 [-22.66, -12.37]**
Heterogeneity: Chi² = 0.52, df = 2 (P = 0.77); I² = 0%								
Test for overall effect: Z = 6.67 (P < 0.00001)								
**9.11.2 vs MTX+LEF**								
Tian X P 2020	9	475	79	134	451	63	0.1%	-125.00 [-277.88, 27.88]
**Subtotal (95% CI)**			**79**			**63**	**0.1%**	**-125.00 [-277.88, 27.88]**
Heterogeneity: Not applicable								
Test for overall effect: Z = 1.60 (P = 0.11)								
**9.11.3 vs MTX+ TGs**								
Mo M L 2018	290.65	120.32	30	300.68	148.76	30	0.6%	-10.03 [-78.49, 58.43]
**Subtotal (95% CI)**			**30**			**30**	**0.6%**	**-10.03 [-78.49, 58.43]**
Heterogeneity: Not applicable								
Test for overall effect: Z = 0.29 (P = 0.77)								
**Total (95% CI)**			**198**			**179**	**100.0%**	**-17.59 [-22.72, -12.46]**
Heterogeneity: Chi² = 2.46, df = 4 (P = 0.65); I² = 0%								
Test for overall effect: Z = 6.72 (P < 0.00001)								
Test for subgroup differences: Chi² = 1.94, df = 2 (P = 0.38), I² = 0%								

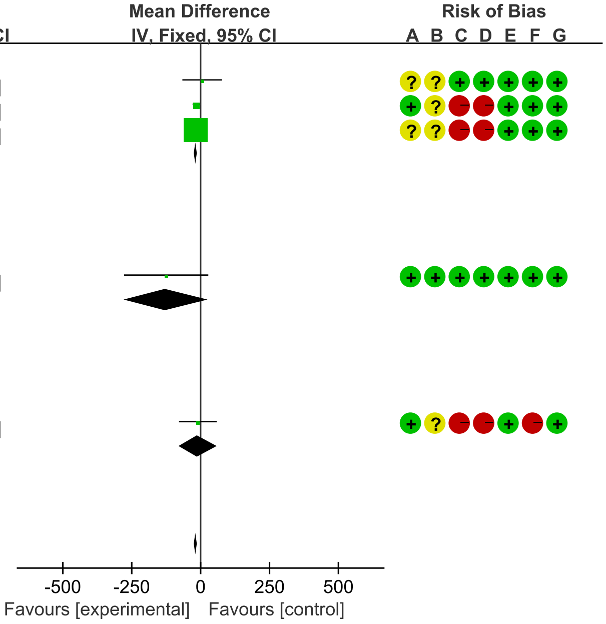

Risk of bias legend
(**A**) Random sequence generation (selection bias)
(**B**) Allocation concealment (selection bias)
(**C**) Blinding of participants and personnel (performance bias)
(**D**) Blinding of outcome assessment (detection bias)
(**E**) Incomplete outcome data (attrition bias)
(**F**) Selective reporting (reporting bias)
(**G**) Other bias
