# Supplementary material for: Effectiveness and Safety of Iguratimod Monotherapy or Combined With Methotrexate in Treating Rheumatoid Arthritis: A Systematic Review and Meta-Analysis
Source: Front Pharmacol. 2022 Aug 5;13:911810. doi: 10.3389/fphar.2022.911810 (PMC9389904; doi:10.3389/fphar.2022.911810)
Supplement: Supplementary file 1 [file DataSheet1.ZIP › S9.pdf]

Study or Subgroup	Experimental Mean	Experimental SD	Experimental Total	Control Mean	Control SD	Control Total	Weight	Mean Difference IV, Random, 95% CI	A	B	C	D	E	F	G
**9.12.1 IGU+MTX vs MTX**															
Ju Y J 2020	13.12	3.22	58	46.8	5.33	58	22.8%	-33.68 [-35.28, -32.08]	+	?	-	-	+	+	+
Mo H 2015	63.58	32.83	30	92.85	33.63	30	12.0%	-29.27 [-46.09, -12.45]	+	?	-	-	+	+	+
Xia Z 2016	12.15	5.75	27	43.52	5.3	28	22.4%	-31.37 [-34.30, -28.44]	?	?	-	-	-	+	+
Xiong Y M 2015	56	11	29	83	15	26	19.8%	-27.00 [-34.02, -19.98]	?	?	-	-	+	+	+
**Subtotal (95% CI)**			**144**			**142**	**77.0%**	**-32.11 [-34.54, -29.68]**							
Heterogeneity: Tau² = 2.22; Chi² = 4.85, df = 3 (P = 0.18); I² = 38%															
Test for overall effect: Z = 25.91 (P < 0.00001)															
**9.12.2 vs MTX+HCQ**															
Liu C L 2020	61.85	10.25	73	82.35	12.34	73	22.0%	-20.50 [-24.18, -16.82]	+	?	-	-	+	+	+
**Subtotal (95% CI)**			**73**			**73**	**22.0%**	**-20.50 [-24.18, -16.82]**							
Heterogeneity: Not applicable															
Test for overall effect: Z = 10.92 (P < 0.00001)															
**9.12.3 vs MTX+TGs**															
Mo M L 2018	126.23	45.36	30	480.63	223.23	30	1.0%	-354.40 [-435.91, -272.89]	+	?	-	-	+	-	+
**Subtotal (95% CI)**			**30**			**30**	**1.0%**	**-354.40 [-435.91, -272.89]**							
Heterogeneity: Not applicable															
Test for overall effect: Z = 8.52 (P < 0.00001)															
**Total (95% CI)**			**247**			**245**	**100.0%**	**-31.66 [-40.06, -23.26]**							

Heterogeneity: Tau² = 79.84; Chi² = 103.36, df = 5 (P < 0.00001); I² = 95%
Test for overall effect: Z = 7.39 (P < 0.00001)
Test for subgroup differences: Chi² = 87.97, df = 2 (P < 0.00001), I² = 97.7%

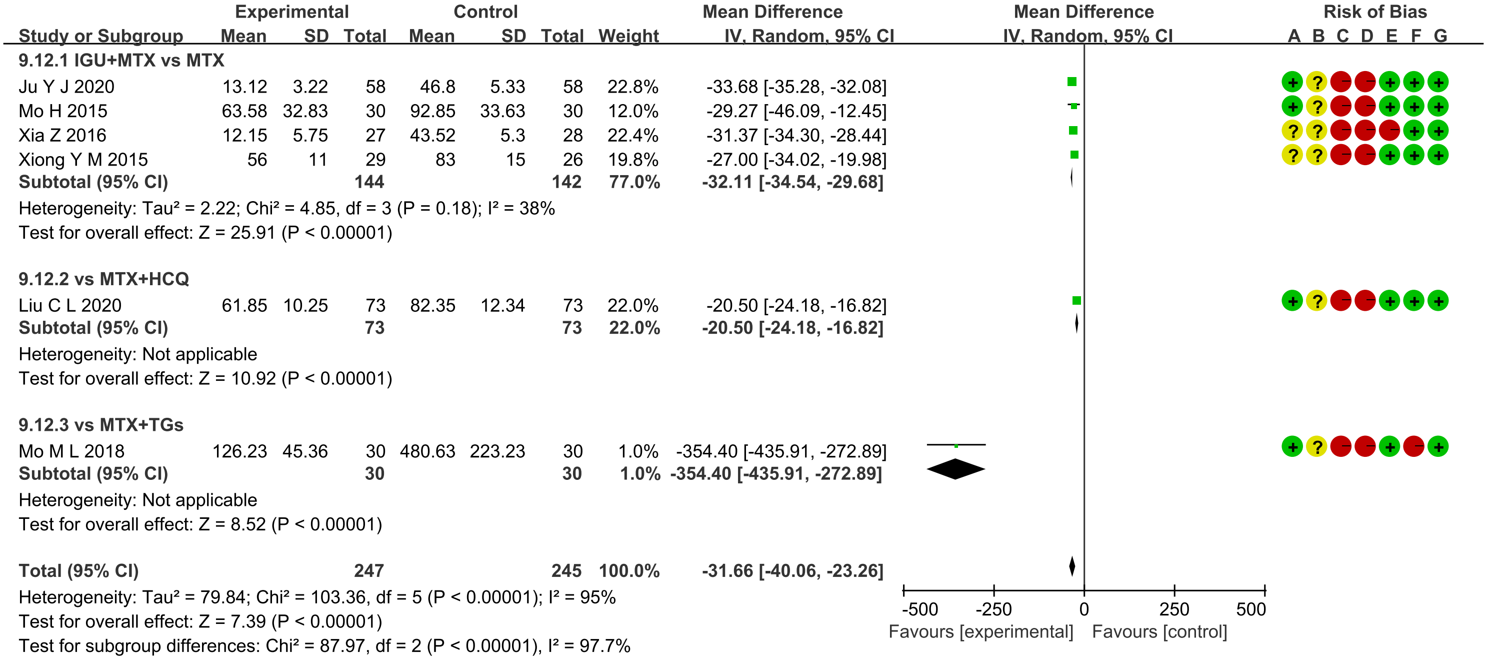

Risk of bias legend
(**A**) Random sequence generation (selection bias)
(**B**) Allocation concealment (selection bias)
(**C**) Blinding of participants and personnel (performance bias)
(**D**) Blinding of outcome assessment (detection bias)
(**E**) Incomplete outcome data (attrition bias)
(**F**) Selective reporting (reporting bias)
(**G**) Other bias
