# Supplementary figures and images for: Effectiveness and Safety of Iguratimod Monotherapy or Combined With Methotrexate in Treating Rheumatoid Arthritis: A Systematic Review and Meta-Analysis
Source: Front Pharmacol. 2022 Aug 5;13:911810. doi: 10.3389/fphar.2022.911810 (PMC9389904; doi:10.3389/fphar.2022.911810)

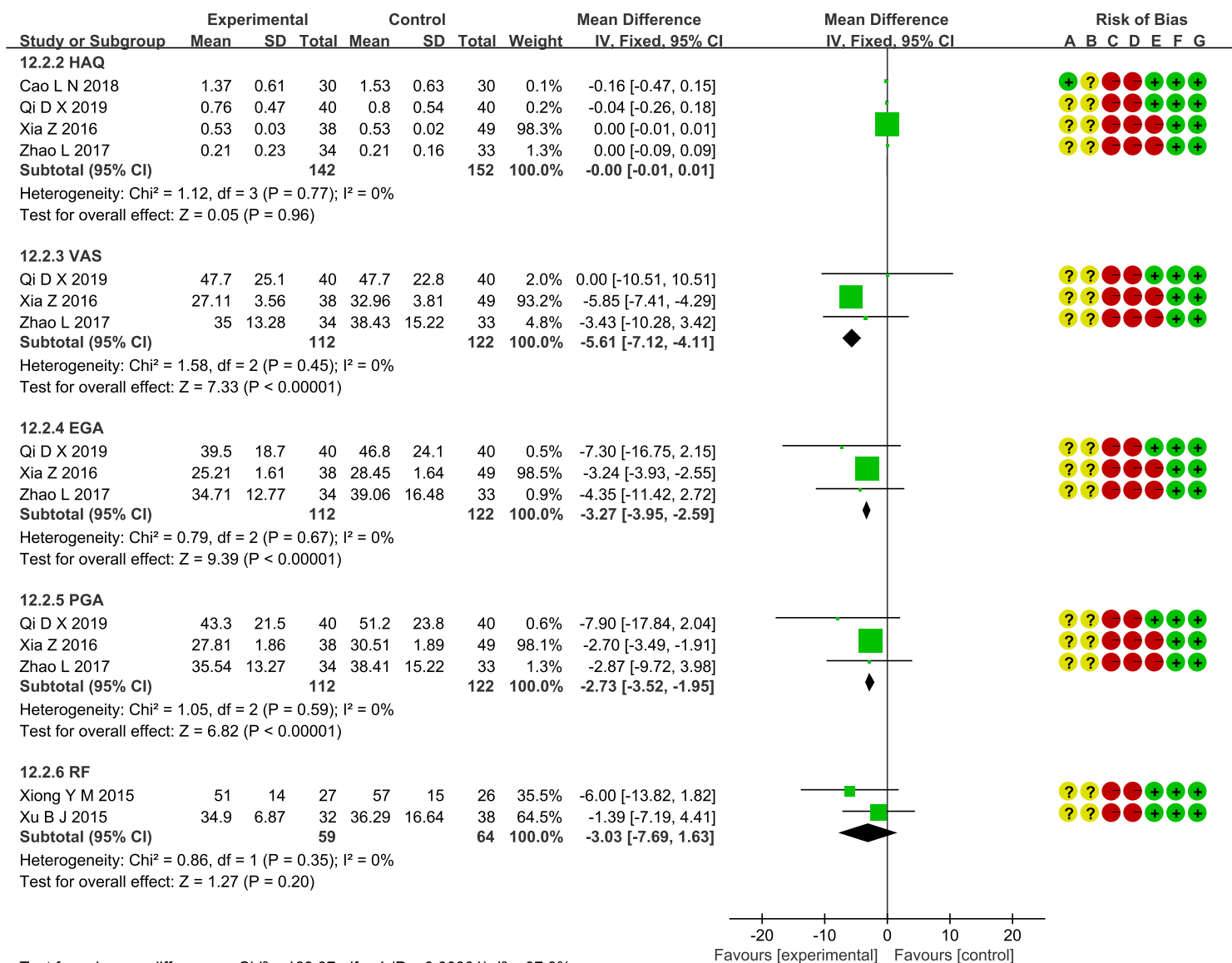

Supplement: Supplementary file 1 [file DataSheet1.ZIP › FIGURE S2.pdf]

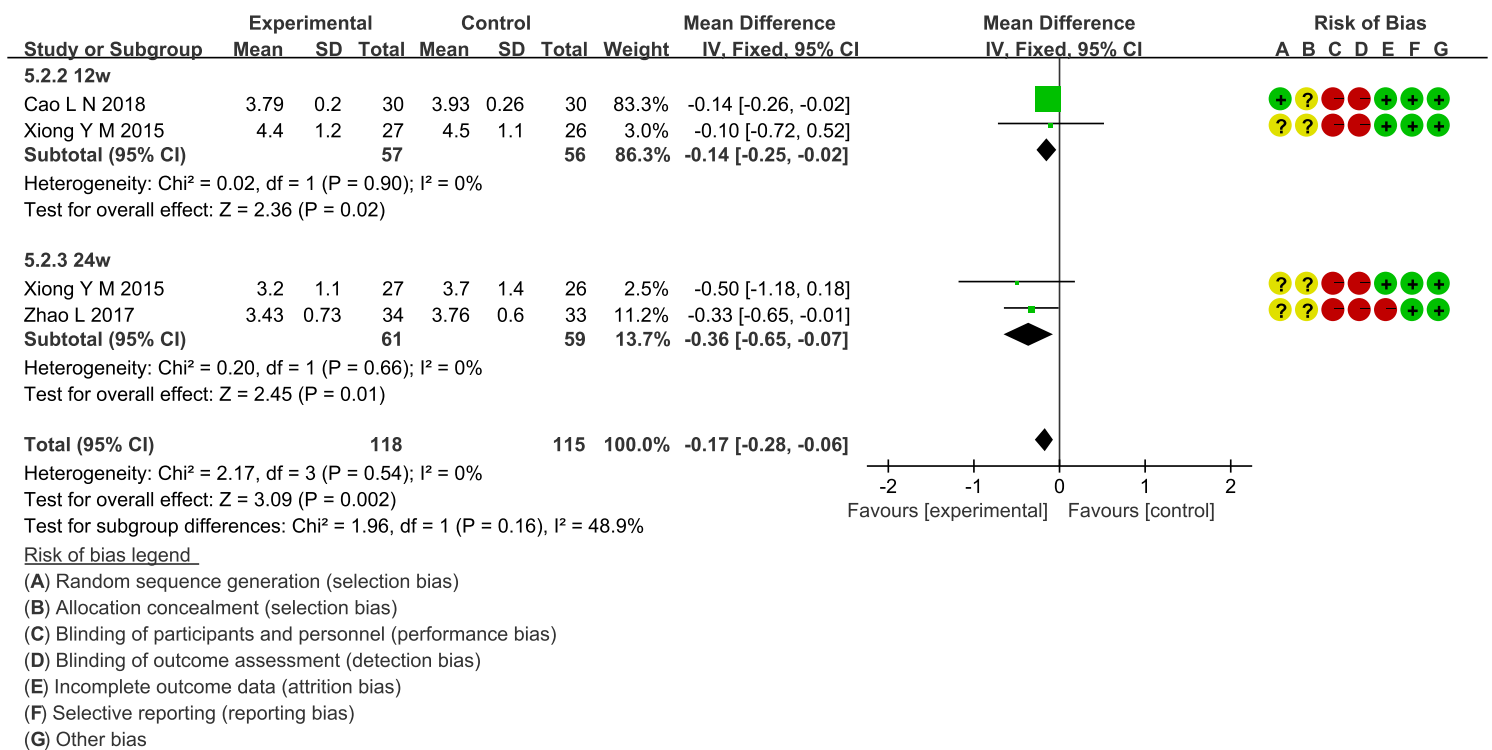

Supplement: Supplementary file 1 [file DataSheet1.ZIP › S13.pdf]

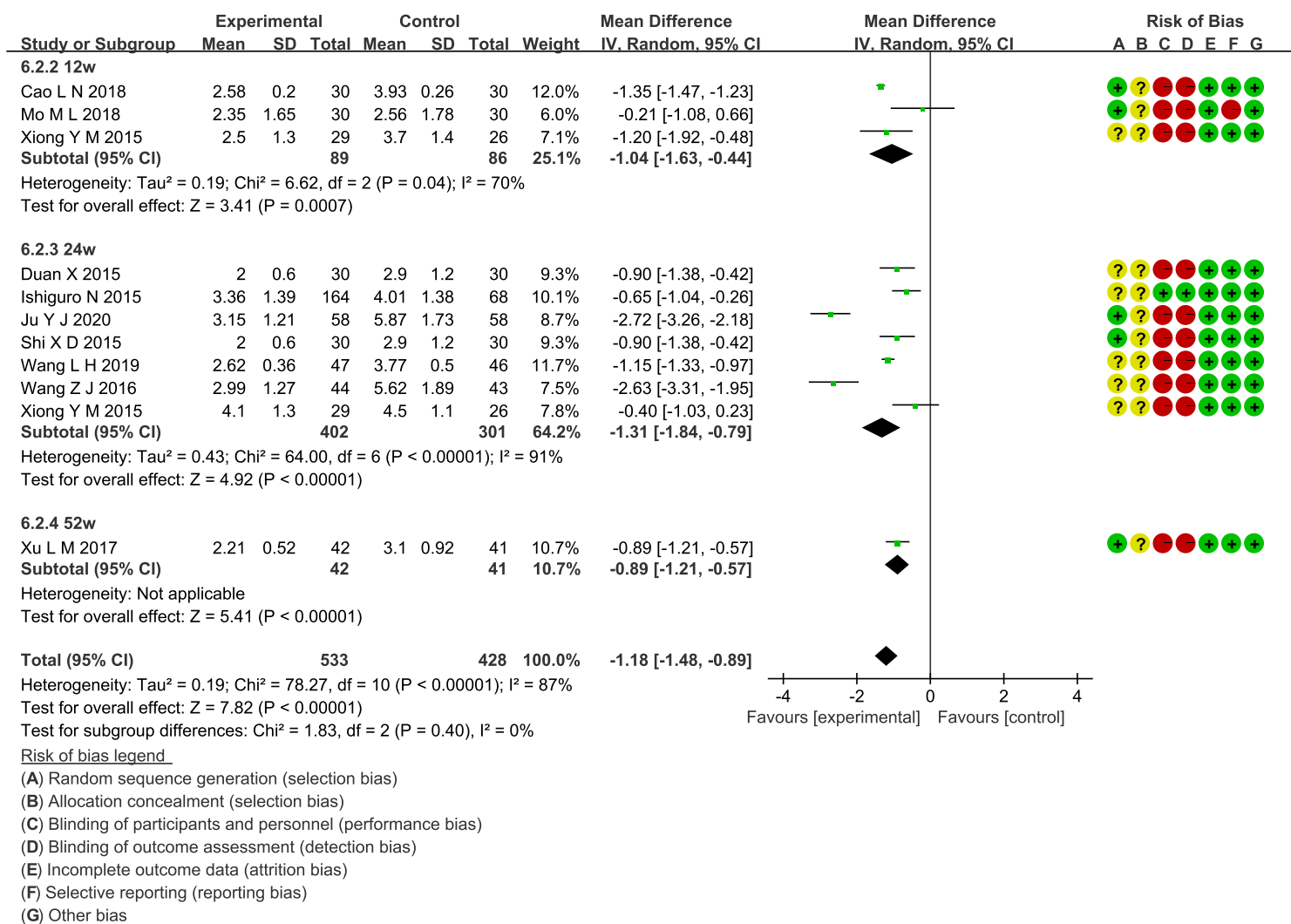

Supplement: Supplementary file 1 [file DataSheet1.ZIP › S14.pdf]

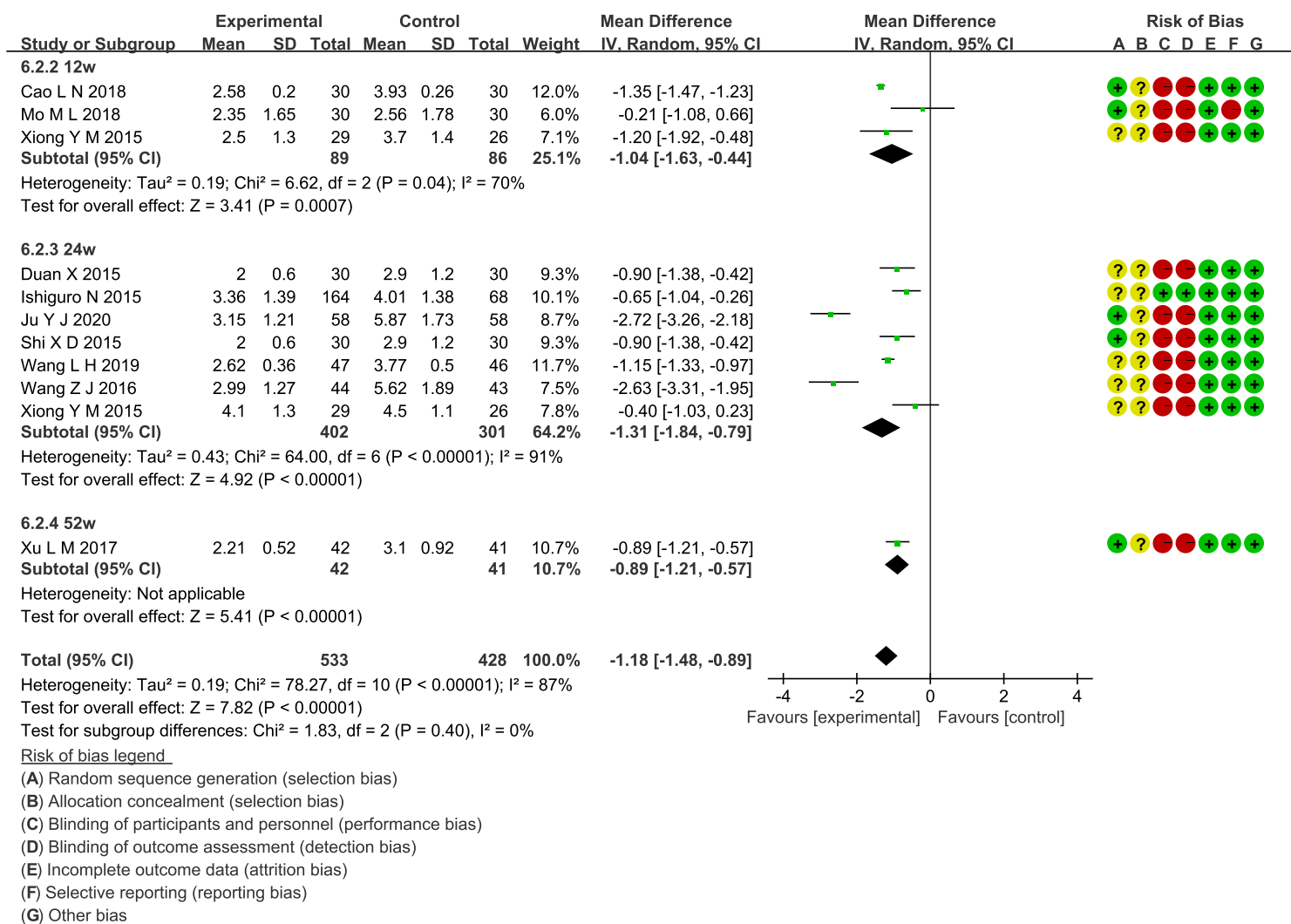

Supplement: Supplementary file 1 [file DataSheet1.ZIP › S16.pdf]

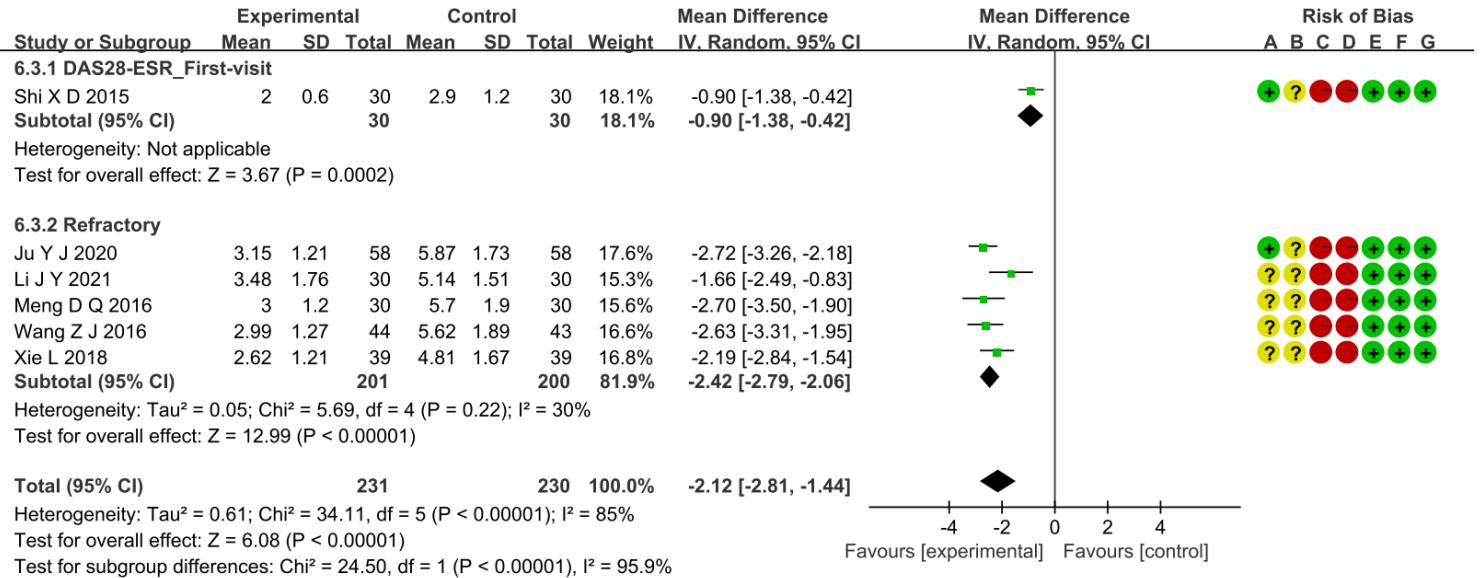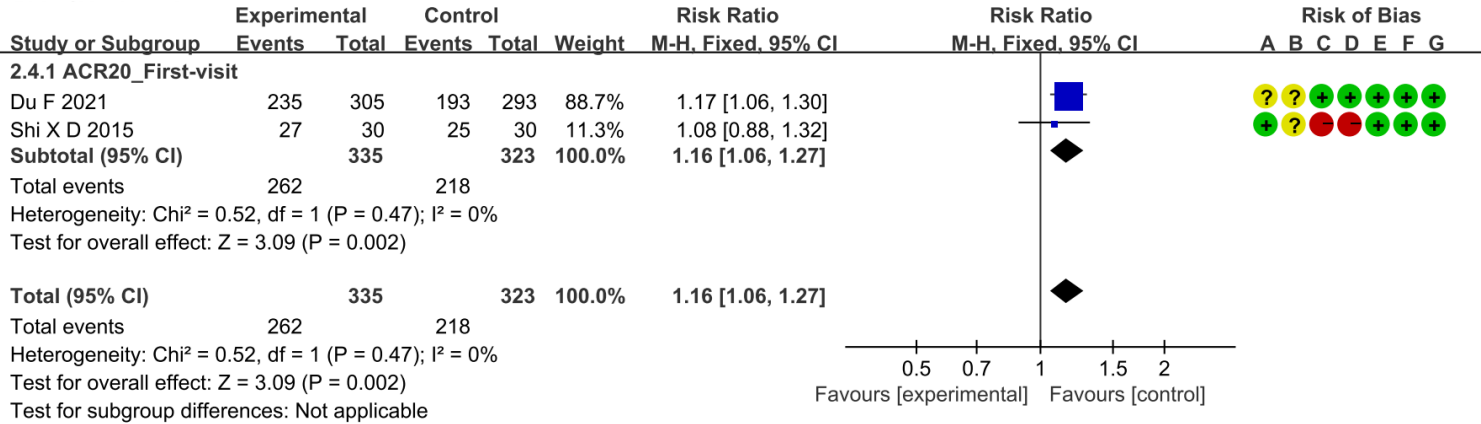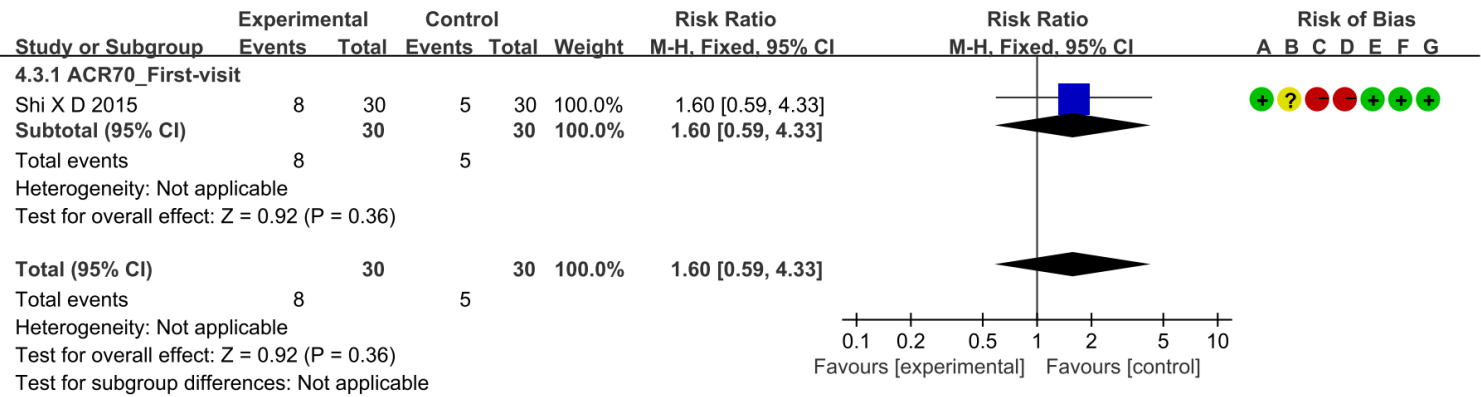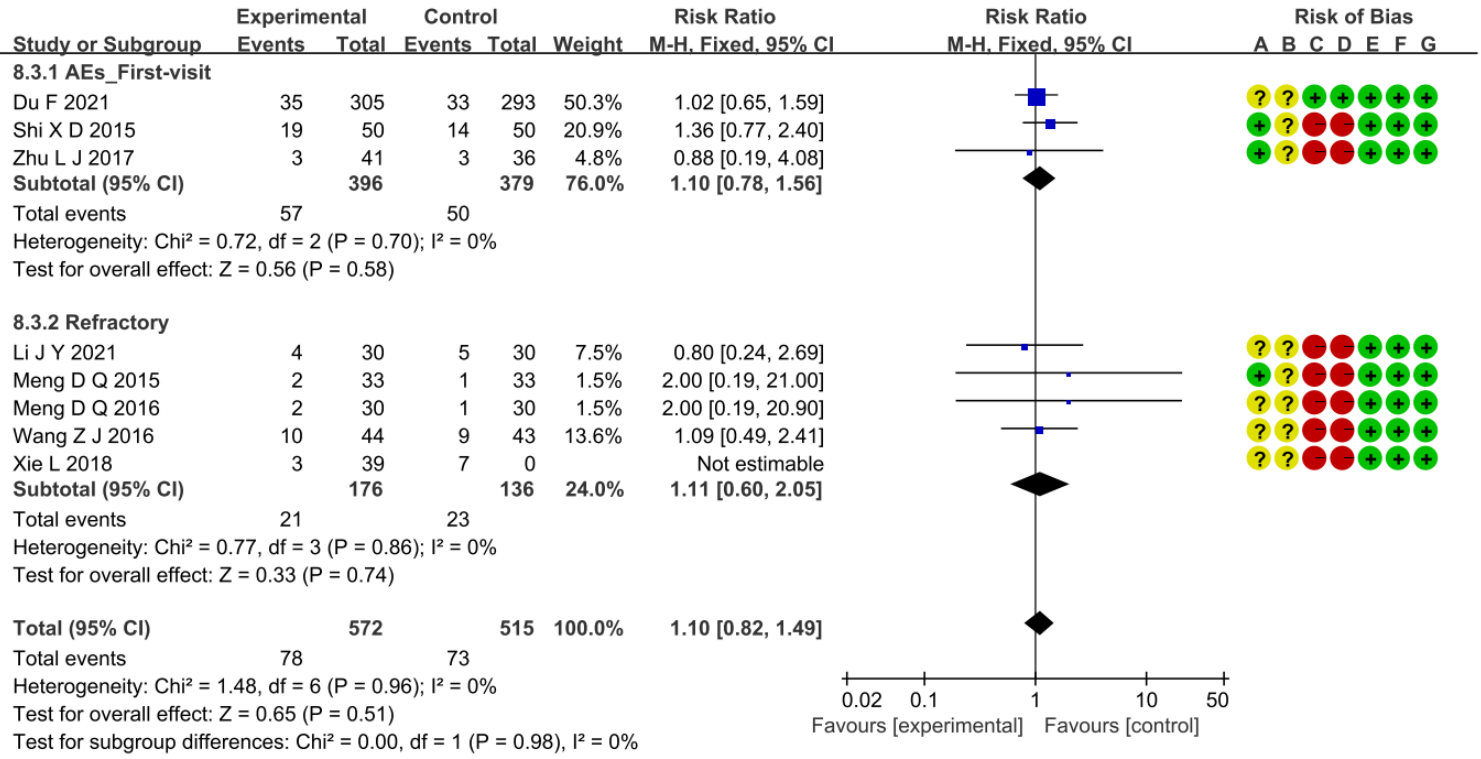

Risk of bias legend

Supplement: Supplementary file 1 [file DataSheet1.ZIP › S22.pdf]

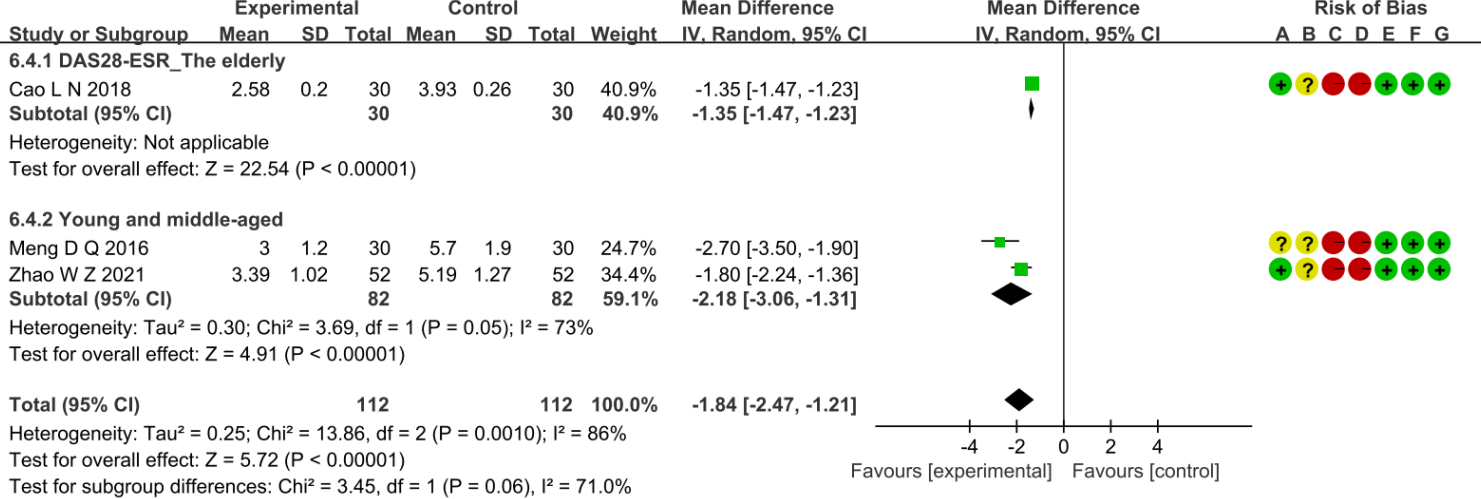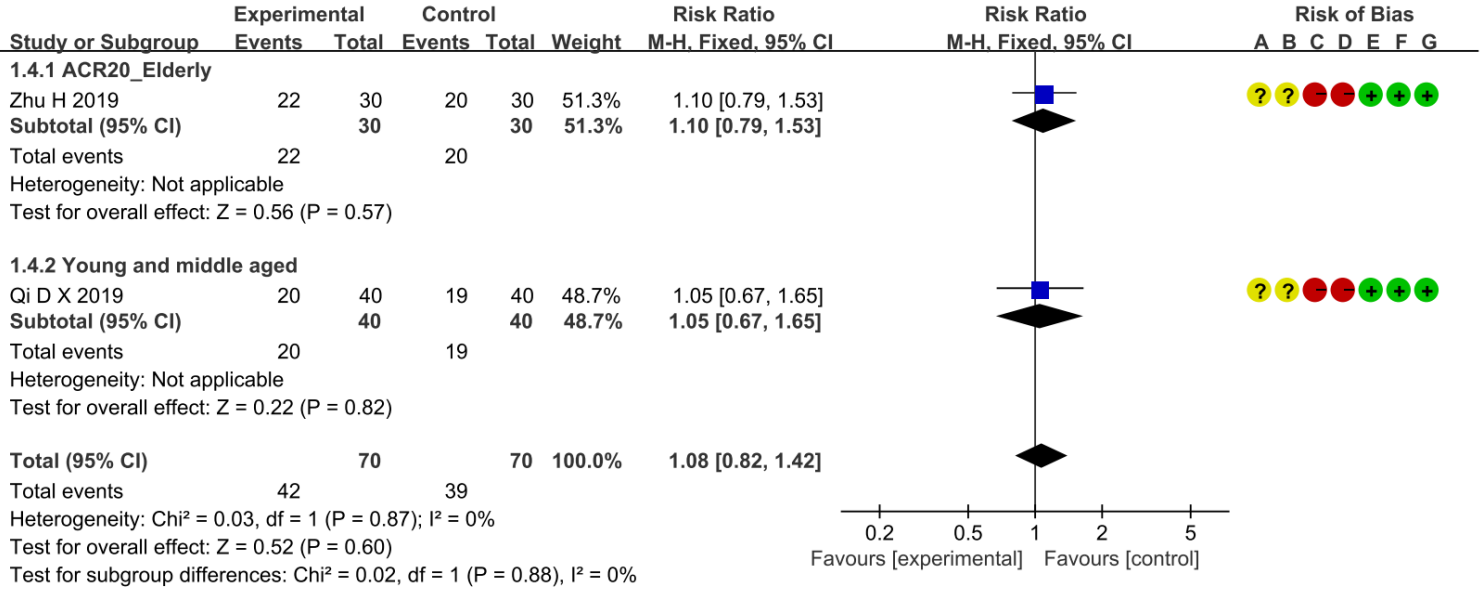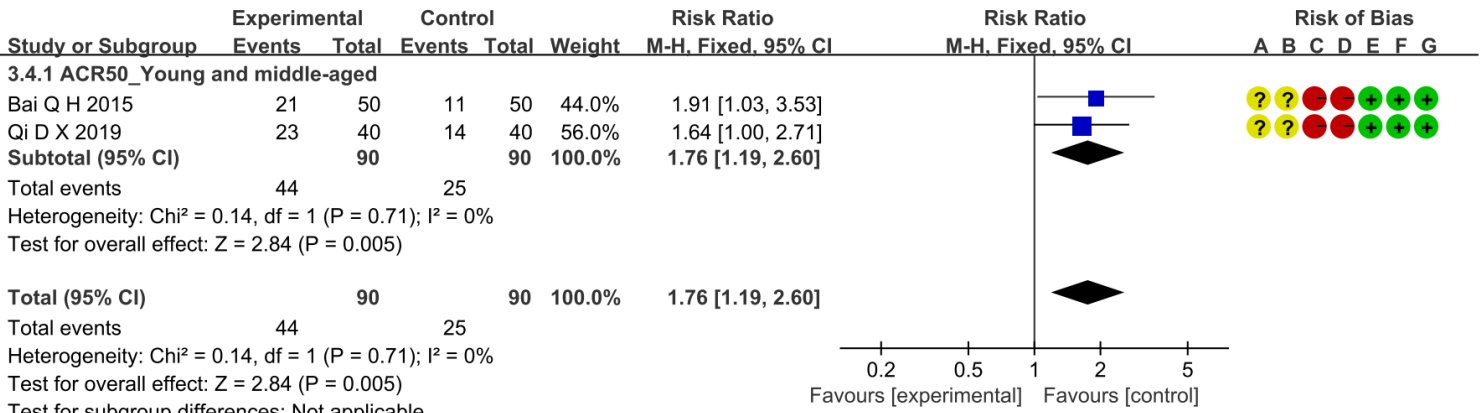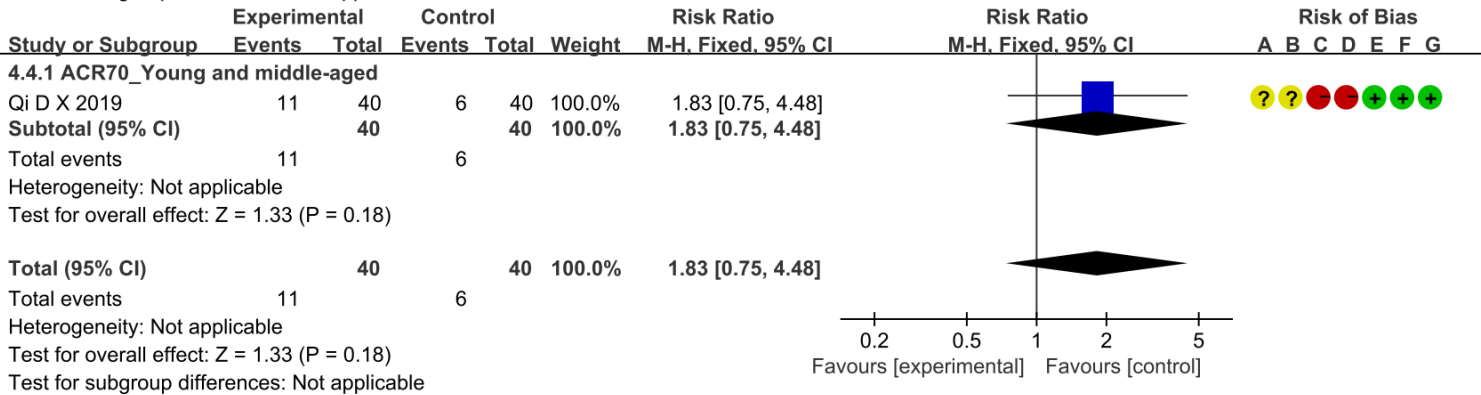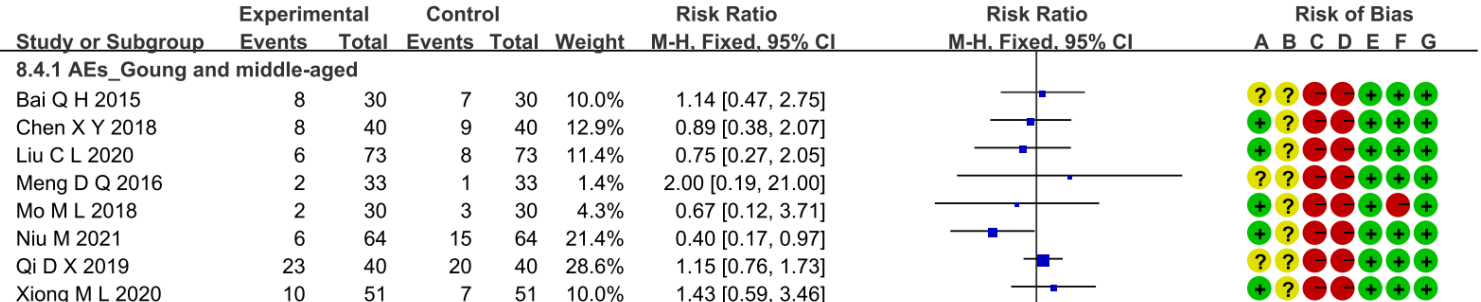

Supplement: Supplementary file 1 [file DataSheet1.ZIP › S25.pdf]

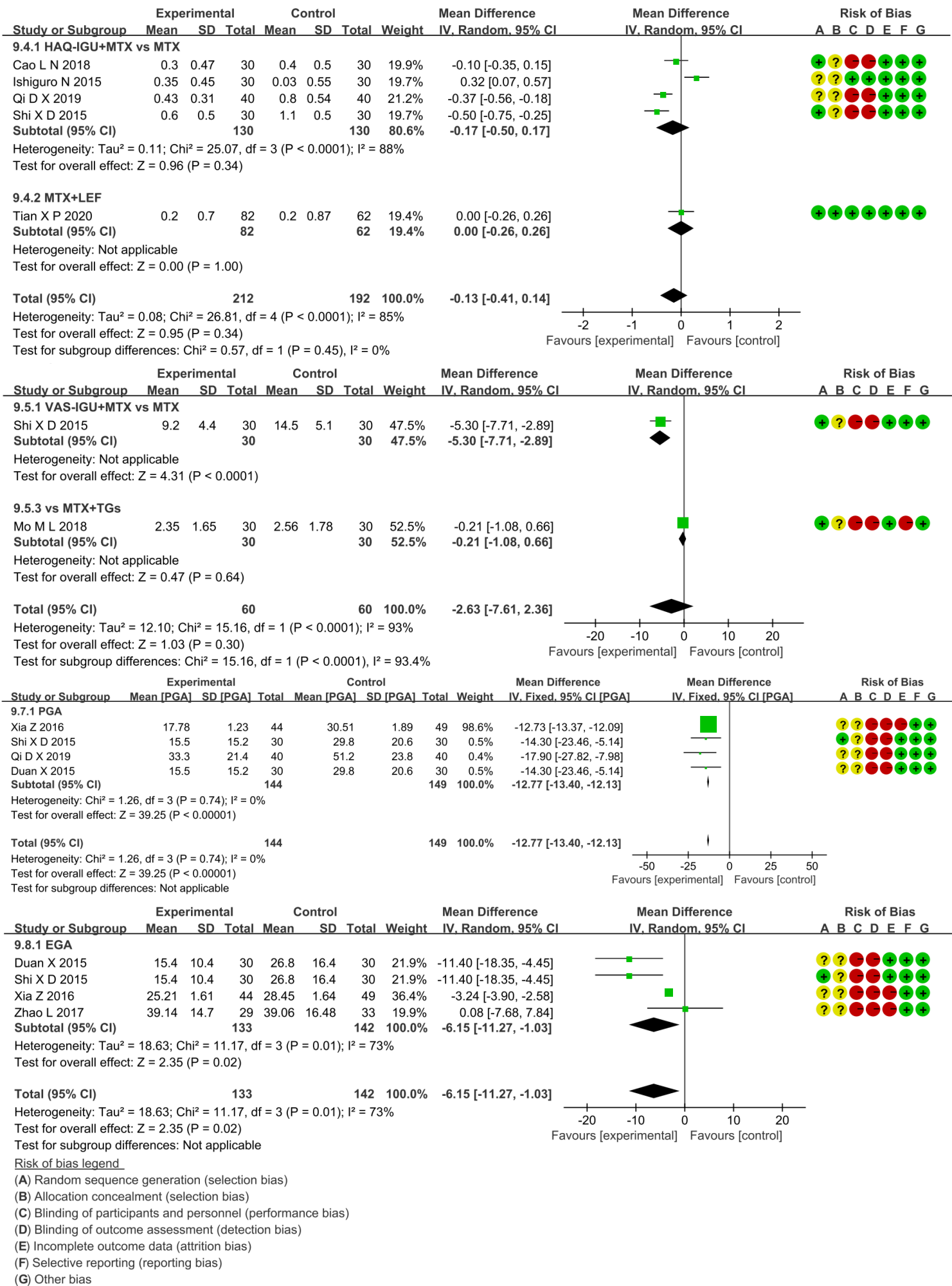

Supplement: Supplementary file 1 [file DataSheet1.ZIP › S5.pdf]
