# Supplementary material for: Effectiveness and Safety of Iguratimod Monotherapy or Combined With Methotrexate in Treating Rheumatoid Arthritis: A Systematic Review and Meta-Analysis
Source: Front Pharmacol. 2022 Aug 5;13:911810. doi: 10.3389/fphar.2022.911810 (PMC9389904; doi:10.3389/fphar.2022.911810)
Supplement: Supplementary file 2 [file Table2.DOCX]

**Supplementary Table S2.** Search Strategies

| **PubMed** | (Iguratimod OR Alamode OR T-614 OR C17H14N2O6S OR CAS 123663-49-0 OR IGU OR 3-Formylamino-7-methylsulfonylamino-6-phenoxy-4H-1-benzopyran-4-one)  AND  ("Arthritis, Rheumatoid"[Mesh]) OR (Rheumatoid arthritis[tiab])) OR (RA[tiab])  AND  (random* controlled trial [pt] OR controlled clinical trial* [pt] OR randomized [tiab] OR placebo [tiab] OR drug therapy [sh] OR random* [tiab] OR trial* [tiab] OR group* [tiab]) |
| --- | --- |
| **EMBASE** | 1 'Iguratimod'  2 'Alamode'  3 ('T-614' or 'C17H14N2O6S' or 'IGU').ti,ab.  4 1 or 2 or 3  5 'Arthritis, rheumatoid'/exp  6 'Rheumatoid arthritis'  7 ('RA').ti,ab.  8 5 or 6 or 7  9 'randomized controlled trial'  10 'single blind procedure' or 'double blind procedure'  11 'crossover procedure'  12 9 or 10 or 11  13 4 and 8 and 12 |
| **Web of Science** | (Iguratimod OR Alamode OR T-614 OR C17H14N2O6S OR CAS 123663-49-0 OR IGU OR 3-Formylamino-7-methylsulfonylamino-6-phenoxy-4H-1-benzopyran-4-one)  AND  (Arthritis, Rheumatoid OR Rheumatoid arthritis OR RA)  AND  (randomized controlled trial [pt] OR controlled clinical trial [pt] OR trial [tiab] OR clinical trials as topic [mesh: noexp] OR Clinical Trial OR random* [tiab] OR random allocation [mh] OR single-blind method [mh] OR double-blind method [mh]) |
| **Chinese Clinical Trial Registry** | Condition or disease: (艾拉莫德 OR T-614)  Other terms: (类风湿关节炎 OR 类风湿性关节炎 OR 关节炎，类风湿) |
| **Clinicaltrials.gov** | Condition or disease: Rheumatoid arthritis OR Arthritis, Rheumatoid  Other terms: (Iguratimod OR Alamode OR T-614 OR C17H14N2O6S OR CAS 123663-49-0 OR IGU OR 3-Formylamino-7-methylsulfonylamino-6-phenoxy-4H-1-benzopyran-4-one) |
| **CENTRAL** | 1 Iguratimod OR Alamode OR T-614 OR C17H14N2O6S OR IGU  2 Arthritis, rheumatoid OR Rheumatoid arthritis OR RA  3 random controlled trial OR controlled clinical trial OR randomized OR placebo OR drug therapy OR random OR trial OR group OR RCT  4 #1 AND #2 AND #3 |
| **CNKI** | ( SU='艾拉莫德' OR SU=' T-614')  AND  (SU='类风湿性关节炎' OR SU='类风湿关节炎' OR SU='关节炎，类风湿')  AND  ( SU='随机' OR SU=' RCT') |
| **Wanfang** | ('艾拉莫德' OR ' T-614')  AND  ('类风湿性关节炎' OR '类风湿关节炎')  AND  ('随机' OR ' RCT') |
| **VIP** | (M=艾拉莫德OR M= T-614)  AND  (M=类风湿性关节炎OR M=类风湿关节炎OR M=关节炎，类风湿)  AND  (M=随机OR M= RCT) |
| **CBM** | ("艾拉莫德"[常用字段:智能] OR "T-614"[常用字段:智能] OR " IGU "[常用字段:智能] OR "T-614"[常用字段:智能] OR "iguratimod "[常用字段:智能])  AND  ("类风湿性关节炎"[常用字段:智能] OR "类风湿关节炎"[常用字段:智能] OR"关节炎, 类风湿"[常用字段:智能])  AND  ("随机"[常用字段:智能] OR "RCT"[常用字段:智能]) |
